# Synthesis and Biological Evaluation of Benzo[*b*]thiophene Acylhydrazones as Antimicrobial Agents against Multidrug-Resistant *Staphylococcus aureus*

**DOI:** 10.3390/biom12010131

**Published:** 2022-01-14

**Authors:** Thibaut Barbier, Alexia Barbry, Jérémy Magand, Cédric Badiou, Floriane Davy, Anne Baudouin, Yves Queneau, Oana Dumitrescu, Gérard Lina, Laurent Soulère

**Affiliations:** 1 Univ Lyon, INSA Lyon, UCBL, Institut de Chimie et de Biochimie Moléculaires et Supramoléculaires, ICBMS, UMR 5246, CNRS, Université Lyon 1, CPE-Lyon, Bâtiment Lederer, 1 Rue Victor Grignard, 69622 Villeurbanne, France; thibaut.barbier@univ-lyon1.fr (T.B.); jeremy.magand@univ-orleans.fr (J.M.); yves.queneau@insa-lyon.fr (Y.Q.); 2 Hospices Civils de Lyon, Hôpital de la Croix Rousse-Centre de Biologie Nord, Institut des Agents Infectieux, Laboratoire de Bactériologie, Grande Rue de la Croix Rousse, 69004 Lyon, France; alexia.barbry@chu-lyon.fr (A.B.); oana.dumitrescu@chu-lyon.fr (O.D.); gerard.lina@chu-lyon.fr (G.L.); 3 Team STAPATH, CIRI, Centre International de Recherche en Infectiologie, Inserm, U1111, Université Claude Bernard Lyon 1, CNRS, UMR5308, ENS de Lyon, 69007 Lyon, France; cedric.badiou@univ-lyon1.fr (C.B.); floriane.davy@univ-lyon1.fr (F.D.); 4 Centre Commun de RMN, CNRS, Université Lyon 1, CPE-Lyon, Bâtiment Lederer, 1 Rue Victor Grignard, 69622 Villeurbanne, France; anne.baudouin@univ-lyon1.fr

**Keywords:** benzothiophene, acylhydrazone, *Staphylococcus aureus*, antibacterial, MRSA

## Abstract

The benzo[*b*]thiophene nucleus and the acylhydrazone functional group were combined to prepare three new series of compounds for screening against *Staphylococcus aureus*. The reaction of substituted benzo[*b*]thiophene-2-carboxylic hydrazide and various aromatic or heteroaromatic aldehydes led to a collection of 26 final products with extensive structural diversification on the aromatic ring and on position 6 of the benzo[*b*]thiophene nucleus. The screening lead to the identification of eight hits, including (*E*)-6-chloro-*N*’-(pyridin-2-ylmethylene)benzo[*b*]thiophene-2-carbohydrazide (**II.b**), a non-cytotoxic derivative showing a minimal inhibitory concentration of 4 µg/mL on three *S. aureus* strains, among which were a reference classical strain and two clinically isolated strains resistant to methicillin and daptomycin, respectively.

## 1. Introduction

Antimicrobial resistance (AMR) is a public health issue that will continue to worsen in the years to come. At least 700,000 deaths are attributed each year to drug-resistant pathogens, but this could rise to 10,000,000 deaths per year by 2050 [1,2,3]. Among those pathogens, some are widely spread, such as vancomycin-resistant *Enterococcus faecium* or methicillin-resistant *Staphylococcus aureus* (MRSA) [4]. The development of new antibiotics targeting antibioresistant bacteria is therefore critical. Substituted benzo[*b*]thiophenes are interesting compounds in medicinal chemistry [5] that display a broad range of activity including antimicrobial [6,7], anticancer [8], anti-diabetic [9], anti-depressant [10], anti-inflammatory and analgesic agents [11,12]. As part of an ongoing research program aiming at the discovery of new potential antibiotics targeting multidrug-resistant *Staphylococcus aureus* strains, we combined the benzo[*b*]thiophene nucleus with the acylhydrazone functional group, which is also relevant in bioactive molecules design [13]. We focused our efforts on the synthesis and biological evaluation of a collection of acylhydrazones built from various aromatic or heteroaromatic aldehydes and benzo[*b*]thiophene-2-carboxylic hydrazide, readily accessible from benzo[*b*]thiophene-2-carboxylic acid. This sequence allowed an easy structural diversification of both the heteroaromatic benzo[*b*]thiophene nucleus and the aromatic system. The purpose was then to screen the collection of benzo[*b*]thiophene-2-acylhydrazones for identifying new hits against drug-resistant *S. aureus* strains.

## 2. Materials and Methods

### 2.1. Chemistry

#### 2.1.1. General Information

All commercial materials were used as received without further purification. Flash chromatography was carried out using Macherey-Nagel Kieselgel 60 M silica. Analytical thin-layer chromatography was realized using aluminum-backed plates coated with Macherey-Nagel Kieselgel 60 XtraSIL G/UV254 and were visualized under UV light (at 254 nm or 365 nm) or stained using ninhydrin. Nuclear magnetic resonance (NMR) spectra were recorded on Bruker AV300, Bruker AV400 or Bruker AV500 spectrometers, operating at 300 MHz, 400 MHz and 500 MHz, respectively, for the proton (^1^H) NMR and at 75 MHz, 100 MHz and 125 MHz, respectively, for the carbon (^13^C) NMR. Chemical shifts were reported in parts per million (ppm) on a scale relative to residual solvent signals. Multiplicities are abbreviated as: s, singlet; d, doublet; t, triplet; q, quadruplets; dd, doublet of doublets; dt, doublet of triplets; td, triplet of doublets; ddd, doublet of doublet of doublets; m, multiplet. Coupling constants were measured in Hertz (Hz). Copies of NMR spectra of new products are available in Supplementary Materials (Figure S1). High-resolution mass spectra (HRMS) and low-resolution mass spectra were obtained by the Centre Commun de Spectrométrie de Masse (CCSM), University of Lyon 1, Lyon, France. LogP calculations were performed using Molinspiration Cheminformatics free web services, https://www.molinspiration.com (accessed on 13 January 2022), Slovensky Grob, Slovakia.

#### 2.1.2. General Procedure for Ethyl 6-halogenobenzo[*b*]thiophene-2-carboxylate (**4**) 

Under a dried and inert atmosphere (N_2_), a solution of 4-chloro-2-fluorobenzaldehyde (14.9 mmol, 1 eq.), ethyl thioglycolate (16.5 mmol, 1.1 eq.) and triethylamine (45 mmol, 3 eq.) in anhydrous DMSO (20 mL) was stirred at 80 °C for 2 h and at room temperature overnight. The mix was then poured into 800 mL of ice/water and stirred vigorously. After 1 h of digestion, the formed solid was filtered, washed with water, and dried by suction. (Adapted from Fedi et al. 2007 [14])

##### Ethyl 6-chlorobenzo[*b*]thiophene-2-carboxylate (**4a**)

Yellow crystals (3.446 g, 96% yield). ^1^H NMR (300 MHz, DMSO-*d*_6_) δ 8.25 (d, *J* = 2.0 Hz, 1H), 8.21 (s, 1H), 8.04 (d, *J* = 8.6 Hz, 1H), 7.52 (dd, *J* = 8.6, 2.0 Hz, 1H), 4.35 (q, *J* = 7.1 Hz, 2H), 1.33 (t, *J* = 7.1 Hz, 3H). (Spectrum in accordance with patent WO2018/122232 [15]).

##### Ethyl 6-fluorobenzo[*b*]thiophene-2-carboxylate (**4b**)

No crystallization occurred in water, and the residue was purified by chromatography (eluent: pentane/Et_2_O 10:1). White powder (710 mg, 21% yield). ^1^H NMR (300 MHz, DMSO-*d*_6_) δ 8.20 (s, 1H), 8.07 (dd, *J* = 9.1, 5.4 Hz, 1H), 7.99 (dd, *J* = 9.1, 2.4 Hz, 1H), 7.37 (td, *J* = 9.1, 2.4 Hz, 1H), 4.35 (q, *J* = 7.1 Hz, 2H), 1.33 (t, *J* = 7.1 Hz, 3H). (Spectrum in accordance with Cheng et al. [16]).

#### 2.1.3. Procedure for Ethyl 6-(trifluoromethyl)benzo[*b*]thiophene-2-carboxylate (**4c**) 

Under a dried and inert atmosphere (N_2_), a solution of 2-fluoro-4-(trifluoromethyl)benzaldehyde (5.20 mmol, 1 eq.), ethyl thioglycolate (6.20 mmol, 1.2 eq.) and K_2_CO_3_ (5.70 mmol, 1.1 eq.) in anhydrous DMF (10 mL) was stirred at 60 °C for 2 h. The mix was then diluted with water (20 mL), extracted with Et_2_O, dried over Na_2_SO_4_ and concentrated under vacuum. The residue was then recrystallized in MeOH and filtered. (Adapted from patent WO2018/122232 [15]).

##### Ethyl 6-(trifluoromethyl)benzo[*b*]thiophene-2-carboxylate (**4c**)

White powder (814 mg, 57% yield). ^1^H NMR (300 MHz, Chloroform-*d*) δ 8.16 (q, *J* = 0.8 Hz, 1H), 8.10 (d, *J* = 0.8 Hz, 1H), 7.98 (dt, *J* = 8.4, 0.8 Hz, 1H), 7.63 (dd, *J* = 8.4, 1.6 Hz, 1H), 4.43 (q, *J* = 7.1 Hz, 2H), 1.43 (t, *J* = 7.1 Hz, 3H). (Spectrum in accordance with Tan et al. [17]).

#### 2.1.4. Procedure for Ethyl 6-chlorobenzofuran-2-carboxylate (**4d**) 

A solution of 4-chlorosalicylaldehyde (6.39 mmol, 1 eq.), ethyl bromomalonate (13.03 mmol, 2.04 eq.) and K_2_CO_3_ (19.74 mmol, 3.09 eq.) in 2-butanone (6 mL) was stirred at 90 °C for 7 h, then at room temperature for 18 h. The mixture was then diluted with EtOAc, neutralized with HCl 1 N and extracted. The organic phase was washed with saturated NaHCO_3_ and brine, dried over Na_2_SO_4_ and concentrated under vacuum. The residue was then purified by chromatography (eluent: pentane/Et_2_O 9:1). (Adapted from Patent US2015315198 [18]).

##### Ethyl 6-chlorobenzofuran-2-carboxylate (**4d**)

White powder (611 mg, 43% yield). ^1^H NMR (300 MHz, Chloroform-*d*) δ 7.62–7.57 (m, 2H), 7.49 (d, *J* = 1.3 Hz, 1H), 7.29 (dd, *J* = 8.5, 1.3 Hz, 1H), 4.44 (q, *J* = 7.1 Hz, 2H), 1.43 (t, *J* = 7.1 Hz, 3H). (Spectrum in accordance with Chen et al. 2017 [19]).

#### 2.1.5. General Procedure for Benzo[*b*]thiophene-2-carboxylic Acids (**1**) 

To a solution of ethyl 6-chlorobenzo[*b*]thiophene-2-carboxylate (14.1 mmol, 1 eq.) in EtOH (15 mL) was added a solution of NaOH 3N (28.2 mmol, 2 eq.). The solution was stirred at room temperature overnight, concentrated under vacuum, diluted with H_2_O (75 mL), acidified with HCl 1 N, extracted with EtOAc, dried over Na_2_SO_4_ and concentrated under vacuum. (Adapted from Patent WO2018/122232 [15]).

##### 6-Chlorobenzo[*b*]thiophene-2-carboxylic acid (**1b**)

White powder (2.616 g, 87% yield). ^1^H NMR (300 MHz, DMSO-*d*_6_) δ 13.57 (bs, 1H), 8.23 (d, *J* = 2.0 Hz, 1H), 8.12 (s, 1H), 8.02 (d, *J* = 8.6 Hz, 1H), 7.50 (dd, *J* = 8.6, 2.0 Hz, 1H); ^13^C NMR (101 MHz, DMSO-*d*_6_) δ 163.48, 142.6, 137.59, 135.82, 132.07, 129.89, 127.20, 125.84, 122.60.

##### 6-Fluorobenzo[*b*]thiophene-2-carboxylic acid (**1c**)

Yellowish powder (295 mg, 75% yield). ^1^H NMR (300 MHz, DMSO-*d_6_*) δ 13.46 (s, 1H), 8.12 (d, *J* = 0.8 Hz, 1H), 8.05 (dd, *J =* 9.0, 5.4 Hz, 1H), 7.98 (dd, *J =* 9.4, 2.6 Hz, 1H), 7.35 (td, *J =* 9.0, 2.6 Hz, 1H); ^13^C NMR (101 MHz, DMSO-*d*_6_) δ 163.48 161.43 (d, *J* = 245.2 Hz), 142.76 (d, *J* = 11.3 Hz), 135.75, 134.96 (d, *J* = 3.9 Hz), 129.99, 127.70 (d, *J* = 9.6 Hz), 114.48 (d, *J* = 24.8 Hz), 109.13 (d, *J* = 26.2 Hz). (Spectrum in accordance with Cai et al. [20]).

##### 6-(Trifluoromethyl)benzo[b]thiophene-2-carboxylic acid (1d)

White powder (400 mg, 89% yield). ^1^H NMR (300 MHz, DMSO-*d*_6_) δ 8.59 (q, *J* = 0.8 Hz, 1H), 8.10 (d, *J* = 0.8 Hz, 1H), 7.98 (dt, *J* = 8.5, 0.8 Hz, 1H), 7.75 (dd, *J* = 8.5, 1.3 Hz, 1H). (Spectrum in accordance with Tan et al. [17]).

##### 6-Chlorobenzofuran-2-carboxylic acid (**1e**)

White powder (378 mg, 86% yield). ^1^H NMR (300 MHz, DMSO-*d*_6_) δ 7.90 (d, *J* = 1.9 Hz, 1H), 7.80 (d, *J* = 8.4 Hz, 1H), 7.68 (d, *J* = 1.0 Hz, 1H), 7.40 (dd, *J* = 8.4, 1.9 Hz, 1H). (Spectrum in accordance with Gensini et al. [21]).

#### 2.1.6. General Procedure for Tert-butyl 2-(benzo[*b*]thiophene-2-carbonyl)hydrazine-1-carboxylates (**2**)

Under a dried and inert atmosphere (N_2_), a solution of benzothiophene-2-carboxylic acid (11 mmol, 1.1 eq.) and *tert*-butyl carbazate (10 mmol, 1.0 eq.) in anhydrous DCM (40 mL) was cooled to 0 °C. Then, DMAP (1.3 mmol, 0.13 eq.) was added to the mixture. DCC (12 mmol, 1.2 eq.) in anhydrous DCM (10 mL) was added dropwise. The mixture was then stirred at room temperature for 24 h, filtered on Celite^®^ and washed with DCM. The filtrate was then concentrated, and the viscous yellow oil was purified by chromatography (eluent: pentane/Et_2_O, 1:1).

##### Tert-butyl-2-(benzo[*b*]thiophene-2-carbonyl)hydrazine-1-carboxylate (**2a**)

White powder (2.630 g, 88% yield). ^1^H NMR (300 MHz, Chloroform-*d*) δ 8.77 (s, 1H), 7.83 (s, 1H), 7.75 (t, *J* = 6.9 Hz, 2H), 7.47–7.31 (m, 2H), 6.82 (s, 1H), 1.50 (s, 9H); ^13^C NMR (75 MHz, Chloroform-*d*) δ 162.4, 156.2, 141.1, 138.9, 135.3, 126.6, 126.5, 125.4, 124.8, 122.4, 82.3, 28.1.

##### Tert-butyl-2-(6-chlorobenzo[*b*]thiophene-2-carbonyl)hydrazine-1-carboxylate (**2b**)

Acid **1b** was used. White powder (1.435 g, 79% yield). ^1^H NMR (300 MHz, Chloroform-*d*) δ 8.73 (s, 1H), 7.75 (s, 1H), 7.71 (s, 1H), 7.67 (d, *J* = 8.6 Hz, 1H), 7.33 (dd, *J* = 8.6, 1.9 Hz, 1H), 6.72 (s, 1H), 1.51 (s, 9H); ^13^C NMR (101 MHz, Acetone-*d*_6_) δ 162.42, 156.46, 142.96, 139.20, 138.93, 132.97, 127.41, 126.55, 125.83, 123.03, 80.78, 28.39.

##### Tert-butyl-2-(6-fluorobenzo[*b*]thiophene-2-carbonyl)hydrazine-1-carboxylate (**2c**)

Acid **1c** was used. White powder (305 mg, 42% yield). ^1^H NMR (300 MHz, Chloroform-*d*) δ 8.41 (s, 1H), 7.79 (s, 1H), 7.75 (dd, *J* = 8.9, 5.2 Hz, 1H), 7.45 (d, *J* = 8.9 Hz, 1H), 7.14 (td, *J* = 8.9, 2.4 Hz, 1H), 6.67 (s, 1H), 1.51 (s, 9H); ^13^C NMR (101 MHz, DMSO-*d*_6_) δ 162.19, 160.52 (d, *J* = 153.3 Hz), 155.39, 141.54 (d, *J* = 10.8 Hz), 137.52 (d, *J* = 3.0 Hz), 135.91, 127.24 (d, *J* = 9.4 Hz), 125.14, 114.27 (d, *J* = 24.4 Hz), 108.98 (d, *J* = 26.2 Hz), 79.49, 28.08.

##### Tert-butyl-2-(6-(trifluoromethyl)benzo[*b*]thiophene-2-carbonyl)hydrazine-1-carboxylate (**2d**)

Acid **1d** was used. White powder (213 mg, 64% yield). ^1^H NMR (300 MHz, Chloroform-*d*) δ 9.11 (s, 1H), 7.94 (s, 1H), 7.82 (d, *J* = 8.7 Hz, 1H), 7.80 (s, 1H), 7.56 (dd, *J* = 8.7, 1.7 Hz, 1H), 6.76 (s, 1H), 1.53 (s, 9H); ^13^C NMR (75 MHz, Acetone-*d*_6_) δ 162.23, 156.45, 142.86, 142.07, 141.57, 128.48 (q, *J* = 32.3 Hz), 127.07, 125.77, 125.4 (q, *J* = 271.5 Hz), 122.16 (q, *J* = 3.5 Hz), 121.22 (q, *J* = 4.5 Hz), 80.88, 28.40.

##### Tert-butyl-2-(6-chlorobenzofuran-2-carbonyl)hydrazine-1-carboxylate (**2e**)

Acid **1e** was used. White powder (281 mg, 78% yield). ^1^H NMR (300 MHz, Acetone-*d*_6_) δ 9.74 (s, 1H), 8.17 (s, 1H), 7.77 (d, *J* = 8.4 Hz, 1H), 7.65–7.53 (m, 2H), 7.35 (dd, *J* = 8.4, 1.8 Hz, 1H), 1.45 (s, 9H); ^13^C NMR (75 MHz, Acetone-*d*_6_) δ 158.77, 156.30, 155.75, 149.81, 133.17, 127.08, 125.38, 124.68, 112.96, 111.27, 80.77, 28.39.

#### 2.1.7. General Procedure for N-Acylhydrazones Derivatives (**I**, **II** and **III**)

**Step 1:** A solution of *tert*-butyl 2-(benzo[*b*]thiophene-2-carbonyl)hydrazine-1-carboxylate (1 mmol, 1 eq.) and TFA (20 mmol, 20 eq.) in anhydrous DCM (3 mL) was stirred at room temperature for 18 h. The mixture was co-evaporated with toluene to produce a white solid, which was engaged as crude material in the next step.

**Step 2:** The crude material was diluted in MeOH (10 mL) before the addition of the corresponding substituted benzaldehyde (2 mmol, 2 eq.) at room temperature. Reflux for 2 h was performed, and the final compound crystallized from the reaction mixture. Then, the reaction mixture was cooled to 0 °C, and the solid was filtered off and washed with cold MeOH.

##### (*E*)-*N*’-(Benzo[*d*][1,3]dioxol-5-ylmethylene)benzo[*b*]thiophene-2-carbohydrazide (**I.a**)

Light-yellow solid (127 mg, 52% yield). ^1^H NMR (300 MHz, DMSO-*d*_6_) δ 12.05 (s, 0.5H), 11.94 (s, 0.5H), 8.40 (d, *J* = 7.6 Hz, 1H), 8.23 (s, 0.5H), 8.16–7.97 (m, 2.5H), 7.69–7.37 (m, 2.5H), 7.32 (s, 0.5H), 7.28–7.18 (m, 1H), 7.03 (t, *J* = 7.1 Hz, 1H), 6.13 (s, 1H), 6.11 (s, 1H); ^13^C NMR (100 MHz, DMSO-*d*_6_) δ 161.9, 158.5, 149.7, 149.6, 148.5, 148.4, 145.0, 143.5, 140.8, 139.5, 138.9, 137.7, 133.7, 132.3, 129.0, 128.8, 127.2, 127.0, 126.0, 125.8, 125.5, 125.2, 124.2, 124.0, 123.3, 123.1, 109.1, 109.0, 106.0, 105.7, 102.1; HRMS (ESI) *m*/*z*: calcd. for C_17_H_13_N_2_O_3_S [M + H]^+^ 325.0641, found 325.0641.

##### (*E*)-*N*’-(4-(Dimethylamino)benzylidene)benzo[*b*]thiophene-2-carbohydrazide (**I.b**)

Yellow solid (99 mg, 45% yield). ^1^H NMR (300 MHz, DMSO-*d*_6_) δ 11.85 (s, 0.5H), 11.78 (s, 0.5H), 8.40 (s, 0.5H), 8.32 (s, 0.5H), 8.20 (s, 0.5H), 8.13–7.94 (m, 2.5H), 7.68 (d, *J* = 8.7 Hz, 1H), 7.57 (d, *J* = 8.7 Hz, 1H), 7.52–7.41 (m, 2H), 6.82 (d, *J* = 8.7 Hz, 1H), 6.77 (d, *J* = 8.7 Hz, 1H), 3.00 (s, 3H), 2.99 (s, 3H); MS (ESI) *m*/*z* = 324.1 [M + H]^+^, 346.1 [M + Na]^+^.

##### (*E*)-*N*’-(Pyridin-2-ylmethylene)benzo[*b*]thiophene-2-carbohydrazide (**I.c**)

Intermediate **2a** (938 mg) was deprotected under the reported acidic conditions (TFA) to produce, after an aqueous workup with NaHCO_3_ and extractions with EtOAc, a white solid (615 mg), which was directly engaged in the presence of 2-pyridinecarboxaldehyde (6.40 mmol, 2.0 eq.) in MeOH (20 mL). After 2 h reflux, the reaction mixture was concentrated in vacuo to give a brown viscous oil, which was purified by recrystallization in EtOAc to give product. White powder (292 mg, 32% yield). ^1^H NMR (300 MHz, DMSO-*d*_6_) δ 12.34 (s, 1H, 0.5H), 12.26 (s, 0.5H), 8.65 (d, *J* = 4.5 Hz, 1H), 8.49 (d, *J* = 10.6 Hz, 1H), 8.29 (s, 0.5H), 8.23 (s, 1H), 8.16–7.85 (m, 3.5H), 7.61–7.37 (m, 3H); ^13^C NMR (100 MHz, DMSO-*d*_6_) δ 162.3, 158.9, 153.5, 153.4, 150.1, 148.7, 145.6, 143.4, 140.9, 139.5, 138.4, 137.8, 137.6, 137.4, 133.5, 132.6, 127.3, 127.2, 126.6, 126.1, 126.0, 125.6, 125.3, 125.0, 123.3, 123.1, 120.8, 120.5; HRMS (ESI) *m*/*z*: calcd. for C_15_H_12_N_3_OS [M + H]^+^ 282.0696, found 282.0682.

##### (*E*)-*N*’-(Pyridin-3-ylmethylene)benzo[*b*]thiophene-2-carbohydrazide (**I.d**)

White powder (129 mg, 67% yield). ^1^H NMR (300 MHz, DMSO-*d*_6_) δ 12.32 (s, 0.5H), 12.21 (s, 0.5H), 8.99 (s, 0.5H), 8.89 (s, 0.5H), 8.74–8.60 (m, 1H), 8.52 (s, 0.5H), 8.44 (s, 0.5H), 8.27 (s, 1H), 8.24–8.14 (m, 1H), 8.13–7.98 (m, 2H), 7.59–7.39 (m, 3H); ^13^C NMR (126 MHz, DMSO-*d*_6_) δ 161.86, 161.44, 158.42, 150.89, 150.69, 148.91, 145.43, 143.10, 142.06, 140.49, 140.39, 139.08, 138.09, 137.36, 137.10, 134.08, 133.62, 132.15, 130.14, 130.06, 126.87, 126.73, 126.11, 126.03, 125.69, 125.55, 125.20, 124.90, 124.11, 122.92, 122.67; HRMS (ESI) *m*/*z*: calcd. for C_15_H_12_N_3_OS [M + H]^+^ 282.0696, found 282.0694.

##### (*E*)-*N*’-(Pyridin-4-ylmethylene)benzo[*b*]thiophene-2-carbohydrazide (**I.e**)

Crystallized very slowly in the reaction mixture after the reflux. The mixture was concentrated and then engaged in recrystallization with MeOH and EtOAc. Light-yellow powder (92 mg, 30% yield). ^1^H NMR (300 MHz, DMSO-*d*_6_) δ 12.41 (s, 0.5H), 12.32 (s, 0.5H), 8.70 (s, 2H), 8.46 (s, 1H), 8.29 (s, 0.5H), 8.20–7.98 (m, 2.5H), 7.80 z(s, 1H), 7.71 (s, 1H), 7.58–7.43 (m, 2H); MS (ESI) *m*/*z* = 282.0 [M + H]^+^, 304.0 [M + Na]^+^.

##### (*E*)-*N*’-(3-Hydroxybenzylidene)benzo[*b*]thiophene-2-carbohydrazide (**I.f**)

Did not crystallize in the reaction mixture after the reflux. The mixture was concentrated and then engaged in recrystallization with MeOH and EtOAc (8:2). Light-brown powder (47 mg, 27% yield). ^1^H NMR (300 MHz, DMSO-*d*_6_) δ 12.10 (s, 0.5H), 11.99 (s, 0.5H), 9.75 (s, 0.5H), 9.67 (s, 0.5H), 8.41 (d, *J* = 11.2 Hz, 1H), 8.25 (s, 0.5H), 8.14–7.95 (m, 2.5H), 7.57–7.44 (m, 2H), 7.38–7.21 (m, 2.5H), 7.14 (d, *J* = 7.7 Hz, 0.5H), 6.88 (s, 1H); MS (ESI) *m*/*z* = 297.1 [M + H]^+^, 319.0 [M + Na]^+^.

##### (*E*)-*N*’-(4-Hydroxybenzylidene)benzo[*b*]thiophene-2-carbohydrazide (**I.g**)

White powder (32 mg, 10% yield). ^1^H NMR (300 MHz, DMSO-*d*_6_) δ 11.95 (s, 0.5H), 11.85 (s, 0.5H), 10.00 (s, 0.5H), 9.97 (s, 0.5H), 8.41 (s, 0.5H), 8.36 (s, 0.5H), 8.22 (s, 0.5H), 8.11–7.96 (m, 2.5H), 7.69 (d, *J* = 8.3 Hz, 1H), 7.59 (d, *J* = 8.3 Hz, 1H), 7.55–7.39 (m, 2H), 6.90 (d, *J* = 8.3 Hz, 1H), 6.85 (d, *J* = 8.3 Hz, 1H); MS (ESI) *m*/*z* = 297.1 [M + H]^+^, 319.0 [M + Na]^+^.

##### (*E*)-*N*’-(4-Hydroxy-3-methoxybenzylidene)benzo[*b*]thiophene-2-carbohydrazide (**I.h**)

White powder (141 mg, 41% yield). ^1^H NMR (300 MHz, DMSO-*d*_6_) δ 11.97 (s, 0.5H), 11.93 (s, 0.5H), 9.62 (s, 0.5H), 9.60 (s, 0.5H), 8.42 (s, 0.5H), 8.36 (s, 0.5H), 8.22 (s, 0.5H), 8.10–7.96 (m, 2.5H), 7.58–7.41 (m, 2.5H), 7.33 (s, 0.5H), 7.20 (d, *J* = 8.2 Hz, 0.5H), 7.13 (d, *J* = 8.2 Hz, 0.5H), 6.87 (t, *J* = 7.2 Hz, 1H), 3.92 (s, 1.5H), 3.84 (s, 1.5H); MS (ESI) *m*/*z* = 327.1 [M + H]^+^, 349.0 [M + Na]^+^.

##### (*E*)-*N*’-(4-Chlorobenzylidene)benzo[*b*]thiophene-2-carbohydrazide (**I.i**)

White solid (216 mg, 62% yield). ^1^H NMR (300 MHz, DMSO-*d*_6_) δ 12.22 (s, 0.5H), 12.10 (s, 0.5H), 8.44 (d, *J* = 11.4 Hz, 1H), 8.26 (s, 0.5H), 8.16 (s, 0.5H), 8.10–7.97 (m, 2H), 7.88 (d, *J* = 8.0 Hz, 1H), 7.79 (d, *J* = 8.0 Hz, 1H), 7.63–7.39 (m, 4H); MS (ESI) *m*/*z* = 315.0 [M + H]^+^, 337.0 [M + Na]^+^.

##### (*E*)-*N*’-(4-Fluorobenzylidene)benzo[*b*]thiophene-2-carbohydrazide (**I.j**)

White solid (173 mg, 44% yield). ^1^H NMR (300 MHz, DMSO-*d*_6_) δ 12.17 (s, 0.5H), 12.06 (s, 0.5H), 8.45 (d, *J* = 14.0 Hz, 1H), 8.25 (s, 0.5H), 8.17 (s, 0.5H), 8.13–7.99 (m, 2H), 7.92 (t, *J* = 6.8 Hz, 1H), 7.83 (t, *J* = 6.8 Hz, 1H), 7.56–7.42 (m, 2H), 7.40–7.27 (m, 2H); MS (ESI) *m*/*z* = 299.1 [M + H]^+^, 321.0 [M + Na]^+^.

##### (*E*)-*N*’-(3-Nitrobenzylidene)benzo[*b*]thiophene-2-carbohydrazide (**I.k**)

Yellowish solid (111 mg, 36% yield). ^1^H NMR (300 MHz, DMSO-*d*_6_) δ 12.41 (s, 0.5H), 12.30 (s, 0.5H), 8.68 (s, 0.5H), 8.58 (s, 1H), 8.44 (s, 0.5H), 8.36–8.16 (m, 3H), 8.05 (s, 2H), 7.87–7.68 (m, 1H), 7.58–7.41 (m, 2H); MS (ESI) *m*/*z* = 326.1 [M + H]^+^, 348.0 [M + Na]^+^.

##### (*E*)-*N*’-(4-Nitrobenzylidene)benzo[*b*]thiophene-2-carbohydrazide (**I.l**)

Yellowish solid (110 mg, 30% yield). ^1^H NMR (300 MHz, DMSO-*d*_6_) δ 12.43 (s, 0.5H), 12.35 (s, 0.5H), 8.56 (s, 0.5H), 8.44 (s, 0.5H), 8.33 (d, *J* = 8.4 Hz, 3H), 8.18–7.94 (m, 4H), 7.59–7.39 (m, 2H); ^13^C NMR (126 MHz, DMSO-*d*_6_) δ 161.98, 158.47, 147.90, 145.52, 142.41, 140.44, 138.97, 137.85, 137.34, 132.22, 128.35, 128.14, 126.25, 125.58, 125.18, 124.91, 124.12, 122.89, 122.71; HRMS (ESI) *m*/*z*: calcd. for C_16_H_10_N_3_O_3_S [M − H]^−^ 324.0448, found 324.0449.

##### (*E*)-2-((2-(Benzo[*b*]thiophene-2-carbonyl)hydrazono)methyl)benzoic acid (**I.m**)

White solid (88 mg, 45% yield). ^1^H NMR (300 MHz, DMSO-*d*_6_) δ 13.40 (s, 1H), 12.37 (s, 0.5H), 12.18 (s, 0.5H), 9.23 (s, 0.5H), 8.94 (s, 0.5H), 8.42 (s, 0.5H), 8.32 (s, 0.5H), 8.28–7.97 (m, 3H), 7.93 (dd, *J* = 7.7, 1.4 Hz, 1H), 7.81–7.61 (m, 1H), 7.61–7.42 (m, 3H); MS (ESI) *m*/*z* = 325.1 [M + H]^+^, 347.0 [M + Na]^+^.

##### (*E*)-3-((2-(Benzo[*b*]thiophene-2-carbonyl)hydrazineylidene)methyl)benzoic acid (**I.n**)

White powder (188 mg, 85% yield). ^1^H NMR (300 MHz, DMSO-*d*_6_) δ 12.29 (s, 0.5H), 12.16 (s, 0.5H), 8.52 (s, 0.5H), 8.49–8.30 (m, 1.5H), 8.32–7.91 (m, 5H), 7.71–7.57 (m, 1H), 7.57–7.41 (m, 2H); ^13^C NMR (101 MHz, DMSO-*d*_6_) δ 166.97, 161.82, 161.41, 158.41, 147.15, 143.87, 143.00, 140.46, 139.08, 138.17, 137.40, 134.68, 132.01, 131.65, 131.51, 131.11, 130.78, 129.36, 128.54, 127.54, 126.91, 126.72, 125.97, 125.56, 125.20, 124.96, 122.93, 122.49; HRMS (ESI) *m*/*z*: calcd. for C_17_H_13_N_2_O_3_S [M + H]^+^ 325.0641, found 325.0626.

##### (*E*)-4-((2-(Benzo[*b*]thiophene-2-carbonyl)hydrazono)methyl)benzoic acid (**I.o**)

White solid (92 mg, 39% yield). ^1^H NMR (300 MHz, DMSO-*d*_6_) δ 13.14 (s, 1H), 12.30 (s, 0.5H), 12.19 (s, 0.5H), 8.53 (s, 0.5H), 8.44 (s, 0.5H), 8.28 (s, 0.5H), 8.23 (s, 0.5H), 8.13–7.81 (m, 6H), 7.57–7.40 (m, 2H); MS (ESI) *m*/*z* = 325.1 [M + H]^+^, 347.0 [M + Na]^+^.

##### (*E*)-*N*’-(Benzo[*d*][1,3]dioxol-5-ylmethylene)-6-chlorobenzo[*b*]thiophene-2-carbohydrazide (**II.a**)

Intermediate **2b** was deprotected under the reported acidic conditions (TFA) to afford a white solid, which was engaged without further purification for the next step. White powder (75 mg, 31% yield). ^1^H NMR (300 MHz, DMSO-*d*_6_) δ 12.08 (s, 0.5H), 11.97 (s, 0.5H), 8.39 (d, *J* = 8.3 Hz, 1H), 8.33–7.97 (m, 3H), 7.48 (d, *J* = 9.3 Hz, 1.5H), 7.31 (s, 0.5H), 7.23 (t, *J* = 9.9 Hz, 1H), 7.10–6.97 (m, 1H), 6.13 (s, 1H), 6.11 (s, 1H); ^13^C NMR (101 MHz, DMSO-*d*_6_) δ 161.18, 157.85, 149.26, 148.16, 144.90, 144.40, 141.58, 139.44, 137.86, 136.02, 134.19, 131.37, 128.52, 128.28, 127.01, 126.83, 125.77, 125.48, 125.07, 123.96, 123.67, 122.48, 122.24, 108.65, 105.64, 105.27, 101.68; HRMS (ESI) *m*/*z*: calcd. for C_17_H_12_ClN_2_O_3_S [M + H]^+^ 359.0252, found 359.0254.

##### (*E*)-6-Chloro-*N*’-(pyridin-2-ylmethylene)benzo[*b*]thiophene-2-carbohydrazide (**II.b**)

Intermediate **2b** was deprotected under the reported acidic conditions (TFA) to afford, after an aqueous workup with NaHCO_3_ and extractions with EtOAc, a white solid, which was engaged without further purification for the next step. White solid (52 mg, 30% yield). ^1^H NMR (300 MHz, DMSO-*d*_6_) δ 12.08 (s, 0.5H), 11.97 (s, 0.5H), 8.39 (d, *J* = 8.3 Hz, 1H), 8.33–7.97 (m, 3H), 7.48 (d, *J* = 9.3 Hz, 1.5H), 7.31 (s, 0.5H), 7.23 (t, *J* = 9.9 Hz, 1H), 7.10–6.97 (m, 1H), 6.13 (s, 1H), 6.11 (s, 1H); ^13^C NMR (126 MHz, DMSO-*d*_6_) δ 161.52, 158.24, 153.04, 152.82, 149.69, 148.52, 145.41, 144.33, 141.70, 138.90, 137.81, 137.07, 136.13, 133.96, 131.69, 127.20, 126.99, 125.88, 125.68, 125.58, 124.63, 122.54, 122.24, 120.51, 120.16; HRMS (ESI) *m*/*z*: calcd. for C_15_H_11_ClN_3_OS [M + H]^+^ 316.0306, found 316.0315

##### (*E*)-*N*’-(Benzo[*d*][1,3]dioxol-5-ylmethylene)-6-fluorobenzo[*b*]thiophene-2-carbohydrazide (**II.c**)

Intermediate **2c** was deprotected under the reported acidic conditions (TFA) to afford a white solid, which was engaged without further purification for the next step. White powder (44 mg, 40% yield). ^1^H NMR (300 MHz, DMSO-*d*_6_) δ 12.05 (s, 0.5H), 11.93 (s, 0.5H), 8.38 (d, *J* = 8.7 Hz, 1H), 8.21 (s, 0.5H), 8.16–7.89 (m, 2.5H), 7.46 (s, 0.5H), 7.41–7.28 (m, 1.5H), 7.23 (t, *J* = 8.9 Hz, 1H), 7.11–6.92 (m, 1H), 6.13 (s, 1H), 6.11 (s, 1H); ^13^C NMR (101 MHz, DMSO-*d*_6_) δ 157.89, 149.33, 149.24, 148.14, 148.08, 148.06, 148.01, 144.80, 138.58, 138.55, 135.97, 134.17, 133.40, 131.45, 128.56, 128.31, 127.48, 125.09, 123.92, 123.62, 114.20, 109.15, 108.73, 108.63, 108.56, 108.49, 105.57, 105.26, 101.67; HRMS (ESI) *m*/*z*: calcd. for C_17_H_12_FN_2_O_3_S [M + H]^+^ 343.0547, found 343.0550.

##### (*E*)-6-Fluoro-*N*’-(pyridin-2-ylmethylene)benzo[*b*]thiophene-2-carbohydrazide (**II.d**)

Intermediate **2c** was deprotected under the reported acidic conditions (TFA) to afford, after an aqueous workup with NaHCO_3_ and extractions with EtOAc, a white solid, which was engaged without further purification for the next step. White solid (23 mg, 24% yield). ^1^H NMR (300 MHz, DMSO-*d*_6_) δ 12.31 (s, 1H), 8.64 (s, 0.5H), 8.54–7.71 (m, 6.5H), 7.59–7.14 (m, 2H); ^13^C NMR (126 MHz, DMSO-*d*_6_) δ 162.02, 160.08, 158.80, 150.13, 145.75, 144,71, 144.62, 141.43, 141.34, 140.47, 137.50, 137.04, 136.76, 136.33, 135.04, 132.22, 129.70, 127.83, 127.28, 127.20, 127.15, 127.07, 126.16, 125.03, 123.37, 120.85, 120.60, 118.72, 114.78, 114.43, 114.24, 114.01, 113.82, 109.37, 109.16, 108.79, 108.59; HRMS (ESI) *m*/*z*: calcd. for C_15_H_11_FN_3_OS [M + H]^+^ 300.0601, found 300.0603.

##### (*E*)-*N*’-((1H-Imidazol-4-yl)methylene)-6-chlorobenzo[*b*]thiophene-2-carbohydrazide (**III.a**)

Intermediate **2b** was deprotected under the reported acidic conditions (TFA) to afford, after an aqueous workup with NaHCO_3_ and extractions with EtOAc, a white solid, which was engaged without further purification for the next step. Beige powder (90 mg, 48% yield). ^1^H NMR (300 MHz, DMSO-*d*_6_) δ 12.92 (s, 0.5H), 12.52 (s, 0.5H), 11.92 (s, 0.5H), 11.85 (s, 0.5H), 8.53–8.35 (m, 1H), 8.31–8.13 (m, 1.5H), 8.13–7.96 (m, 1.5H), 7.90–7.67 (m, 1.5H), 7.67–7.40 (m, 1.5H); ^13^C NMR (101 MHz, DMSO-*d*_6_) δ 157.66, 144.16, 141.51, 139.58, 137.86, 136.71, 131.38, 130.92, 126.77, 125.75, 125.34, 124.90, 122.48, 122.08; MS (ESI) *m*/*z* = 305.1 [M + H]^+^, 327.0 [M + Na]^+^.

##### (*E*)-6-Chloro-*N*’-(furan-2-ylmethylene)benzo[*b*]thiophene-2-carbohydrazide (**III.b**)

Intermediate **2b** was deprotected under the reported acidic conditions (TFA) to afford a white solid, which was engaged without further purification for the next step. Beige powder (145 mg, 78% yield). ^1^H NMR (300 MHz, DMSO-*d*_6_) δ 12.12 (s, 0.5H), 12.04 (s, 0.5H), 8.43 (s, 0.5H), 8.34 (s, 0.5H), 8.30–8.12 (m, 1.5H), 8.12–7.96 (m, 1.5H), 7.88 (s, 1H), 7.57–7.41 (m, 1H), 7.06 (s, 0.5H), 6.99 (s, 0.5H), 6.67 (s, 1H); ^13^C NMR (101 MHz, DMSO-*d*_6_) δ 158.34, 149.66, 145.94, 142.04, 139.68, 138.42, 138.25, 135.33, 134.70, 131.97, 131.65, 127.49, 127.30, 126.24, 125.90, 125.64, 122.96, 122.56, 114.70, 114.23, 112.79; MS (ESI) *m*/*z* = 305.0 [M + H]^+^, 327.0 [M + Na]^+^.

##### (*E*)-6-Chloro-*N*’-((5-(hydroxymethyl)furan-2-yl)methylene)benzo[*b*]thiophene-2-carbohydrazide (**III.c**)

Intermediate **2b** was deprotected under the reported acidic conditions (TFA) to afford a white solid, which was engaged without further purification for the next step. Beige powder (94 mg, 46% yield). ^1^H NMR (300 MHz, DMSO-*d*_6_) δ 12.09 (s, 0.5H), 12.03 (s, 0.5H), 8.44 (s, 0.5H), 8.36–8.15 (m, 2H), 8.12–7.94 (m, 1.5H), 7.50 (d, *J* = 8.4 Hz, 1H), 7.15–6.85 (m, 1H), 6.47 (s, 1H), 5.44 (t, *J* = 6.3 Hz, 1H), 4.63–4.29 (m, 2H); ^13^C NMR (101 MHz, DMSO-*d*_6_) δ 158.22, 157.89, 148.44, 141.61, 139.28, 137.89, 136.46, 135.18, 134.30, 131.54, 131.16, 127.07, 126.86, 125.82, 125.45, 125.19, 122.51, 122.11, 115.54, 114.25, 109.38, 55.82; MS (ESI) *m*/*z* = 335.0 [M + H]^+^, 357.0 [M + Na]^+^.

##### (*E*)-6-Chloro-*N*’-(pyridin-2-ylmethylene)benzofuran-2-carbohydrazide (**III.e**)

Intermediate **2e** was deprotected under the reported acidic conditions (TFA) to afford, after an aqueous workup with NaHCO_3_ and extractions with EtOAc, a white solid, which was engaged without further purification for the next step. White powder (63 mg, 44% yield). ^1^H NMR (300 MHz, DMSO-*d*_6_) δ 12.45 (s, 1H), 8.63 (dd, *J* = 4.8, 1.5 Hz, 1H), 8.54 (s, 1H), 8.03–7.73 (m, 5H), 7.49–7.41 (m, 2H); ^13^C NMR (101 MHz, DMSO-*d*_6_) δ 154.59, 153.05, 149.11, 148.57, 137.00, 131.93, 126.04, 124.67, 124.27, 120.17, 112.29, 111.20; HRMS (ESI) *m*/*z*: calcd. for C_15_H_11_ClN_3_O_2_ [M + H]^+^ 300.0534, found 300.0533.

##### (*E*)-*N*’-(Pyridin-2-ylmethylene)-6-(trifluoromethyl)benzo[*b*]thiophene-2-carbohydrazide (**III.f**)

Intermediate **2d** was deprotected under the reported acidic conditions (TFA) to afford, after an aqueous workup with NaHCO_3_ and extractions with EtOAc, a white solid, which was engaged without further purification for the next step. White powder (64 mg, 44% yield). ^1^H NMR (300 MHz, DMSO-*d*_6_) δ 12.42 (s, 1H), 8.70–8.59 (m, 2H), 8.52 (d, *J* = 13.4 Hz, 1H), 8.39–8.18 (m, 3H), 8.07–7.85 (m, 2H), 7.77 (d, *J* = 7.9 Hz, 1H), 7.46 (d, *J* = 6.5 Hz, 1H). ^13^C NMR (101 MHz, DMSO-*d*_6_) δ 161.37, 158.04, 152.99, 152.78, 149.68, 148.84, 145.64, 142.91, 141.86, 141.72, 140.29, 139.92, 137.13, 137.02, 136.72, 131.50, 126.66, 126.51, 125.55, 124.69, 124.61, 121.45, 121.09, 120.88, 120.83, 120.77, 120.71, 120.68, 120.59, 120.19; HRMS (ESI) *m*/*z*: calcd. for C_16_H_11_F_3_N_3_OS [M + H]^+^ 350.0569, found 350.0572.

#### 2.1.8. Procedure for *N*’-(Pyridin-2-ylmethyl)benzo[*b*]thiophene-2-carbohydrazide (**I.p**) 

Under H_2_ atmosphere, a solution of (*E*)-*N*’-(pyridin-2-ylmethylene)benzo[*b*]thiophene-2-carbohydrazide (0.12 mmol, 1 eq.) and palladium on carbon 10% (0.019 mmol, 15 mol%) in 5 mL of EtOAc and 1 mL of MeOH was stirred at room temperature for 7 h. The mixture was filtered on Celite^®^ and washed with EtOAc, and the residue was concentrated under vacuum and purified by chromatography (eluent: EtOAc/MeOH 98:2). (Adapted from Kim et al. [22]).

##### *N*’-(Pyridin-2-ylmethyl)benzo[*b*]thiophene-2-carbohydrazide (**I.p**)

White powder (30 mg, 60% yield). ^1^H NMR (300 MHz, DMSO-*d*_6_) δ 10.34 (d, *J* = 5.1 Hz, 1H), 8.63–8.40 (m, 1H), 8.04–7.98 (m, 2H), 7.95–7.88 (m, 1H), 7.78 (td, *J* = 7.7, 1.8 Hz, 1H), 7.54 (d, *J* = 7.7 Hz, 1H), 7.49–7.38 (m, 2H), 7.32–7.21 (m, 1H), 5.76 (q, *J* = 5.1 Hz, 1H), 4.12 (d, *J* = 5.1 Hz, 2H); ^13^C NMR (101 MHz, DMSO-*d*_6_) δ 161.02, 158.23, 148.90, 140.04, 139.12, 138.16, 136.59, 126.25, 125.20, 125.00, 124.69, 122.82, 122.36, 122.32, 56.21; HRMS (ESI) *m*/*z*: calcd. for C_15_H_14_N_3_OS [M + H]^+^ 284.0852, found 284.0858.

#### 2.1.9. Procedure for Pyridine 2-aldoxime (**6**)

To a solution of pyridine-2-carboxaldehyde (1.87 mmol, 1 eq.) and hydroxylamine hydrochloride (2.33 mmol, 1.25 eq.) in water (5 mL) was added dropwise a solution of sodium bicarbonate (2.33 mmol, 1.25 eq.) in water (10 mL), and the mixture was stirred for 3 h at room temperature. The solution was then extracted with EtOAc and the organic layer dried over Na_2_SO_4_ and concentrated under vacuum. (Adapted from Younus Wani et al. 2011 ([23]).

##### Pyridine 2-aldoxime (**6**)

White powder (186 mg, 81%). ^1^H NMR (300 MHz, Chloroform-*d*) δ 8.99 (s, 1H), 8.63 (ddd, *J* = 4.9, 1.8, 1.0 Hz, 1H), 8.31 (s, 1H), 7.82 (dt, *J* = 8.1, 1.0 Hz, 1H), 7.72 (td, *J* = 8.1, 1.8 Hz, 1H), 7.29 (ddd, *J* = 8.1, 4.9, 1.0 Hz, 1H). (Spectrum in accordance with Zhang et al. [24]).

#### 2.1.10. Procedure for (*E*)-Picolinaldehyde *O*-(6-chlorobenzo[*b*]thiophene-2-carbonyl) oxime (**III.d**)

Under a dried and inert atmosphere (N_2_), a solution of 6-chlorobenzothiophene-2-carboxylic acid (1.06 mmol, 1.3 eq.) and pyridine 2-aldoxime (0.82 mmol, 1.0 eq.) in anhydrous DCM (3 mL) was cooled to 0 °C. Then, DMAP (0.12 mmol, 0.15 eq.) and DCC (1.06 mmol, 1.3 eq.) were gradually added to the mixture. The mixture was stirred at room temperature overnight and then filtered on Celite^®^ and washed with DCM. The filtrate was then concentrated, and the residue was purified by column chromatography (eluent: pentane/EtOAc 2:1).

##### (*E*)-Picolinaldehyde *O*-(6-chlorobenzo[*b*]thiophene-2-carbonyl) oxime (**III.d**)

Orange powder (166 mg, 64% yield). ^1^H NMR (300 MHz, DMSO-*d*_6_) δ 8.85 (s, 1H), 8.77–8.73 (m, 1H), 8.43 (d, *J* = 0.7 Hz, 1H), 8.33 (dt, *J* = 1.9, 0.7 Hz, 1H), 8.10 (dd, *J* = 8.5, 0.7 Hz, 1H), 8.04–7.95 (m, 2H), 7.62–7.54 (m, 2H); ^13^C NMR (126 MHz, DMSO-*d*_6_) δ 159.04, 158.37, 150.12, 148.94, 142.67, 137.31, 137.04, 132.61, 131.55, 131.02, 127.37 (2C), 126.05, 122.57, 122.49; HRMS (ESI) *m*/*z*: calcd. for C_15_H_10_ClN_2_O_2_S [M + H]*^+^* 317.0146, found 317.0136.

### 2.2. Biological Assays

#### 2.2.1. Minimum Inhibitory Concentration (MIC) Evaluation

MICs were evaluated in CaMHB (cation-adjusted Mueller–Hinton broth) by the method of microdilution in liquid medium, which follows the CLSI recommendations (Clinical and Laboratory Standards Institute (CLSI), methods for dilution antimicrobial susceptibility tests for bacteria that grow aerobically; approved standard seventh edition. Clinical and Laboratory Standards Institute, Wayne, PA, USA). Evaluated compounds were diluted in DMSO with a concentration of 5 mg/mL and then further diluted in CaMHB. The 0.5 MacFarland bacterial suspensions were made from colonies previously grown on blood agar plate (COS, Biomérieux) in a saline solution (0.45% NaCl). They were diluted in CaMHB (1/100) before addition into 96-well microplates. MICs were carried out in triplicate and determined after 18 h of incubation at 37 °C. The median values were reported.

#### 2.2.2. Cytotoxicity Assay

Cytotoxicity was evaluated using a propidium iodide assay. Briefly, human lung adenocarcinomic A549 cells were plated on black flat 96-well plates (Greiner) at a concentration of 0.5 × 10^6^ cells/mL in DMEM GlutaMAX (Gibco) supplemented with 10% fetal bovine serum for 24 h in a humidified atmosphere at 37 °C and 5% CO_2_. The medium was then removed and fresh medium with 2.5 µg/mL of propidium iodide and tested compounds (128 µg/mL of **II.b** and 0.2 µg/mL for recombinant Hla) was added. Absorbance at 618 nm was measured every 10 min over 7 h. Each concentration was made in triplicate and mean values are displayed with standard deviation.

## 3. Results and Discussion

### 3.1. Synthesis and Biological Evaluation of Series **I**—Benzo[b]thiophene-2-Acylhydrazones

**Figure 1:**
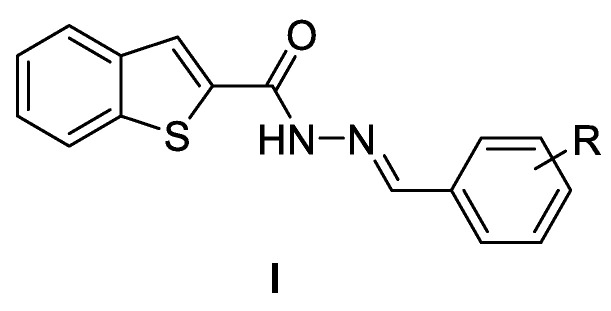
General structure of set I derivatives.

Benzo[*b*]thiophene-2-acylhydrazones **I** derived from various aldehydes were first investigated (Figure 1).

**Scheme 1:**
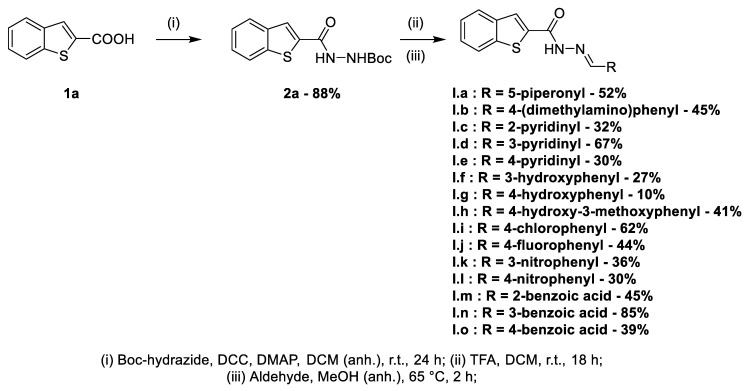
Synthesis of set **I** derivatives.

The synthetic route to these compounds was achieved by the conversion of benzothiophene-2-carboxylic acid **1a** into *tert*-butyl 2-(benzothiophene-2-carbonyl)hydrazinecarboxylate **2a** in 88% yield using DCC as a coupling reagent. This method was preferred to the substitution of methyl benzothiophene-2-carboxylate with hydrazine, leading to a mixture of products difficult to purify [25]. Then, the *tert*-butyl carboxylate group was removed under acidic conditions using TFA to give a crude material which was then engaged without purification for reaction with diversely substituted aromatic or heteroaromatic aldehydes under acidic and refluxing conditions to form the imine bond of the acylhydrazone (Scheme 1).

All the synthesized derivatives **I** were conveniently obtained in satisfactory yields by spontaneous precipitation in pure form in the reaction medium after two hours of reflux. Compounds such as the 2-pyridinyl derivative **I.c** required the careful removal of all traces of acid after TFA deprotection, by a basic aqueous work-up and extraction with ethyl acetate before reaction with 2-pyridinecarboxaldehyde.

**Figure 2:**
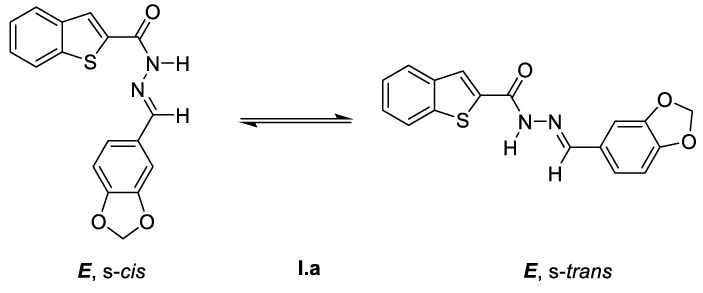

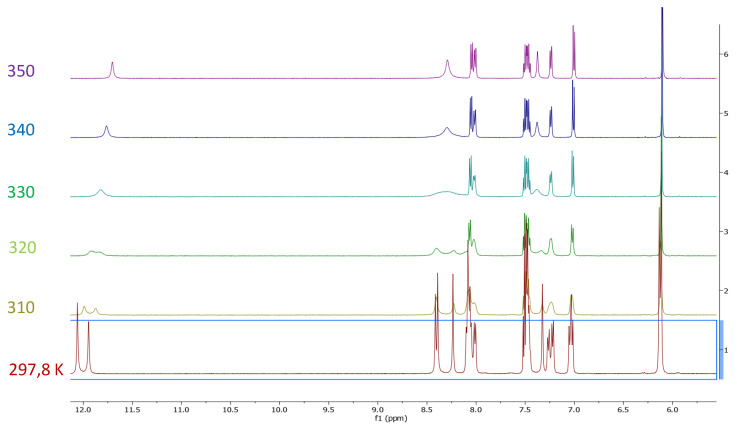
^1^H NMR spectra of **I.a** at different temperatures.

Structural analysis showed the presence of mixtures of geometric isomers for each final compound, as ^1^H and ^13^C NMR signals were split in two (spectra available in Supplementary Materials, Figure S1). Theoretically, each *N*-acylhydrazone derivative could present four different isomers due to (*E*)/(*Z*) and s-*cis*/s-*trans* isomerism. Previous studies have shown that only the more stable (*E*)-isomer is generated due to the steric hindrance and under such thermodynamic conditions, and that s-*cis*- and s-*trans*-isomers (rotamers) were identified by ^1^H NMR spectroscopy [13,26,27]. To demonstrate the presence of rotamers in the case of the piperonyl derivative **I.a**, the temperature of ^1^H NMR experiments was gradually increased, and the expected coalescence phenomenon was observed at 330 K and at higher temperatures (Figure 2).

This first set of compounds was then tested on three strains of *Staphylococcus aureus*, including a reference (ATCC29213) and two clinically isolated strains, resistant to methicillin (SF8300) and daptomycin (ST20171643), respectively. The purpose of this experiment was to establish the minimum inhibitory concentration (MIC), which is the minimum concentration of compound needed to prevent the growth of a standardized bacterial inoculum, for the benzo[*b*]thiophene-derived acylhydrazones. The results are presented in Table 1.

In this series, the two most active compounds are the piperonyl derivative **I.a,** which shows a MIC of 32 µg/mL for ATCC29213 and a range from 16 to 64 µg/mL for the two strains of antibiotic-resistant *S. aureus*, and the pyridinyl compound **I.c**, which shows a MIC of 16 µg/mL for every strain tested, making it the most potent molecule in this series. Interestingly, the position of the nitrogen atom in position 2 of the pyridine moiety in **I.c** was found to be important, as the other pyridinyl compounds **I.d** and **I.e**, in which the nitrogen is, respectively, in position 3 and 4, were found to be inactive. In this set, another position of interest in the phenyl ring is position 4 (**I.i**, **I.l** and, to a lesser extent, **I.a**), with interesting MICs for the products, while the others had no biological activity.

**Scheme 2:**
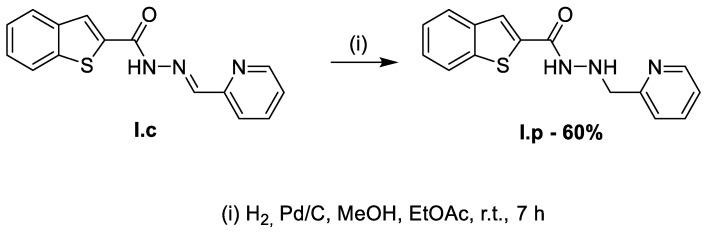
Synthesis of compound **I.p**.

To examine the effect of the reduction of the imine bond and its consequences on free rotation and conjugation, the pyridinyl compound **I.c** was reduced using palladium over carbon under a H_2_ atmosphere with a 60% yield (Scheme 2). Reduced compound **I.p** exhibited a MIC four times higher than the one of **I.c** (Table 1). It is therefore suggested that preventing the free rotation of the pyridine ring and allowing conjugation due to the tautomeric form of the amide bond is important for the activity of **I.c**. An effect of hydrophobicity is also observed, decreasing the LogP from 3.13 for **I.c** to 2.33 for **I.p**.

### 3.2. Synthesis and Biological Evaluation of Series **II**—6-Halogenobenzo[b]thiophene-2-Acylhydrazones

**Figure 3:**
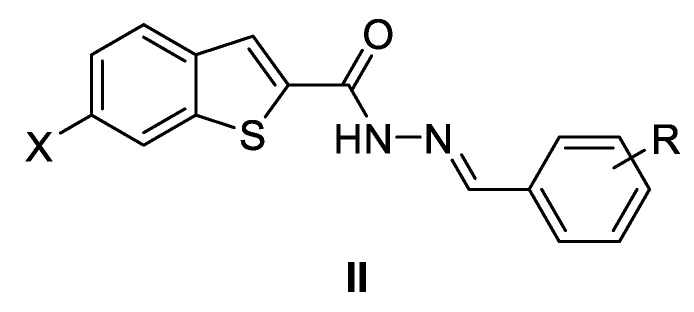
General structure of set **II** derivatives.

Based on this first set of results, we selected the two compounds **I.a** and **I.c** (bearing a 5-piperonyl and a 2-pyridinyl moiety, respectively) as hits for further structural modulation. With 6-halogenobenzo[*b*]thiophene rings, being easily accessible using 2-fluoro-4-halogenobenzaldehyde as precursor, we prepared a series of four compounds, namely two piperonyl or pyridinyl compounds bearing a fluorine or chlorine atom, respectively (Figure 3).

**Scheme 3:**
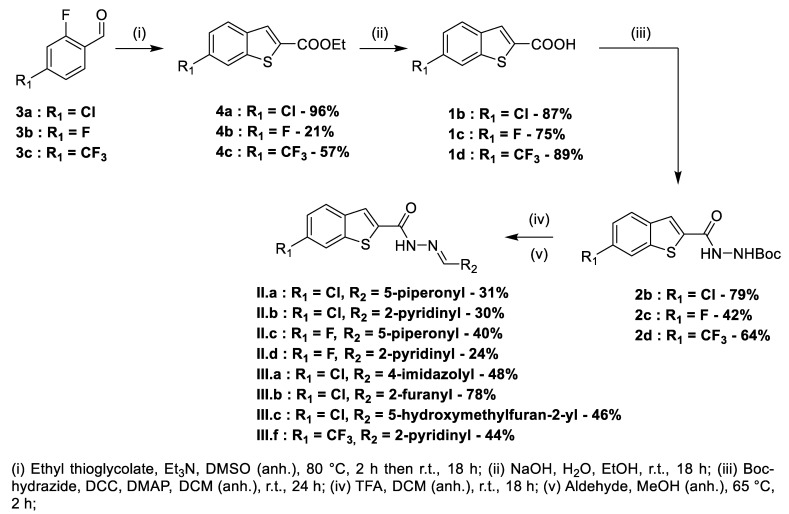
Synthesis of set **II** and **III** derivatives.

In order to synthesize the acylhydrazones, 6-chloro- and 6-fluorobenzo[*b*]thiophene-2-carboxylic acids **1b** and **1c** were thus prepared. First, 2-fluoro-4-halogenobenzaldehyde reacted with ethyl thioglycolate by nucleophilic substitution followed by an intramolecular cyclization to obtain compounds **4a** and **4b**, as described in the literature [14]. These esters were then saponified with sodium hydroxide to give the desired carboxylic acids **1b** and **1c**. The general procedure used for the first set was applied to the latter to obtain compounds **II.a**–**d** with similar yields, as shown in Scheme 3.

These compounds were also tested to establish their MIC against *S. aureus* with the same method used for set **I**, and the results are presented in Table 2.

This short series allowed us to identify the chloropyridinyl compound **II.b** as significantly more active compared with its non-halogenated counterpart **I.c,** reaching 4 µg/mL for the three strains. The presence of a fluorine atom in position 6 of the benzothiophene for the pyridinyl compound did not change the activity. Both halogenated compounds derived from the piperonyl compound **I.a** were deprived of biological activity (**II.a** and **II.c**).

### 3.3. Synthesis and Biological Evaluation of Series **III**—Structural Modifications of **II.b**

Attempts to improve the activity of the chloropyridinyl compound **II.b** were then undertaken by replacing the pyridine ring with three five-bond rings, namely imidazole, furan and 5-hydroxymethylfuran. The procedure used was the same as the one used for chlorinated derivatives **II.a** and **II.b** (Scheme 3)**.** As shown in Table 3, the activity remains better for compound **II.b**, confirming the importance of the 2-pyridinyl group.

**Scheme 4:**
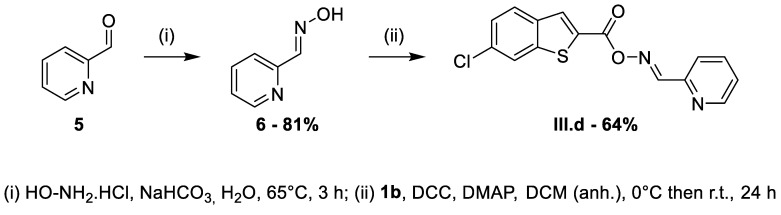
Synthesis of **III.d**.

Then, the acylhydrazone was replaced by an acyloxime moiety, leading to a more flexible molecule and losing the s-*cis*/s-*trans* isomerism. Compound **III.d** was obtained through the conversion of pyridine-2-carboxaldehyde into the corresponding oxime **6**, followed by the acylation from 6-chlorobenzo[*b*]thiophene-2-carboxylic acid **1b** (Scheme 4).

Compound **III.d** showed no anti-staphylococcal activity at concentrations lower or equal to 256 µg/mL (Table 3). Therefore, the rigidity imposed by the acylhydrazone moiety is critical to the biological activity.

**Scheme 5:**
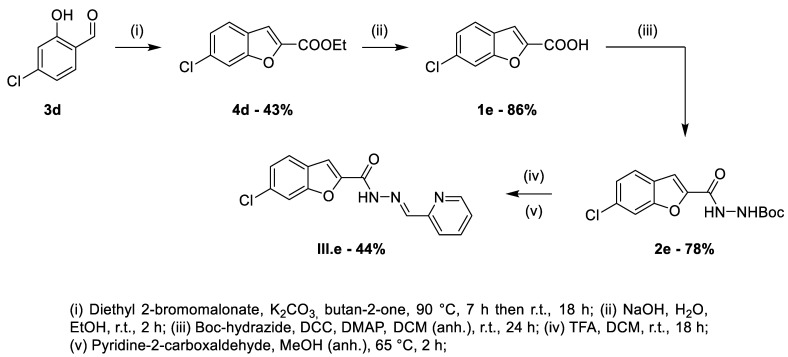
Synthesis of **III.e**.

Then, the effect of the hydrophobicity of the compounds on the antibacterial activity was investigated using the benzo[*b*]furan counterpart of **II.b** (Scheme 5). First, 4-chloro-2-hydroxybenzaldehyde **3d** was converted into the ester **4d** using diethyl 2-bromomalonate as described [18]. Then, the synthetic route was the same as described before for benzo[*b*]thiophene acylhydrazone derivatives.

As shown in Table 3, compound **III.e** showed a weak activity against *S. aureus* with MIC values ≥ 128 µg/mL. Therefore, the hydrophobicity of the benzo[*b*]thiophene ring is critical for the biological activity. As expected, the replacement of the sulfur by an oxygen atom decreases the overall LogP of the compound (3.79 for **II.b** and 3.15 for **III.e**).

Finally, the chlorine atom was replaced by a bioisostere, a trifluoromethyl group, using the same synthetic strategy (Scheme 3), leading to compound **III.f**. Even if the MICs are interesting (Table 3), the chlorinated derivative **II.b** remains the most active compound.

### 3.4. Cytotoxicity Assay of **II.b**

**Figure 4:**
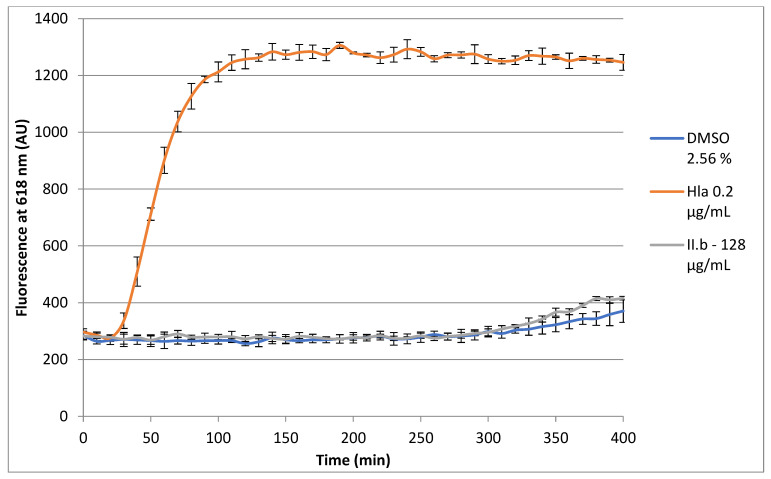
Effect of **II.b** on the growth of A549 cells.

Due to its interesting antistaphylococcal activity, the potential mammalian cytotoxicity of the chloropyridinyl derivative **II.b** was examined. Therefore, a 7 h propidium iodide (PI)-based assay was used on adenocarcinomic human alveolar basal epithelial cells (A549), with recombinant *S. aureus* α-hemolysin as a positive control. As depicted in Figure 4, no cytotoxicity was found for **II.b** after 7 h at a concentration of 128 µg/mL (32 times the MIC).

## 4. Conclusions

In this study, benzo[*b*]thiophene-based acylhydrazones were shown to be interesting compounds acting as anti-staphylococcal agents. More precisely, (*E*)-6-chloro-*N*’-(pyridin-2-ylmethylene)benzo[*b*]thiophene-2-carbohydrazide **II.b** showed an equal minimum inhibitory concentration of 4 µg/mL for the three strains of *S. aureus*, including two clinically isolated strains of drug-resistant *S. aureus*. Moreover, **II.b** showing no cytotoxicity on A549 cells; this suggests that the chloropyridinyl benzothiophene acylhydrazone structure is pertinent for future work on chemical diversification and the understanding of the mode of action.

## Figures and Tables

**Table 1:** Minimum inhibitory concentrations of set **I** molecules against *S. aureus* strains.

Name	Structure	LogP	MIC (µg/mL)		
			ATCC 29213 ^a^	SF8300 ^b^	ST20171643 ^c^
**I.a**	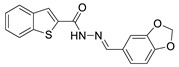	4.20	32	64	16
**I.b**	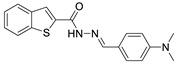	4.41	>256	>256	>256
**I.c**	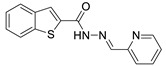	3.13	16	16	16
**I.d**	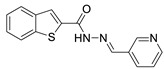	3.07	>256	>256	>256
**I.e**	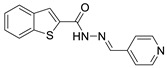	3.02	>256	>256	>256
**I.f**	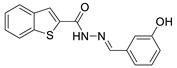	3.80	>256	>256	ND
**I.g**	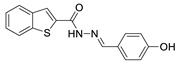	3.83	>256	>256	>256
**I.h**	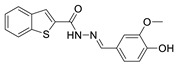	3.64	>256	>256	>256
**I.i**	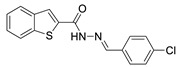	4.98	128	128	128
**I.j**	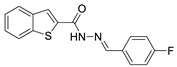	4.47	>256	>256	128
**I.k**	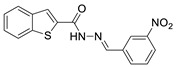	4.24	>256	>256	ND
**I.l**	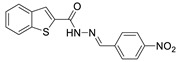	4.26	64	64	32
**I.m**	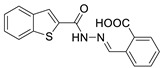	3.83	256	>256	>256
**I.n**	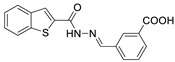	4.19	>256	>256	256
**I.o**	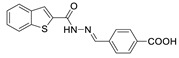	4.22	>256	>256	256
**I.p**	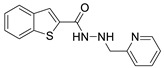	2.33	64	64	64

^a^: methicillin-sensitive *S. aureus*; ^b^: methicillin-resistant *S. aureus*; ^c^: daptomycin-resistant *S. aureus.*

**Table 2:** Minimum inhibitory concentrations of set **II** molecules against *S. aureus* strains.

Name	Structure	LogP	MIC (µg/mL)		
			ATCC 29213 ^a^	SF8300 ^b^	ST20171643 ^c^
**II.a**	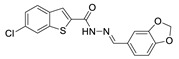	4.85	>256	>256	>256
**II.b**	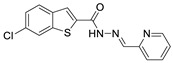	3.79	4	4	4
**II.c**	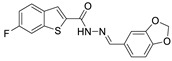	4.33	>256	>256	>256
**II.d**	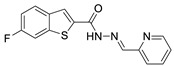	3.27	32	16	16

^a^: methicillin-sensitive *S. aureus*; ^b^: methicillin-resistant *S. aureus*; ^c^: daptomycin-resistant *S. aureus.*

**Table 3:** Minimum inhibitory concentrations of set **III** molecules against *S. aureus* strains.

Name	Structure	LogP	MIC (µg/mL)		
			ATCC 29213 ^a^	SF8300 ^b^	ST20171643 ^c^
**III.a**	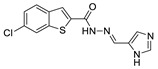	3.19	256	64	64
**III.b**	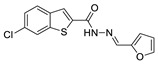	4.22	>256	256	256
**III.c**	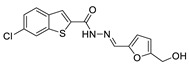	3.79	>256	256	256
**III.d**	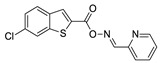	3.89	>256	>256	>256
**III.e**	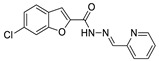	3.15	>256	256	128
**III.f**	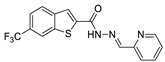	4.01	32	8	16

^a^: methicillin-sensitive *S. aureus*; ^b^: methicillin-resistant *S. aureus*; ^c^: daptomycin-resistant *S. aureus.*

## Data Availability

Data can be found in the manuscript and in the Supplementary Materials.

## Supplementary Materials

The following are available online at https://www.mdpi.com/article/10.3390/biom12010131/s1, Figure S1: ^1^H- and ^13^C-NMR spectra for new compounds.
