# Supplementary material for: Synthesis and Biological Evaluation of Benzo[b]thiophene Acylhydrazones as Antimicrobial Agents against Multidrug-Resistant Staphylococcus aureus
Source: Biomolecules. 2022 Jan 14;12(1):131. doi: 10.3390/biom12010131 (PMC8773820; doi:10.3390/biom12010131)
Supplement: Supplementary file 1 [file biomolecules-12-00131-s001.zip › biomolecules-1531396-supplementary.pdf]

Supplementary Materials

Figure S1. ^1H and ^{13}C NMR spectra for compounds **1b**, **2a**, **2b**, **2c**, **2d**, **2e**, **I.a**, **I.b**, **I.c**, **I.d**, **I.e**, **I.f**, **I.g**, **I.h**, **I.i**, **I.j**, **I.k**, **I.l**, **I.m**, **I.n**, **I.o**, **I.p**, **II.a**, **II.b**, **II.c**, **II.d**, **III.a**, **III.b**, **III.c**, **III.d**, **III.e** and **III.f.**

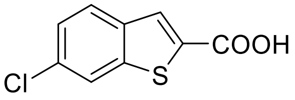
COOH
Cl
S

1b

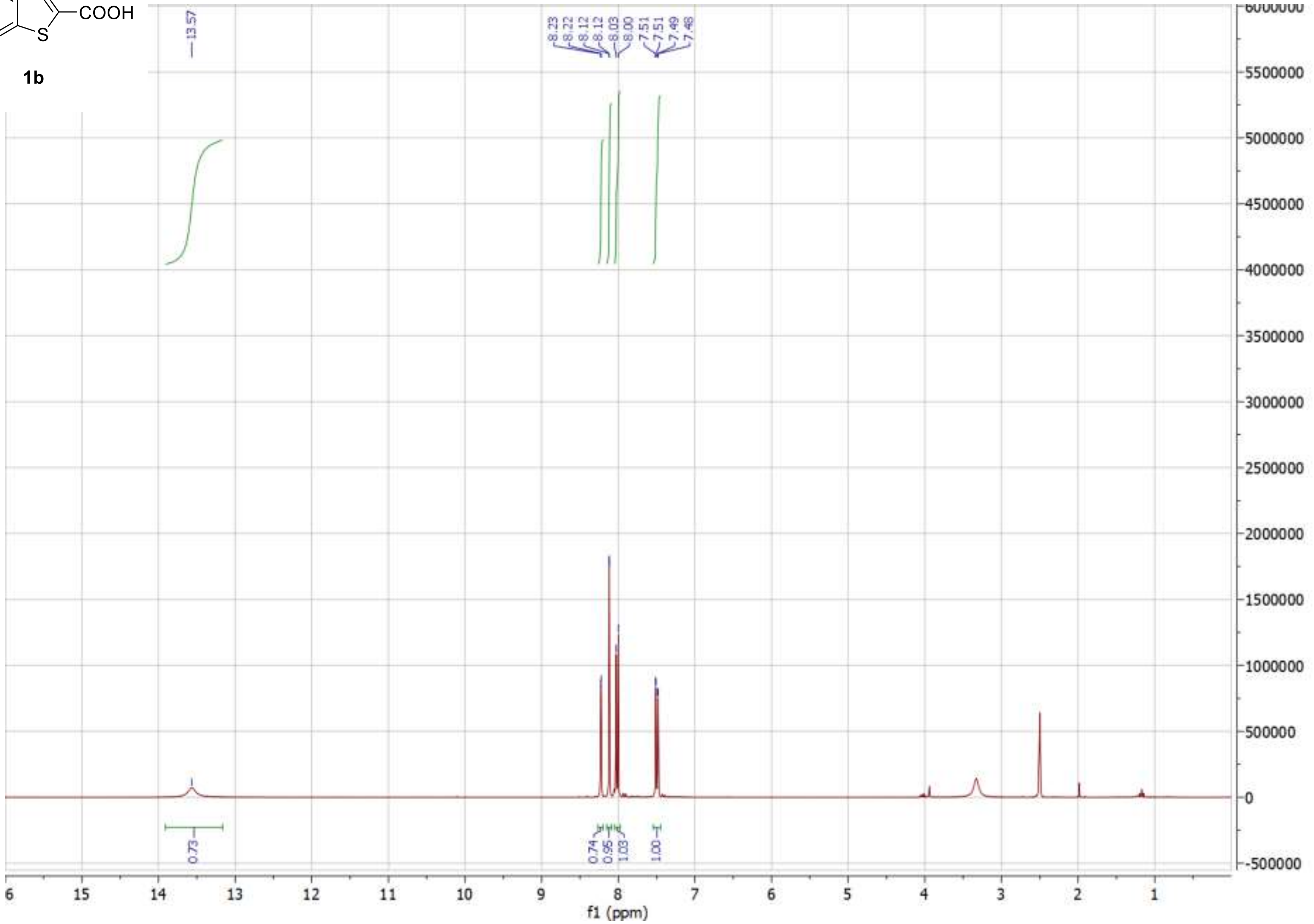
13.57
8.23
8.22
8.12
8.12
8.03
8.00
7.51
7.51
7.49
7.48
0.73
0.74
0.95
1.03
1.00
6000000
5500000
5000000
4500000
4000000
3500000
3000000
2500000
2000000
1500000
1000000
500000
0
-500000
6 15 14 13 12 11 10 9 8 7 6 5 4 3 2 1
f1 (ppm)

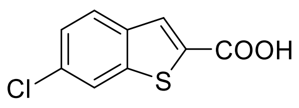

COOH
Cl
S

1b

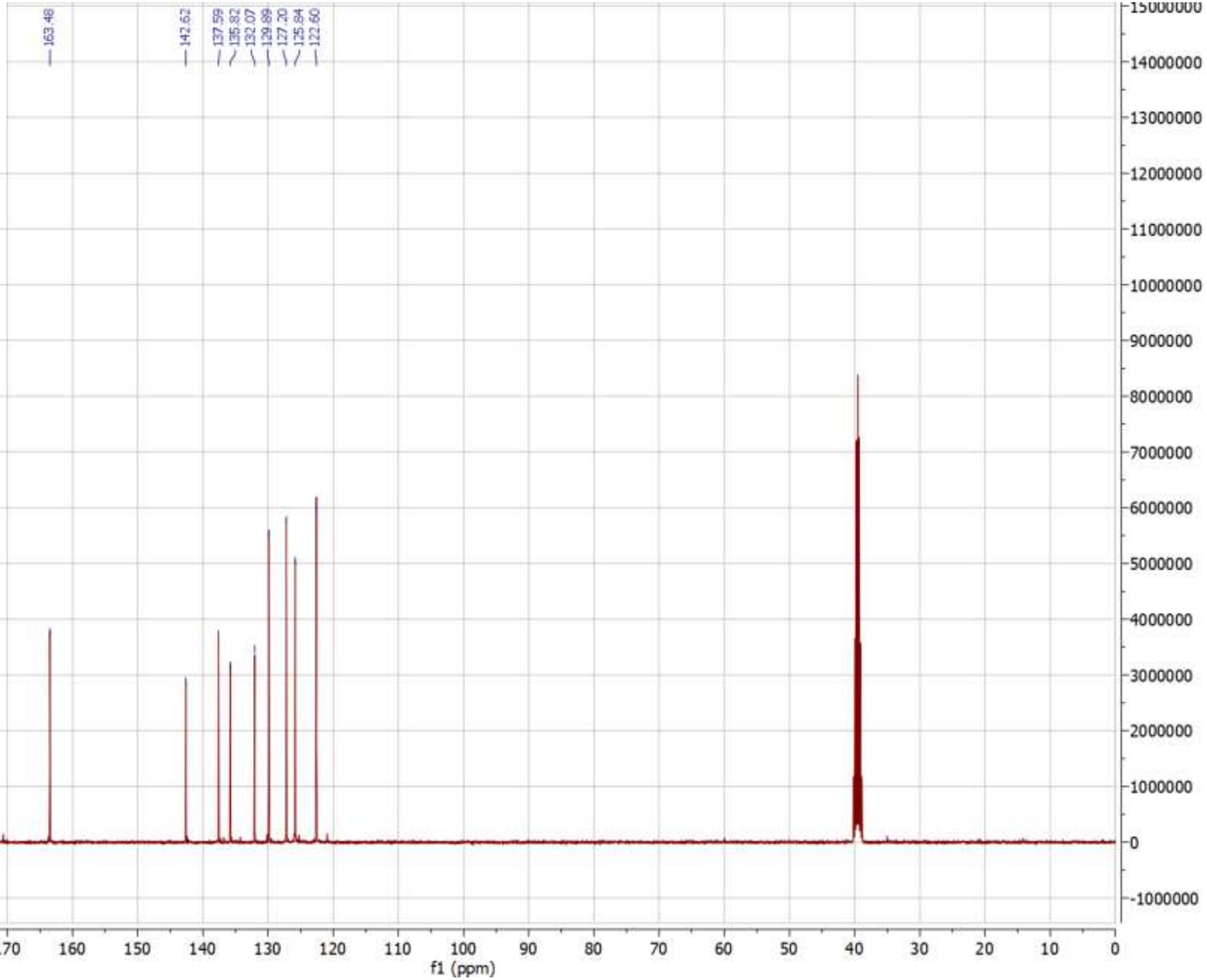

163.48
142.62
137.59
135.82
132.07
129.89
127.20
125.84
122.60
15000000
14000000
13000000
12000000
11000000
10000000
9000000
8000000
7000000
6000000
5000000
4000000
3000000
2000000
1000000
0
-1000000
190
180
170
160
150
140
130
120
110
100
90
80
70
60
50
40
30
20
10
0
f1 (ppm)

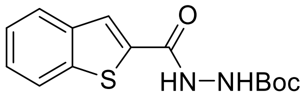

2a

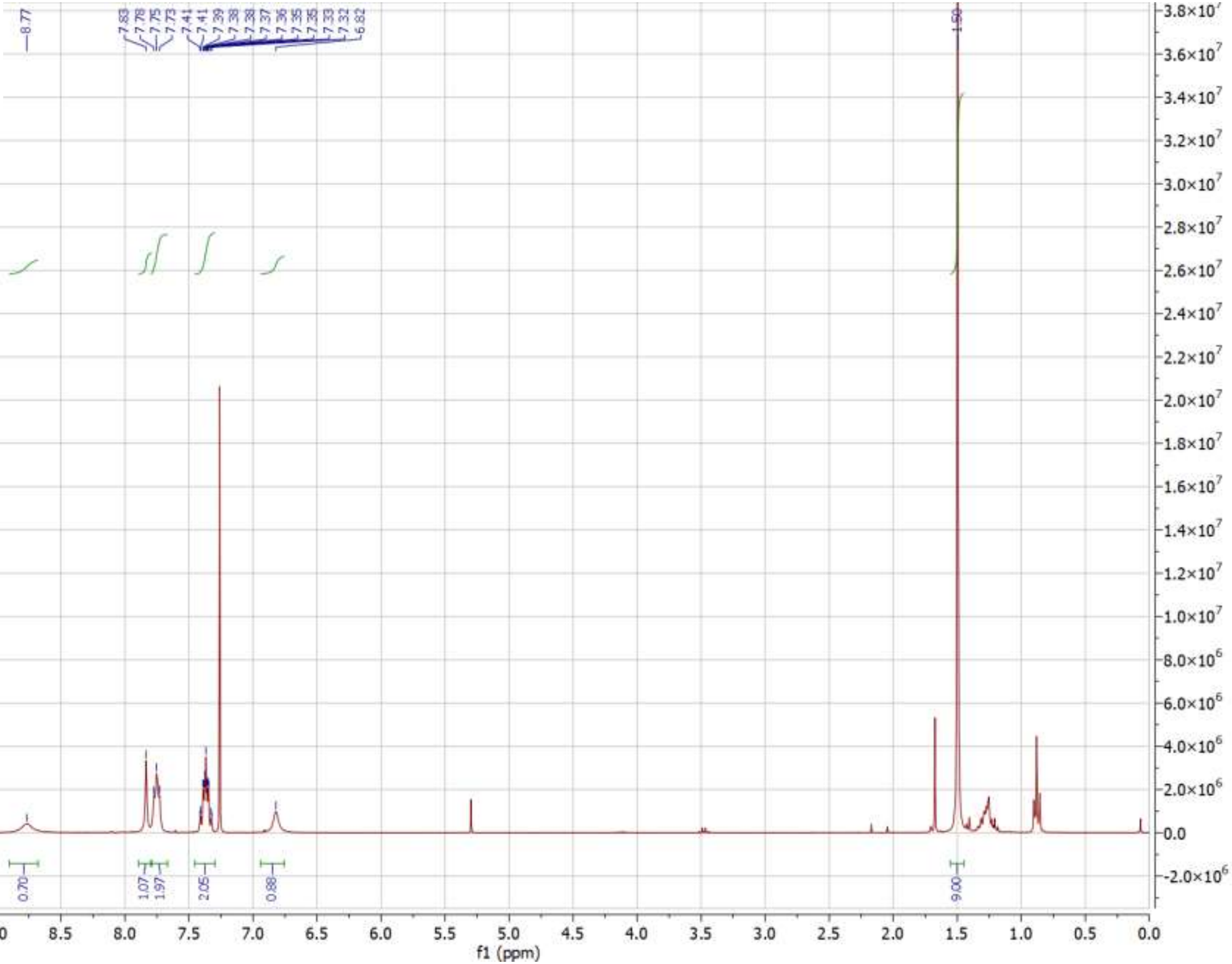

8.77
7.83
7.78
7.75
7.73
7.41
7.41
7.39
7.38
7.38
7.37
7.36
7.35
7.35
7.33
7.32
6.82
1.50
0.70
1.07
1.97
2.05
0.88
9.00
f1 (ppm)

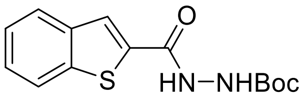
O
S
HN−NHBoc

2a

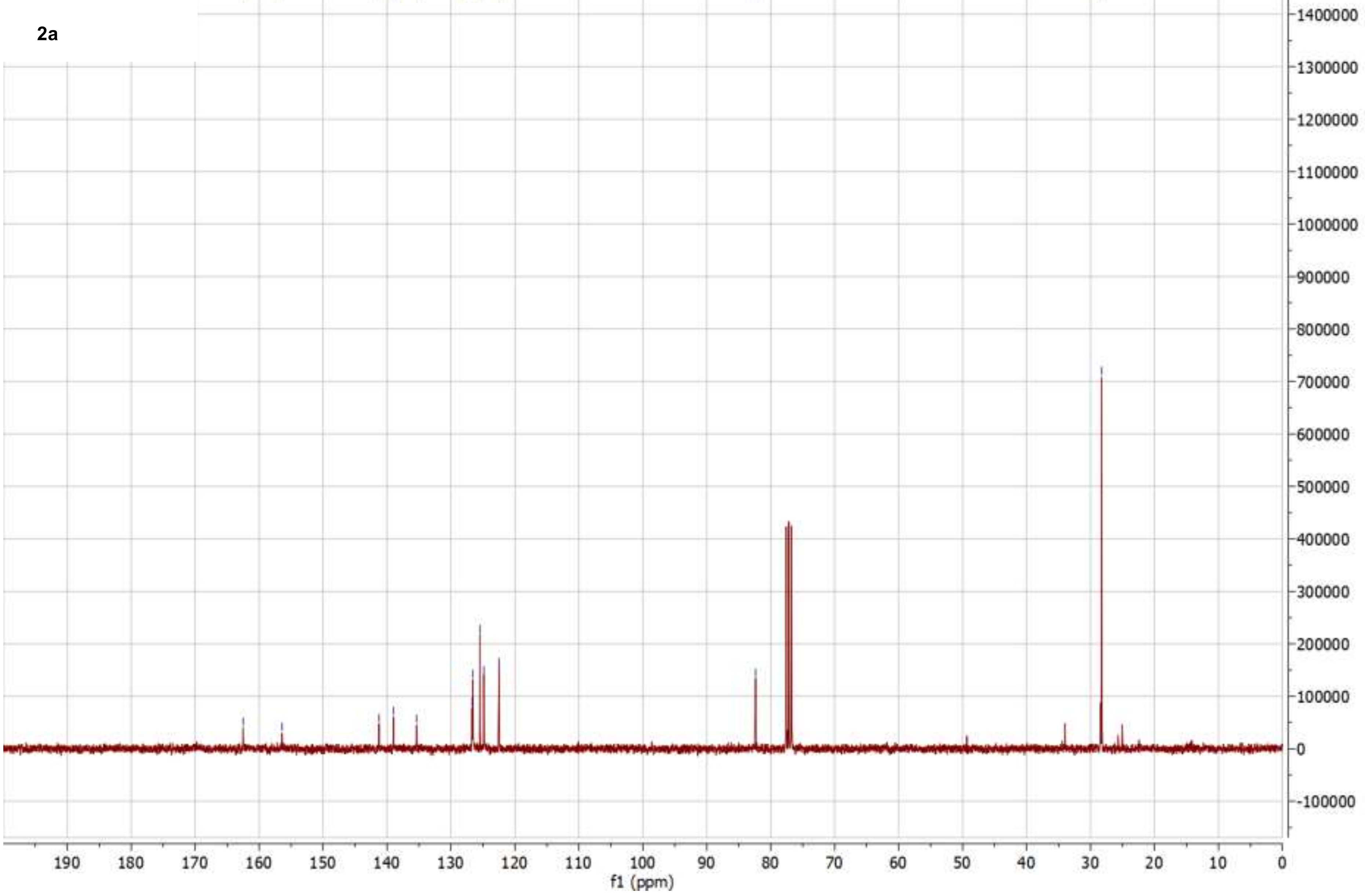
162.51
156.41
141.27
139.01
135.36
126.70
126.56
125.47
124.86
122.52
82.37
28.24
f1 (ppm)

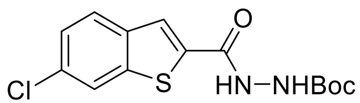
O
Cl
S
HN−NHBoc

2b

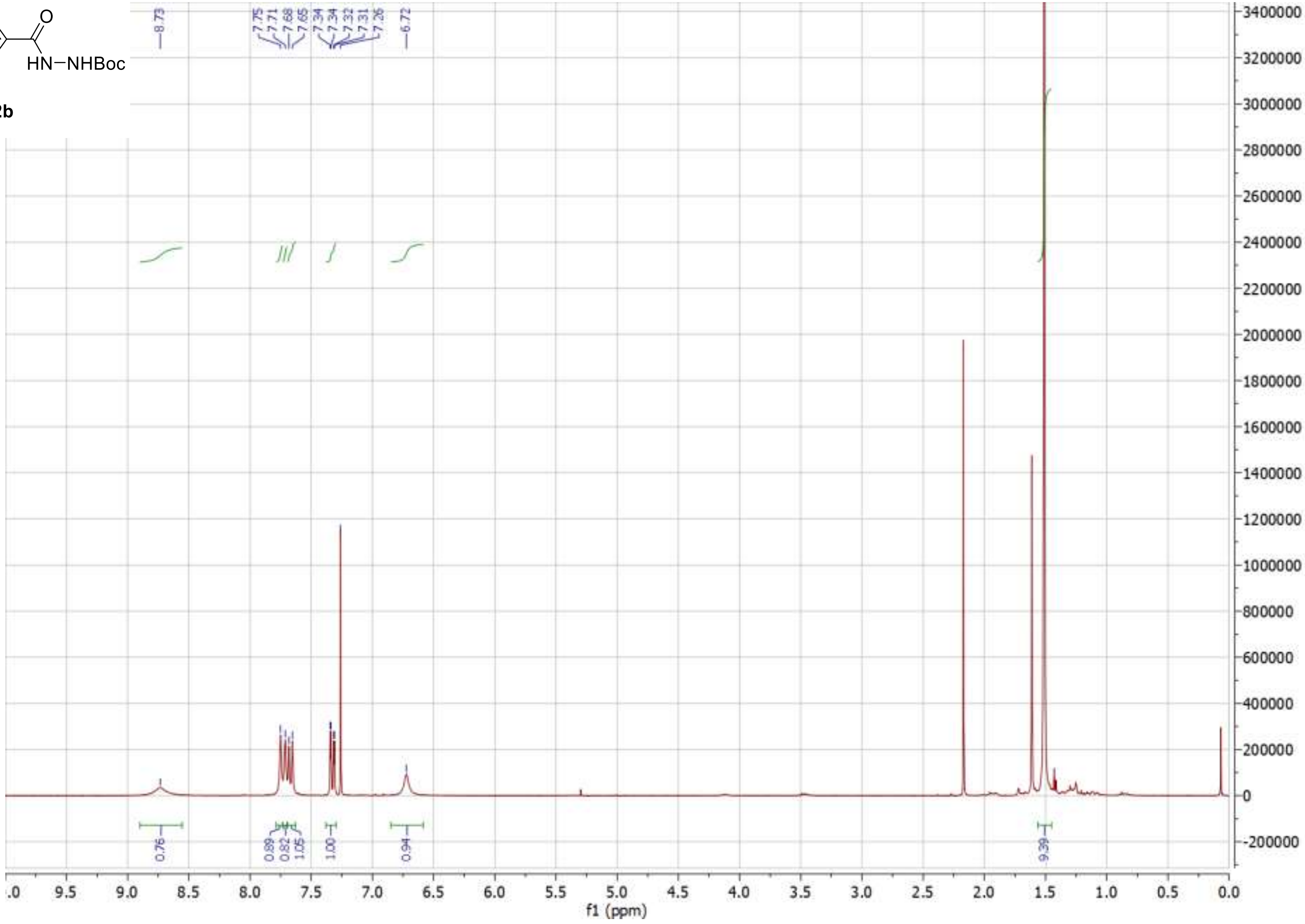
8.73
7.75
7.71
7.68
7.65
7.34
7.34
7.32
7.31
7.26
6.72
0.76
0.89
0.82
1.05
1.00
0.94
9.39
f1 (ppm)

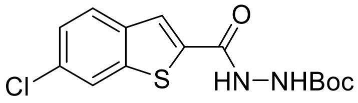
O
Cl
S
HN−NHBoc

2b

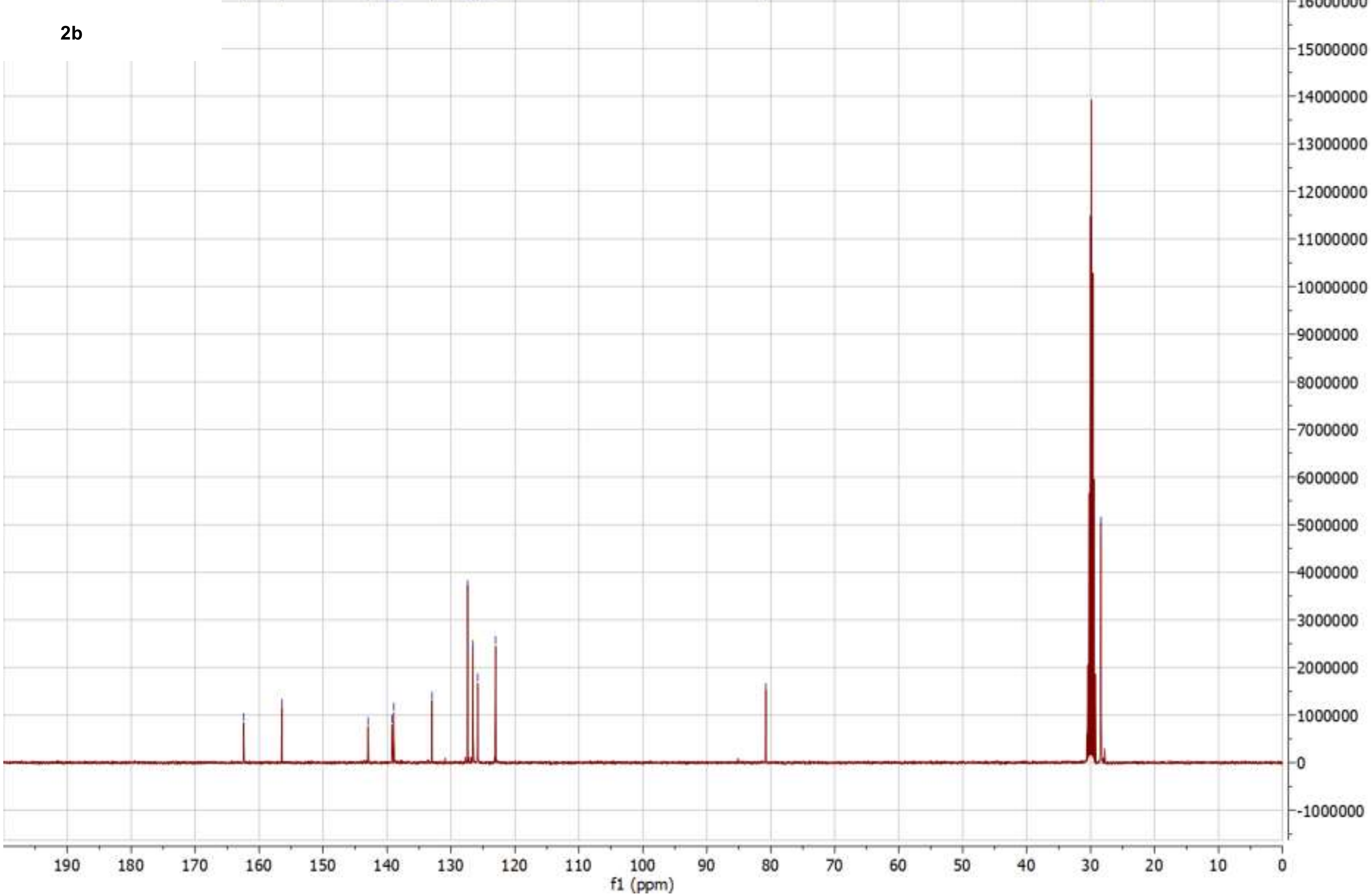
162.42
156.46
142.96
139.20
138.93
132.97
127.41
126.55
125.83
123.03
80.78
28.39
f1 (ppm)

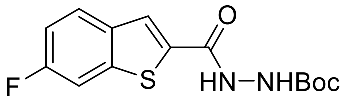

O
F
S
HN−NHBoc

2c

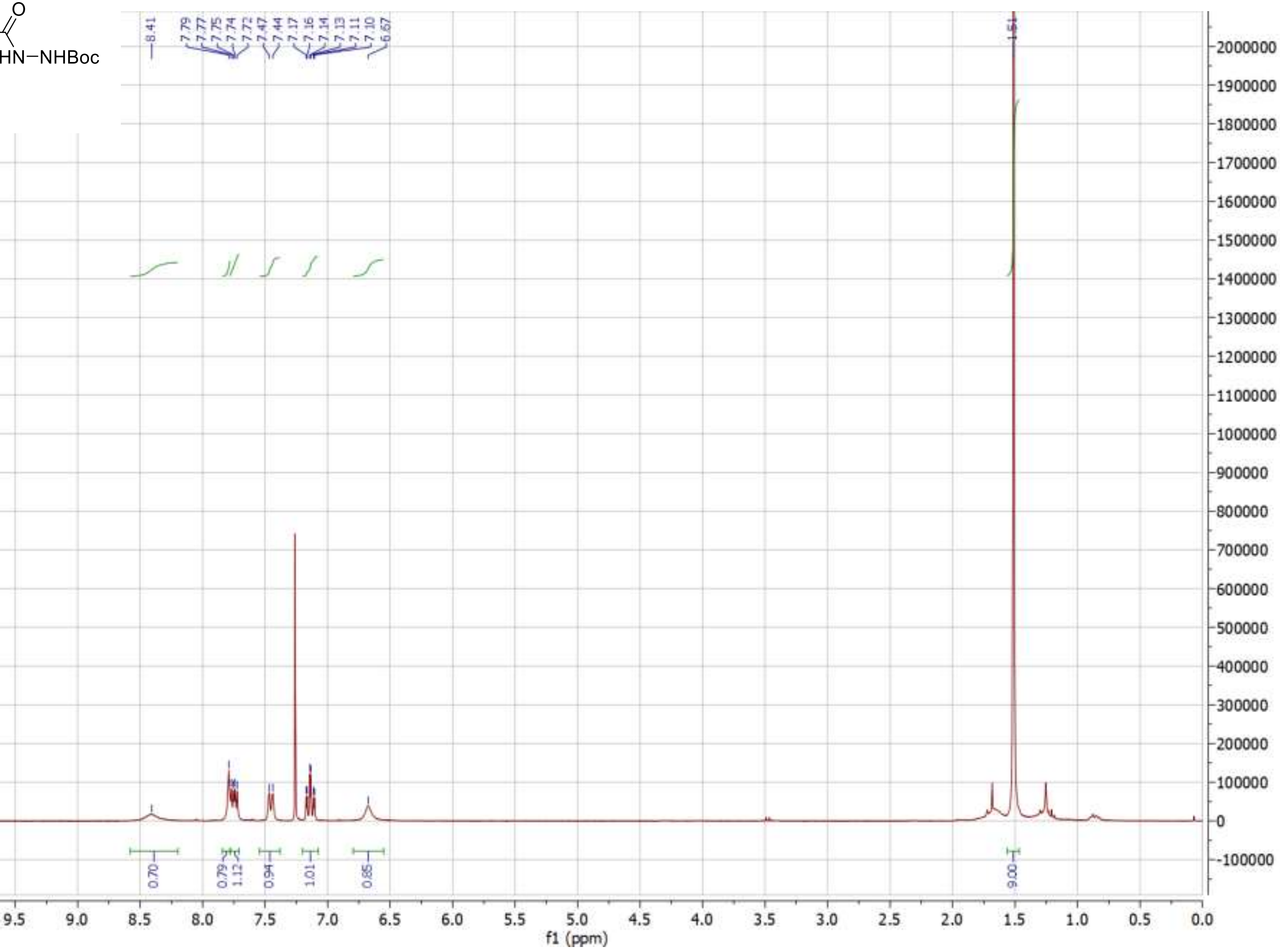

8.41
7.79
7.77
7.75
7.74
7.72
7.47
7.44
7.17
7.16
7.14
7.13
7.11
7.10
6.67
1.51
0.70
0.79
1.12
0.94
1.01
0.85
9.00
f1 (ppm)

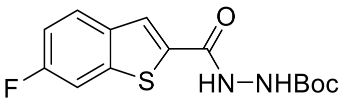
O
F
S
HN−NHBoc

2c

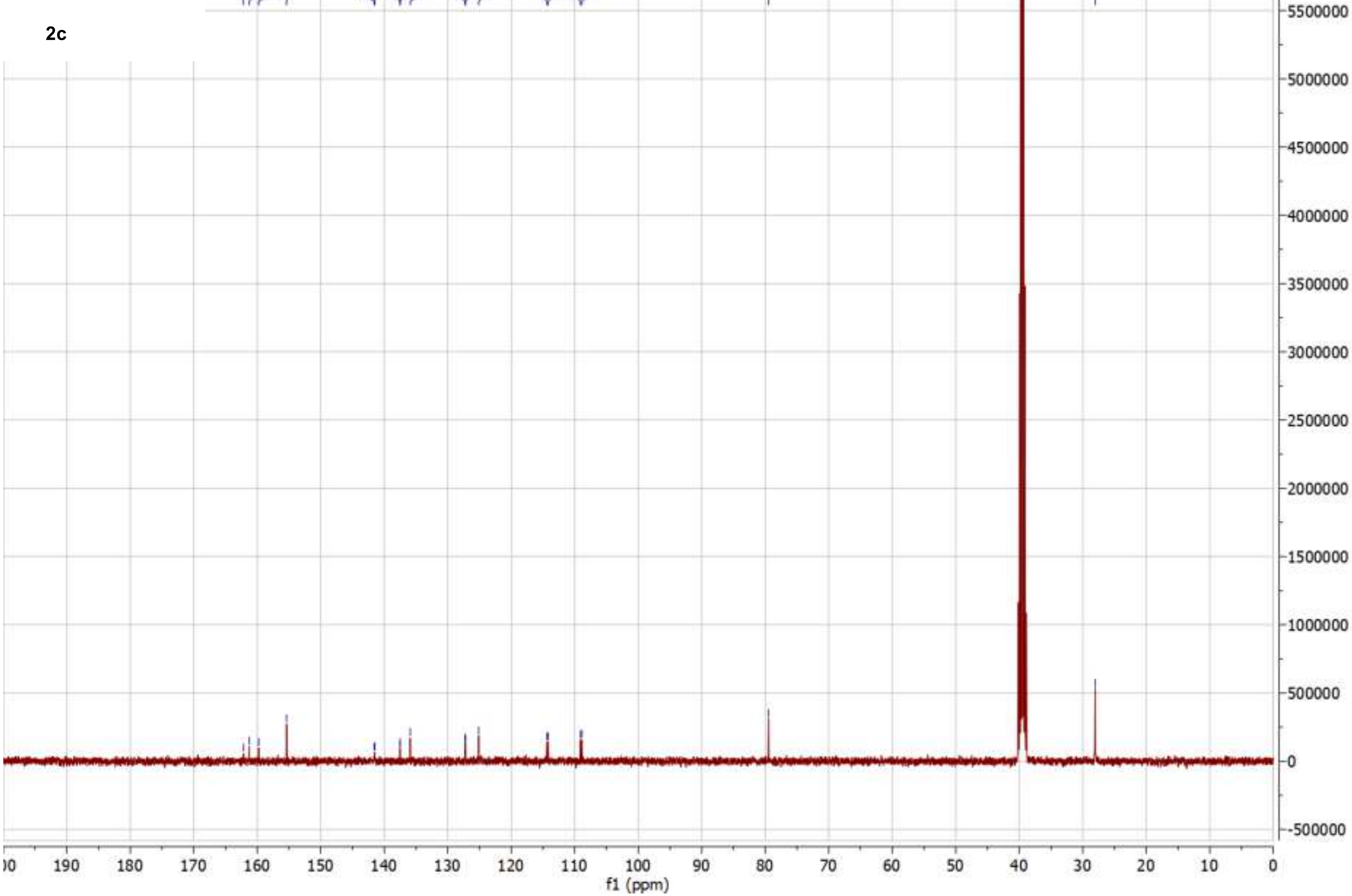
162.19
161.29
159.76
155.39
141.60
141.49
137.54
137.51
135.91
127.28
127.19
125.14
114.39
114.15
109.11
108.85
79.49
28.08
5500000
5000000
4500000
4000000
3500000
3000000
2500000
2000000
1500000
1000000
500000
0
-500000
190
180
170
160
150
140
130
120
110
100
90
80
70
60
50
40
30
20
10
0
f1 (ppm)

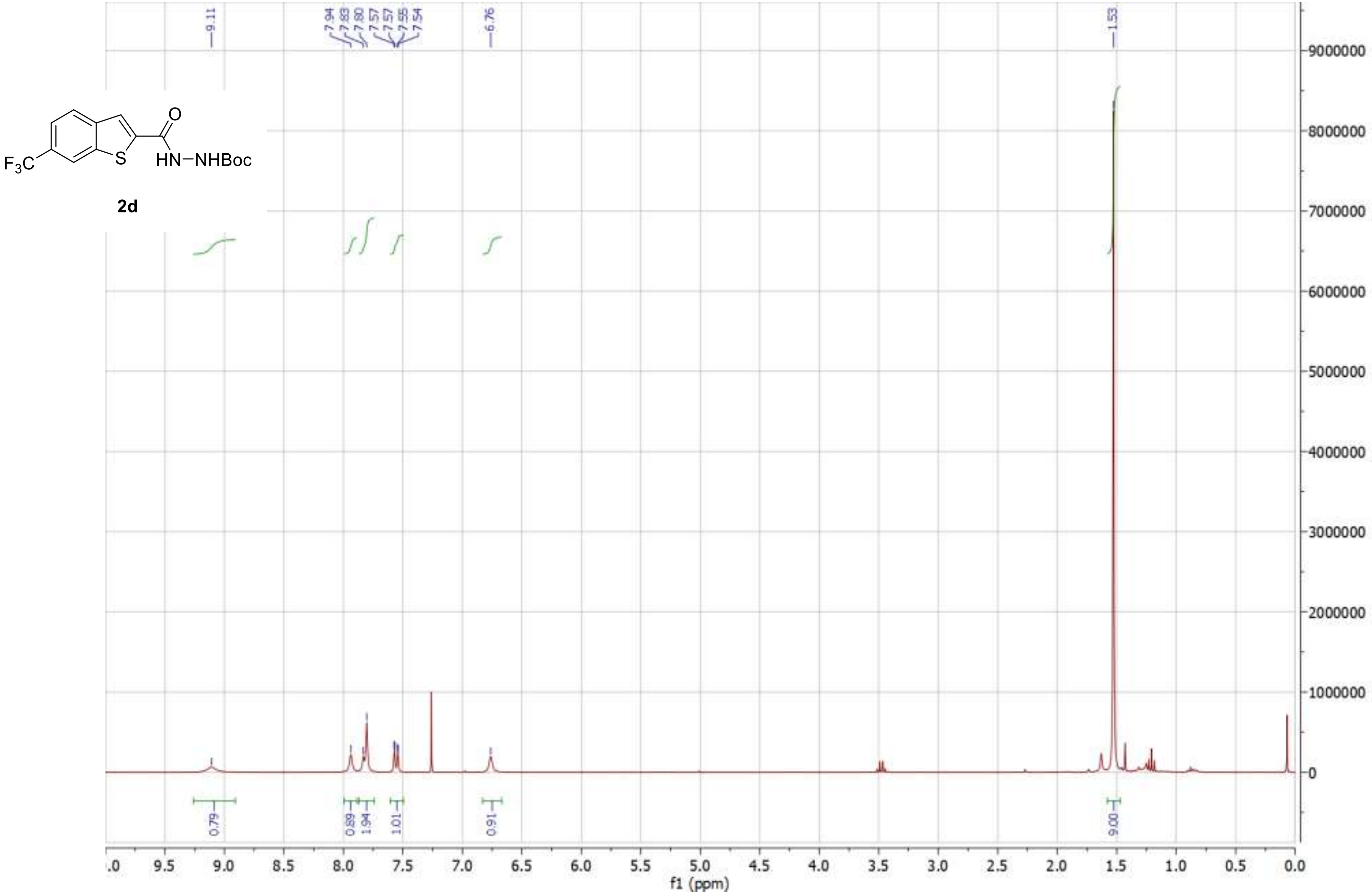

F3C
S
O
HN−NHBoc
2d
9.11
7.94
7.83
7.80
7.57
7.57
7.55
7.54
6.76
1.53
0.79
0.89
1.94
1.01
0.91
9.00
f1 (ppm)
9000000
8000000
7000000
6000000
5000000
4000000
3000000
2000000
1000000
0

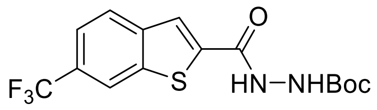
O
F3C
S
HN−NHBoc

2d

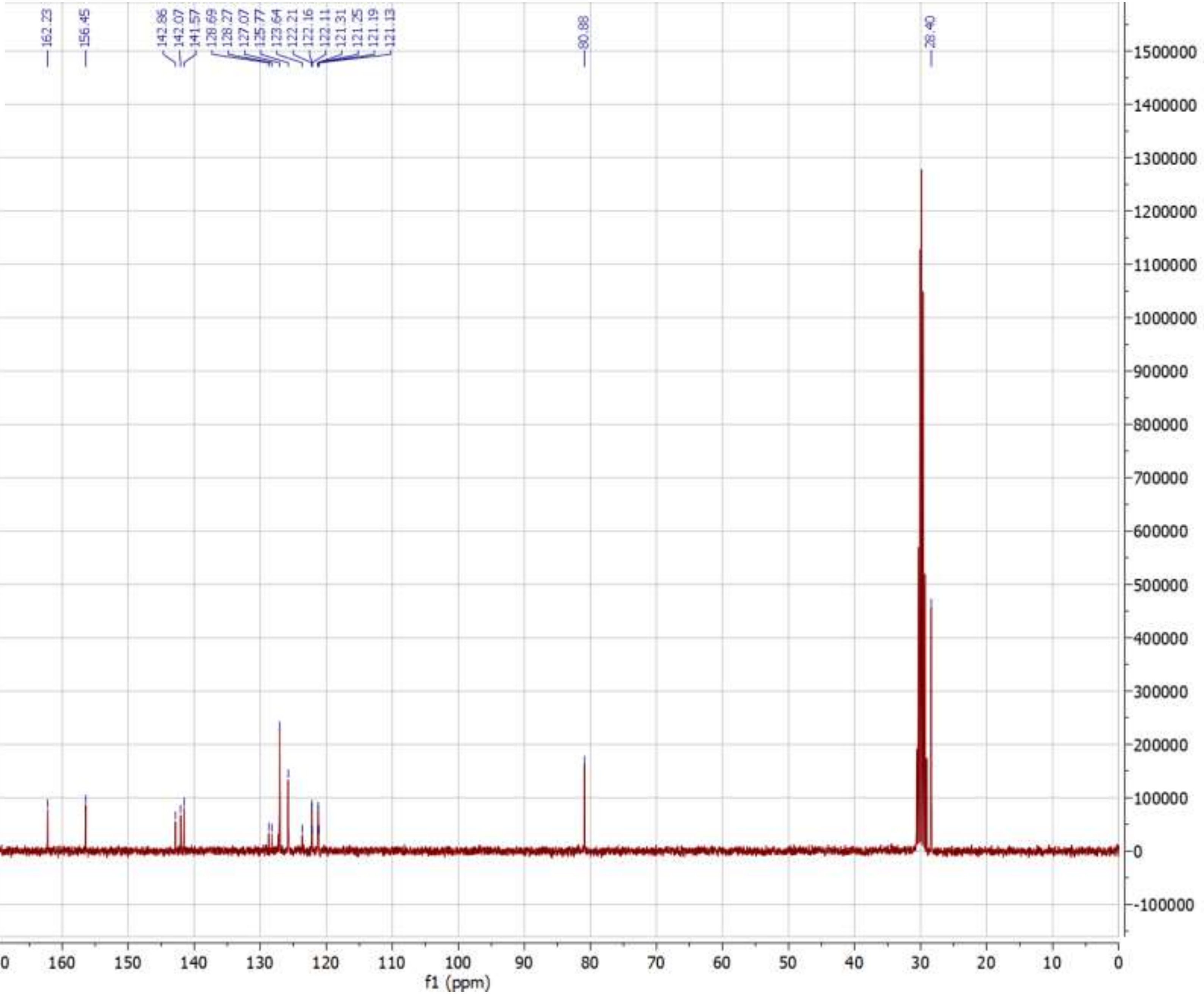
162.23
156.45
142.86
142.07
141.57
128.69
128.27
127.07
125.77
123.64
122.21
122.16
122.11
121.31
121.25
121.19
121.13
80.88
28.40
1500000
1400000
1300000
1200000
1100000
1000000
900000
800000
700000
600000
500000
400000
300000
200000
100000
0
-100000
190 180 170 160 150 140 130 120 110 100 90 80 70 60 50 40 30 20 10 0
f1 (ppm)

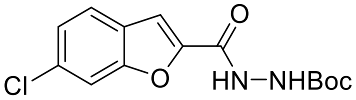
Cl
O
O
HN−NHBoc

2e

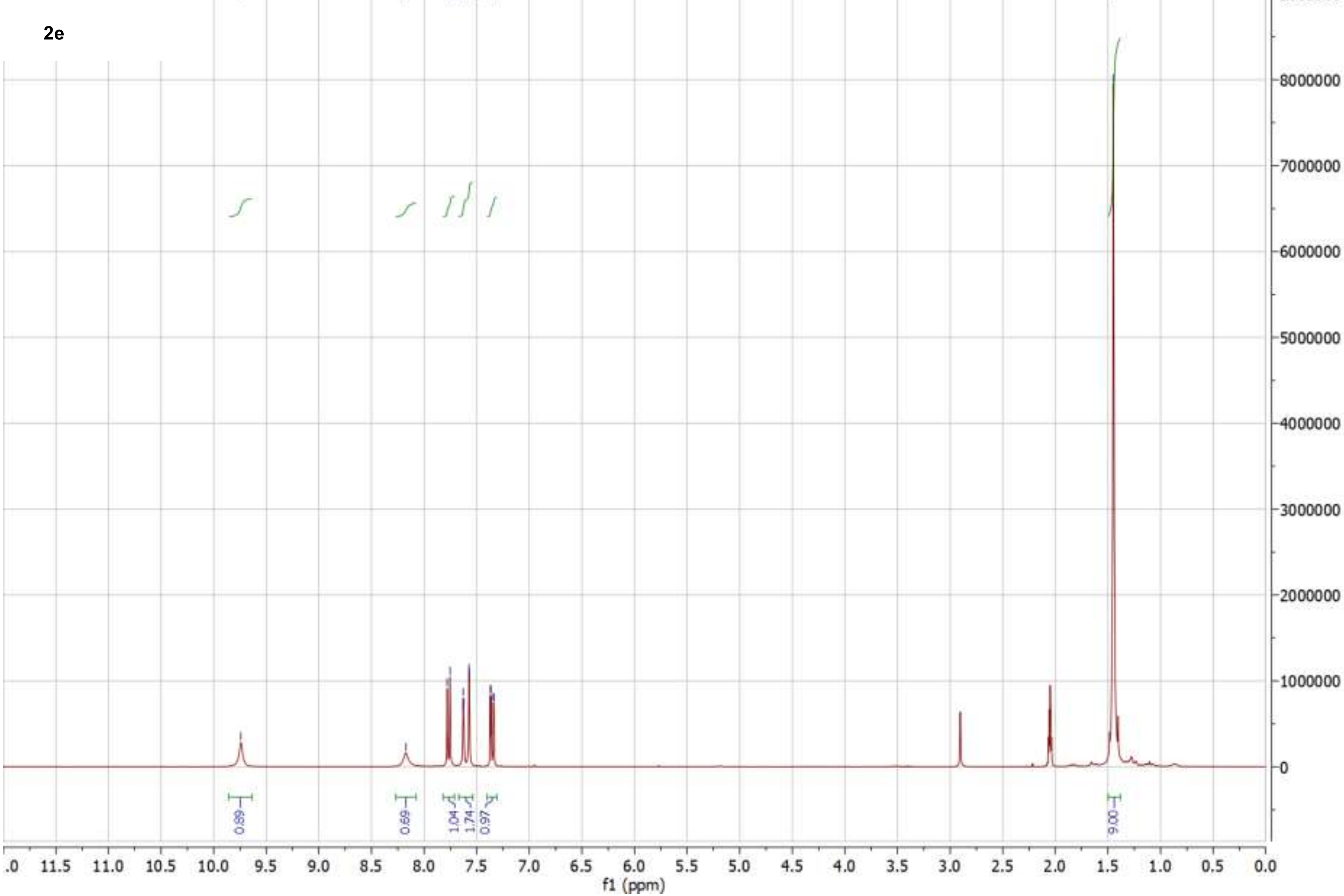
9.74
8.17
7.78
7.75
7.63
7.63
7.62
7.57
7.57
7.37
7.36
7.34
7.34
1.45
0.89
0.89
1.04
1.74
0.97
9.00
9000000
8000000
7000000
6000000
5000000
4000000
3000000
2000000
1000000
0
.0 11.5 11.0 10.5 10.0 9.5 9.0 8.5 8.0 7.5 7.0 6.5 6.0 5.5 5.0 4.5 4.0 3.5 3.0 2.5 2.0 1.5 1.0 0.5 0.0
f1 (ppm)

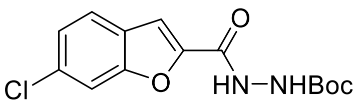
O
Cl
O
HN−NHBoc

2e

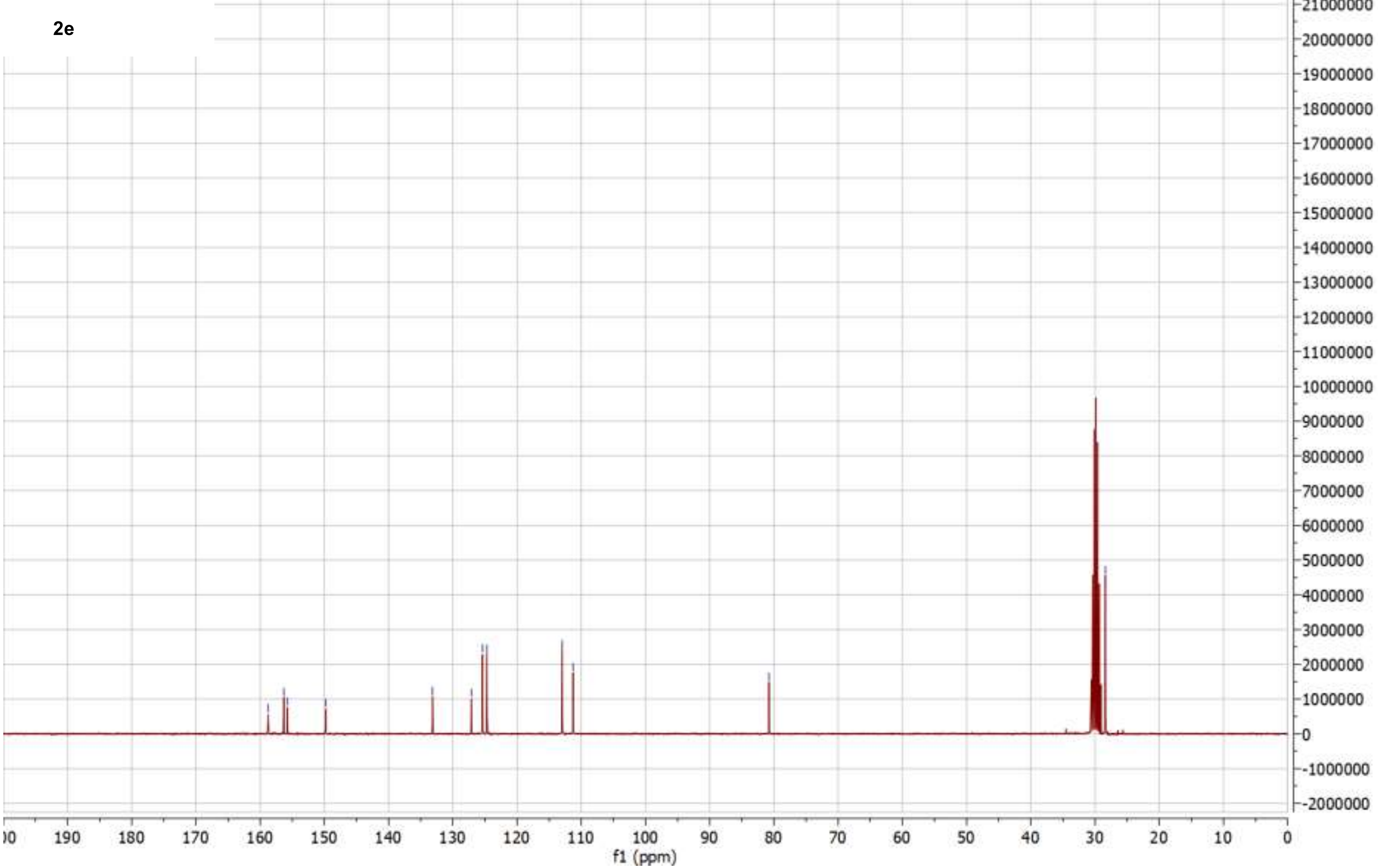
158.77
156.30
155.75
149.81
133.17
127.08
125.38
124.68
112.96
111.27
80.77
28.39
23000000
22000000
21000000
20000000
19000000
18000000
17000000
16000000
15000000
14000000
13000000
12000000
11000000
10000000
9000000
8000000
7000000
6000000
5000000
4000000
3000000
2000000
1000000
0
-1000000
-2000000
190
180
170
160
150
140
130
120
110
100
90
80
70
60
50
40
30
20
10
0
f1 (ppm)

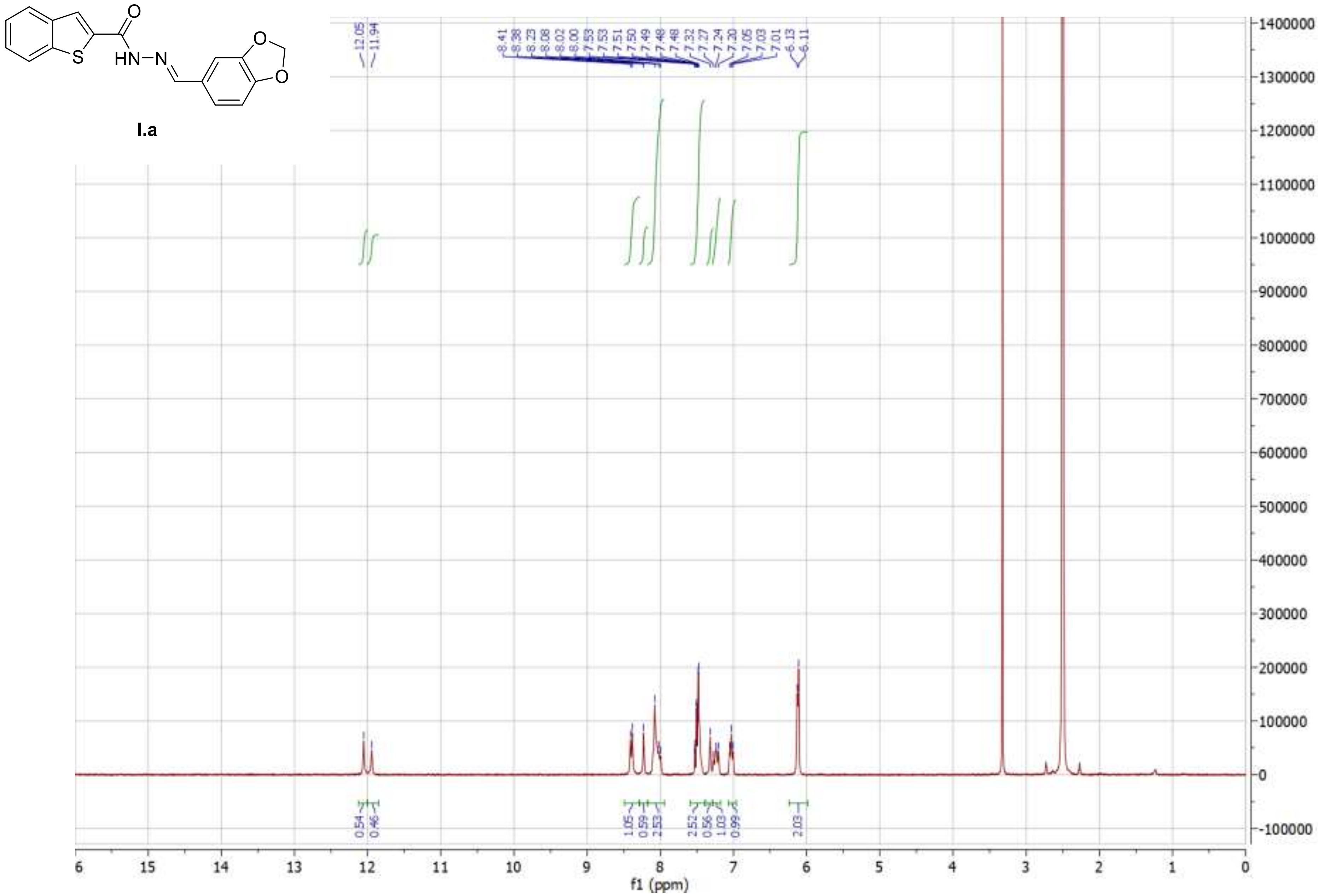
O
S
HN–N
O
O

I.a

12.05
11.94
8.41
8.38
8.23
8.08
8.02
8.00
7.53
7.53
7.51
7.50
7.49
7.48
7.48
7.32
7.27
7.24
7.20
7.05
7.03
7.01
6.13
6.11
0.54
0.46
1.05
0.59
2.53
2.52
0.56
1.03
0.99
2.03
f1 (ppm)

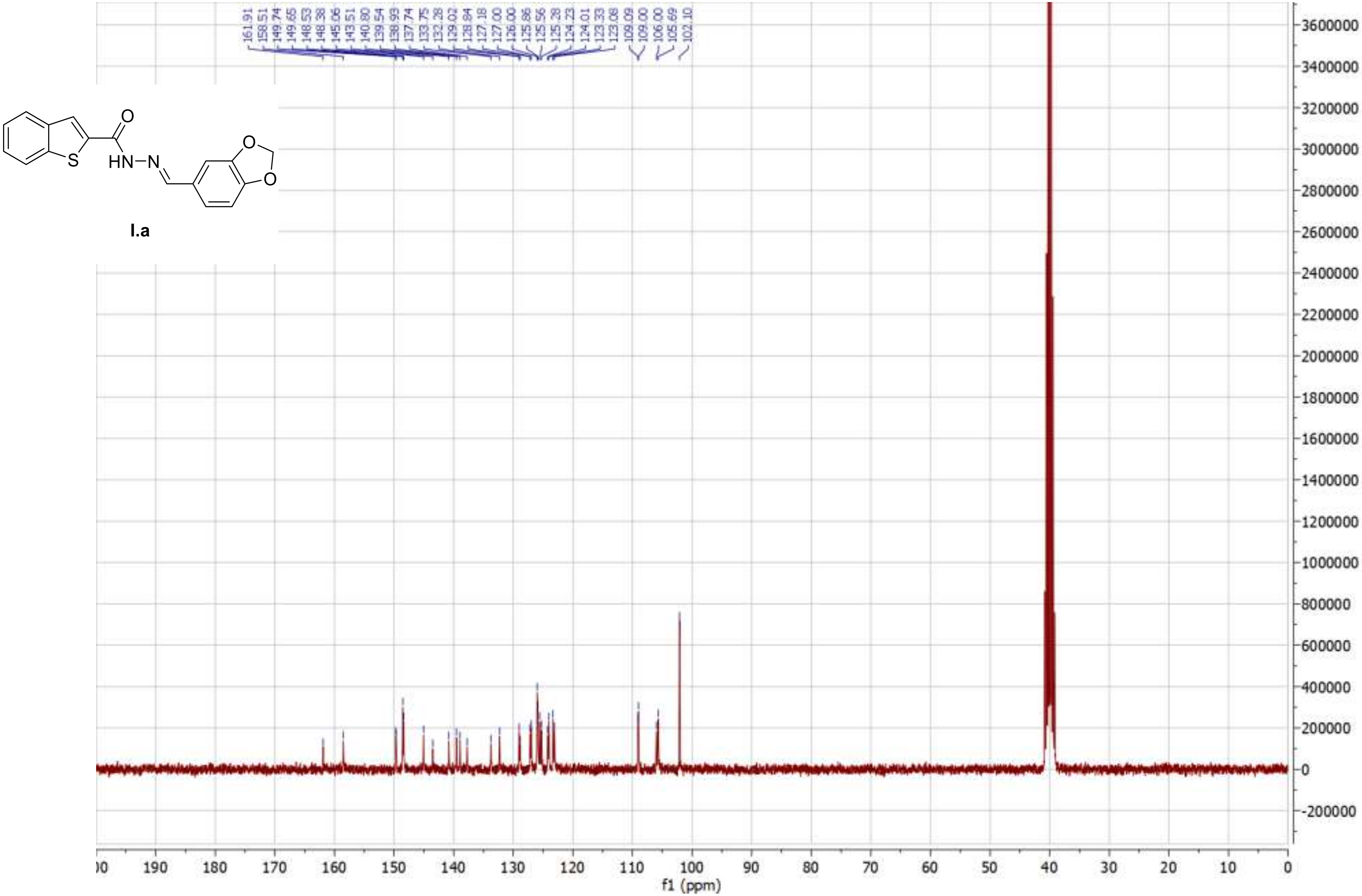
O
S
HN–N
O
O
161.91
158.51
149.74
149.65
148.53
148.38
145.06
143.51
140.80
139.54
138.93
137.74
133.75
132.28
129.02
128.84
127.18
127.00
126.00
125.86
125.56
125.28
124.23
124.01
123.33
123.08
109.09
109.00
106.00
105.69
102.10
f1 (ppm)

I.a

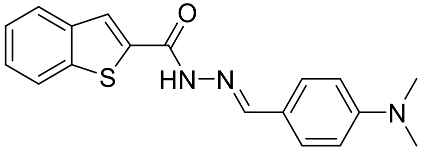

O
S
HN–N
N

I.b

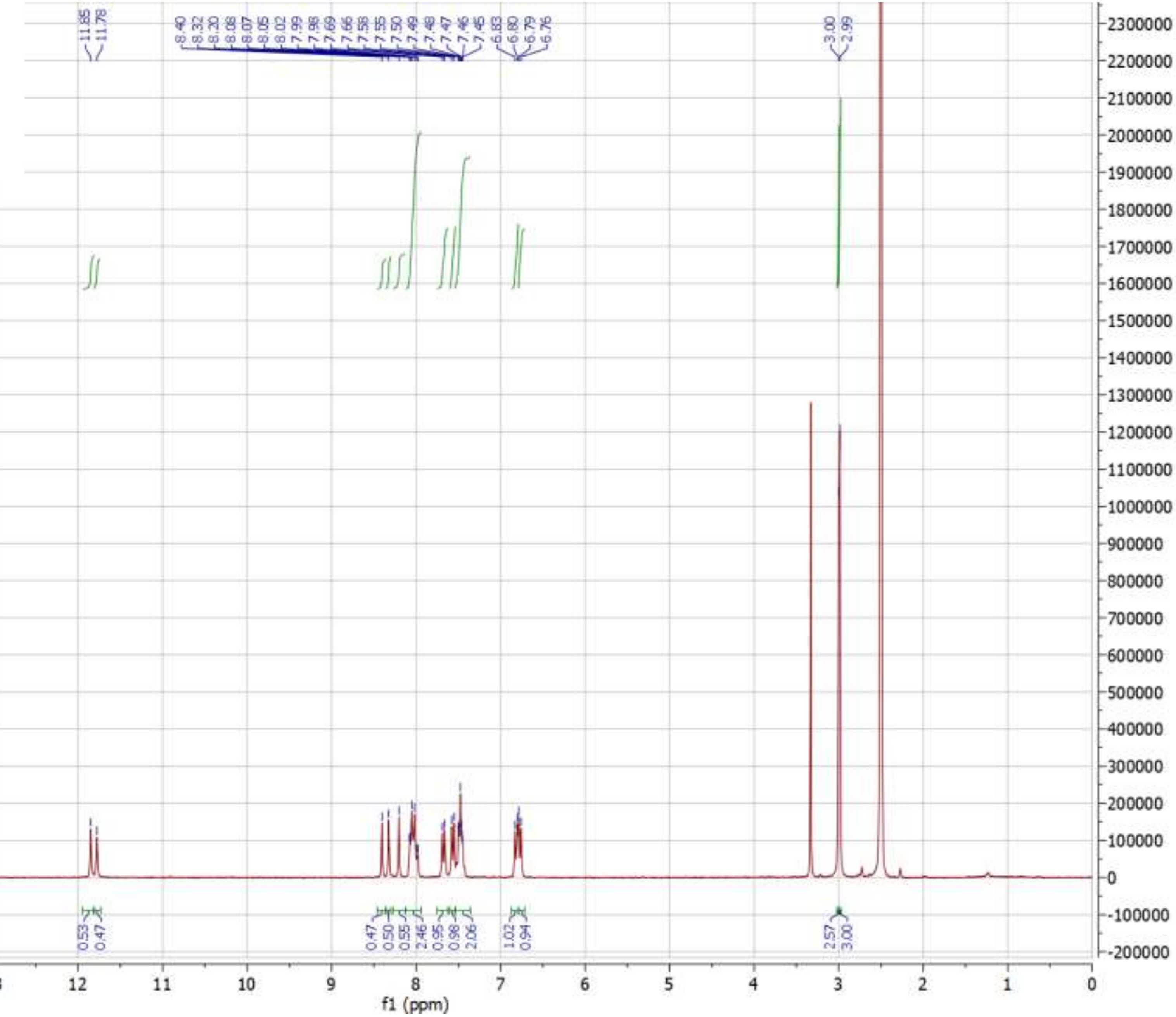

11.85
11.78
8.40
8.32
8.20
8.08
8.07
8.05
8.02
7.99
7.98
7.69
7.66
7.58
7.55
7.50
7.49
7.48
7.47
7.46
7.45
6.83
6.80
6.79
6.76
3.00
2.99
0.53
0.47
0.47
0.50
0.55
2.46
0.95
0.98
2.06
1.02
0.94
2.57
3.00
f1 (ppm)

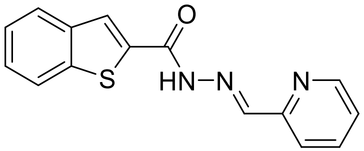
O
HN−N
N
S

I.c

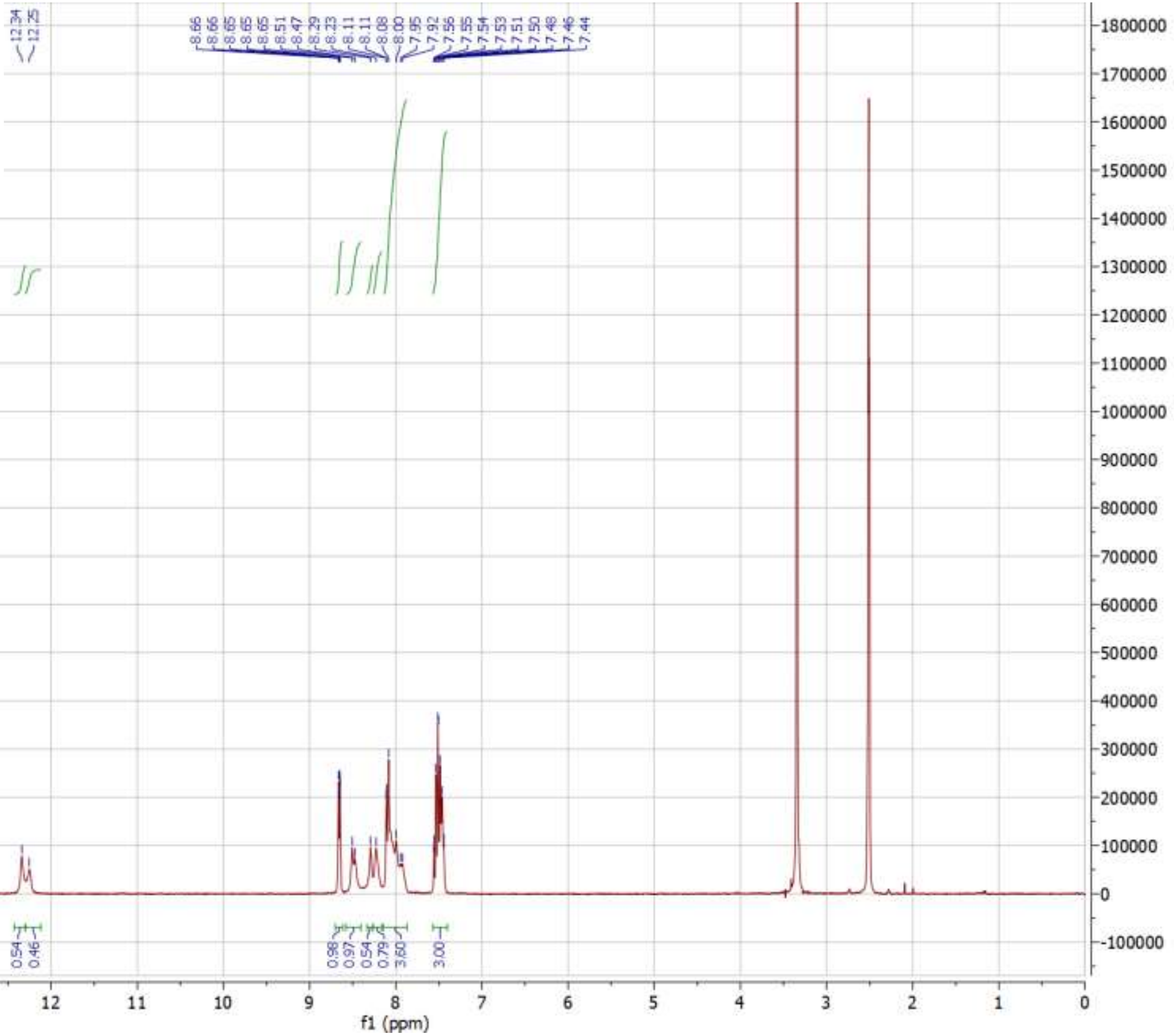
12.34
12.25
8.66
8.66
8.65
8.65
8.65
8.51
8.47
8.29
8.23
8.11
8.11
8.08
8.00
7.95
7.92
7.56
7.55
7.54
7.53
7.51
7.50
7.48
7.46
7.44
0.54
0.46
0.98
0.97
0.54
0.79
3.60
3.00
f1 (ppm)

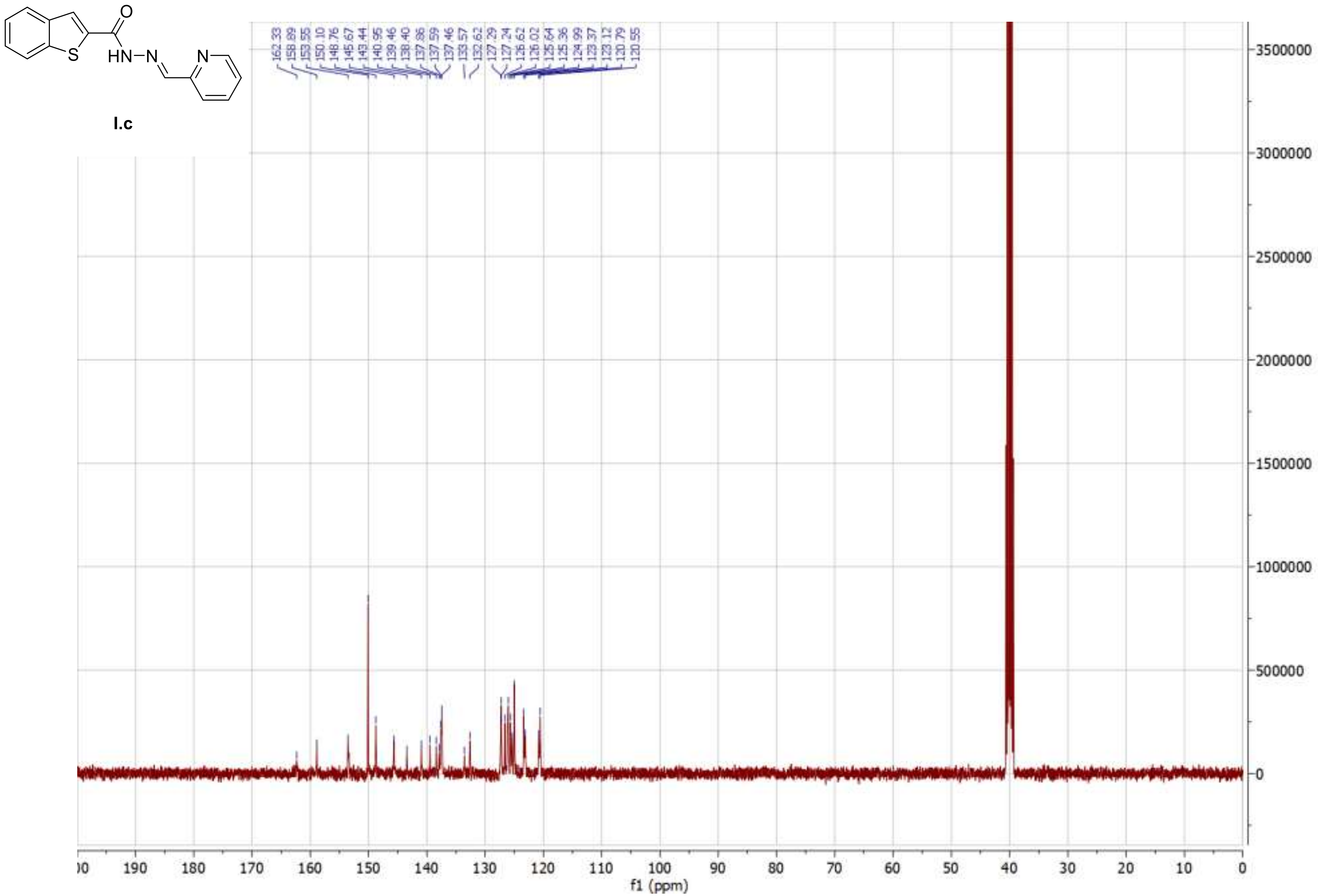
O
S
HN–N
N

162.33
158.89
153.55
150.10
148.76
145.67
143.44
140.95
139.46
138.40
137.86
137.59
137.46
133.57
132.62
127.29
127.24
126.62
126.02
125.64
125.36
124.99
123.37
123.12
120.79
120.55
3500000
3000000
2500000
2000000
1500000
1000000
500000
0
190
180
170
160
150
140
130
120
110
100
90
80
70
60
50
40
30
20
10
0
f1 (ppm)

I.c

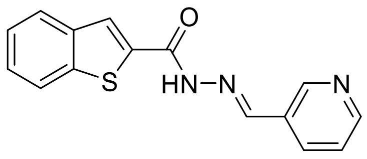
O
S
HN
N
N

I.d

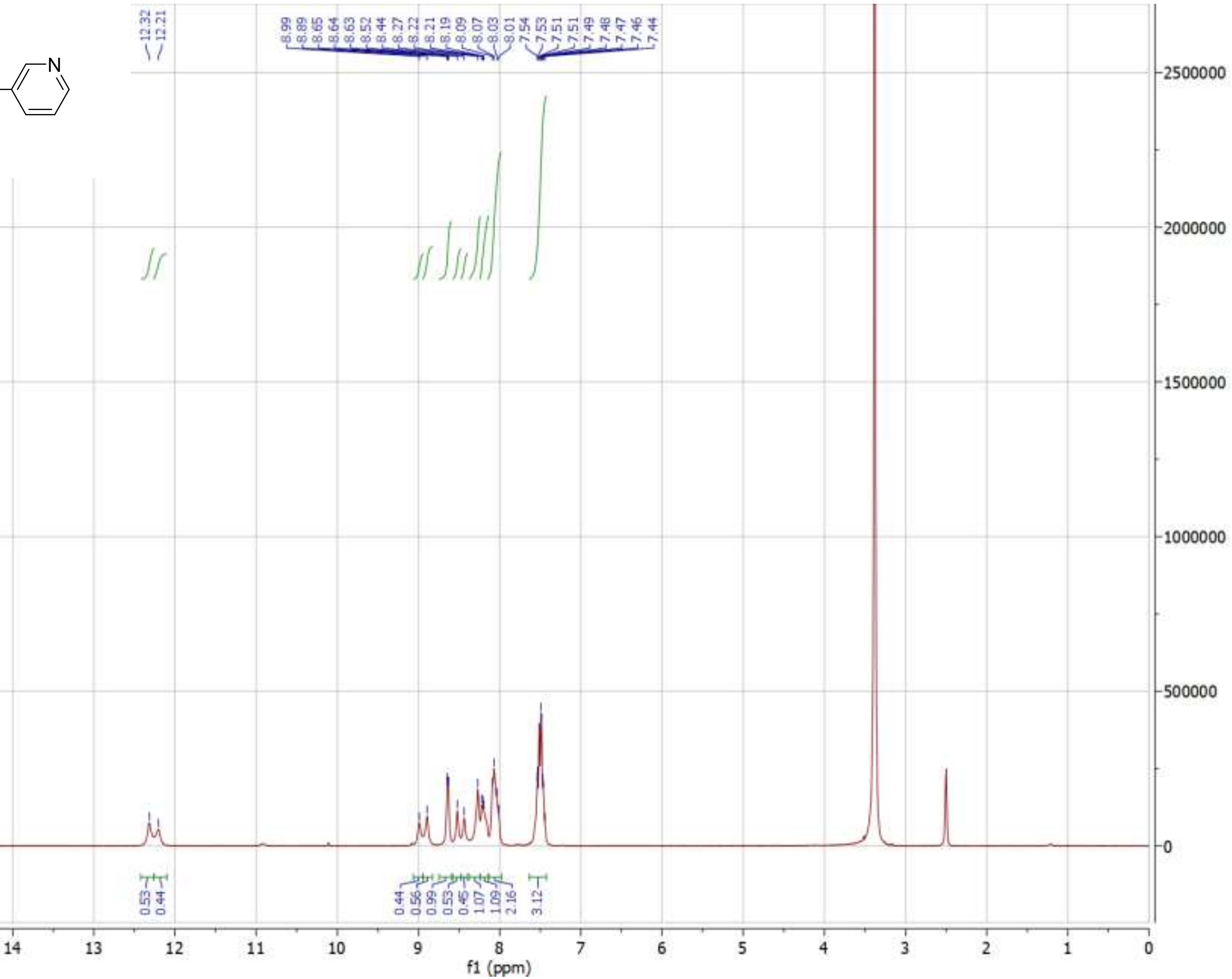
12.32
12.21
8.99
8.89
8.65
8.64
8.63
8.52
8.44
8.27
8.22
8.21
8.19
8.09
8.07
8.03
8.01
7.54
7.53
7.51
7.51
7.49
7.48
7.47
7.46
7.44
2500000
2000000
1500000
1000000
500000
0
0.53
0.44
0.44
0.56
0.99
0.53
0.45
1.07
1.09
2.16
3.12
6
15
14
13
12
11
10
9
8
7
6
5
4
3
2
1
0
f1 (ppm)

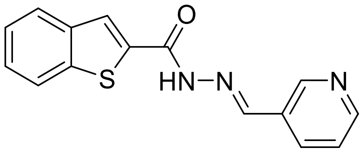
O
S
HN–N
N

I.d

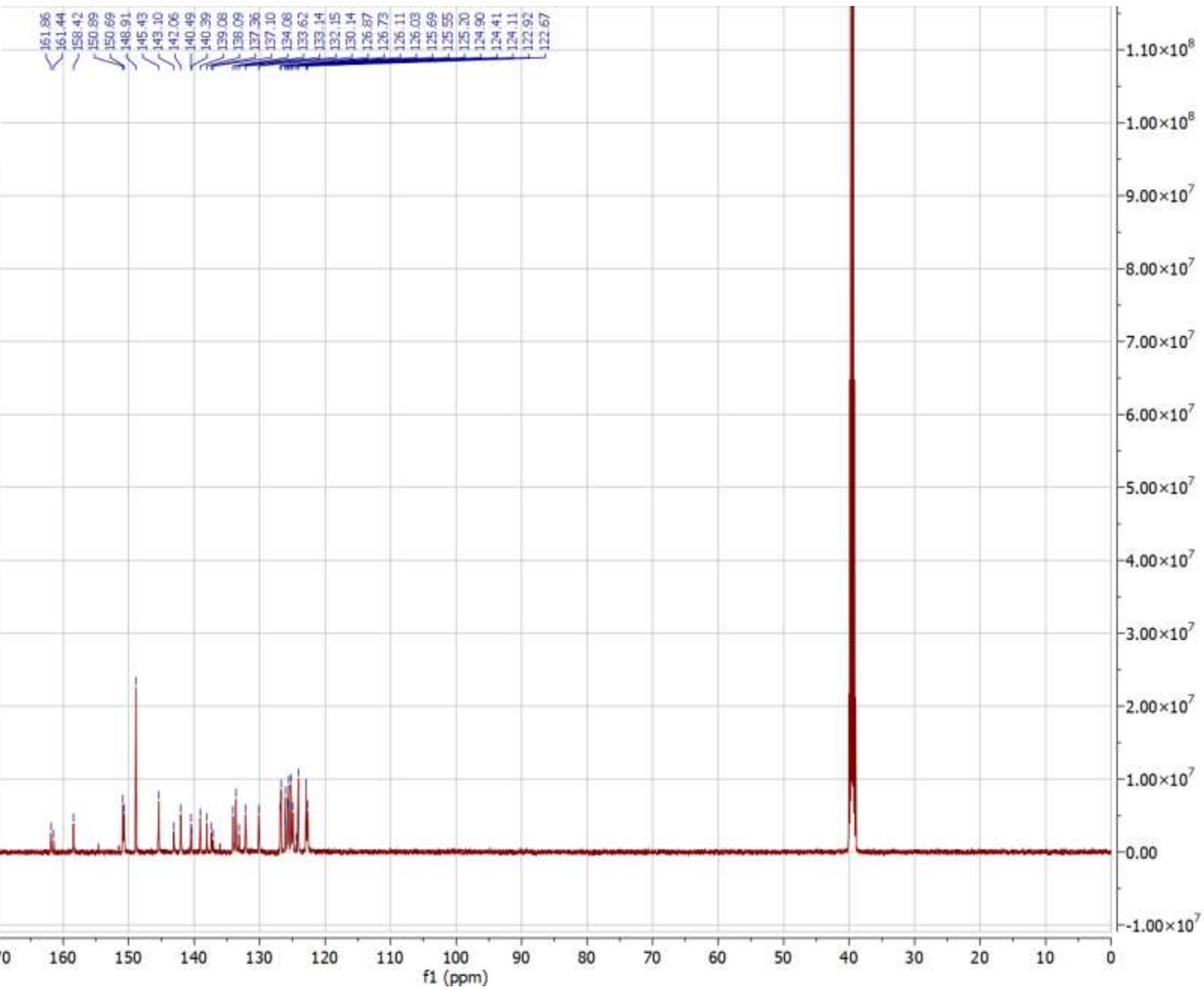
161.86
161.44
158.42
150.89
150.69
148.91
145.43
143.10
142.06
140.49
140.39
139.08
138.09
137.36
137.10
134.08
133.62
133.14
132.15
130.14
126.87
126.73
126.11
126.03
125.69
125.55
125.20
124.90
124.41
124.11
122.92
122.67
f1 (ppm)

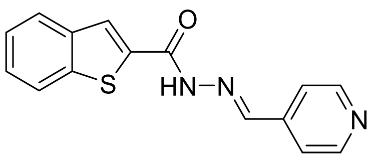

I.e

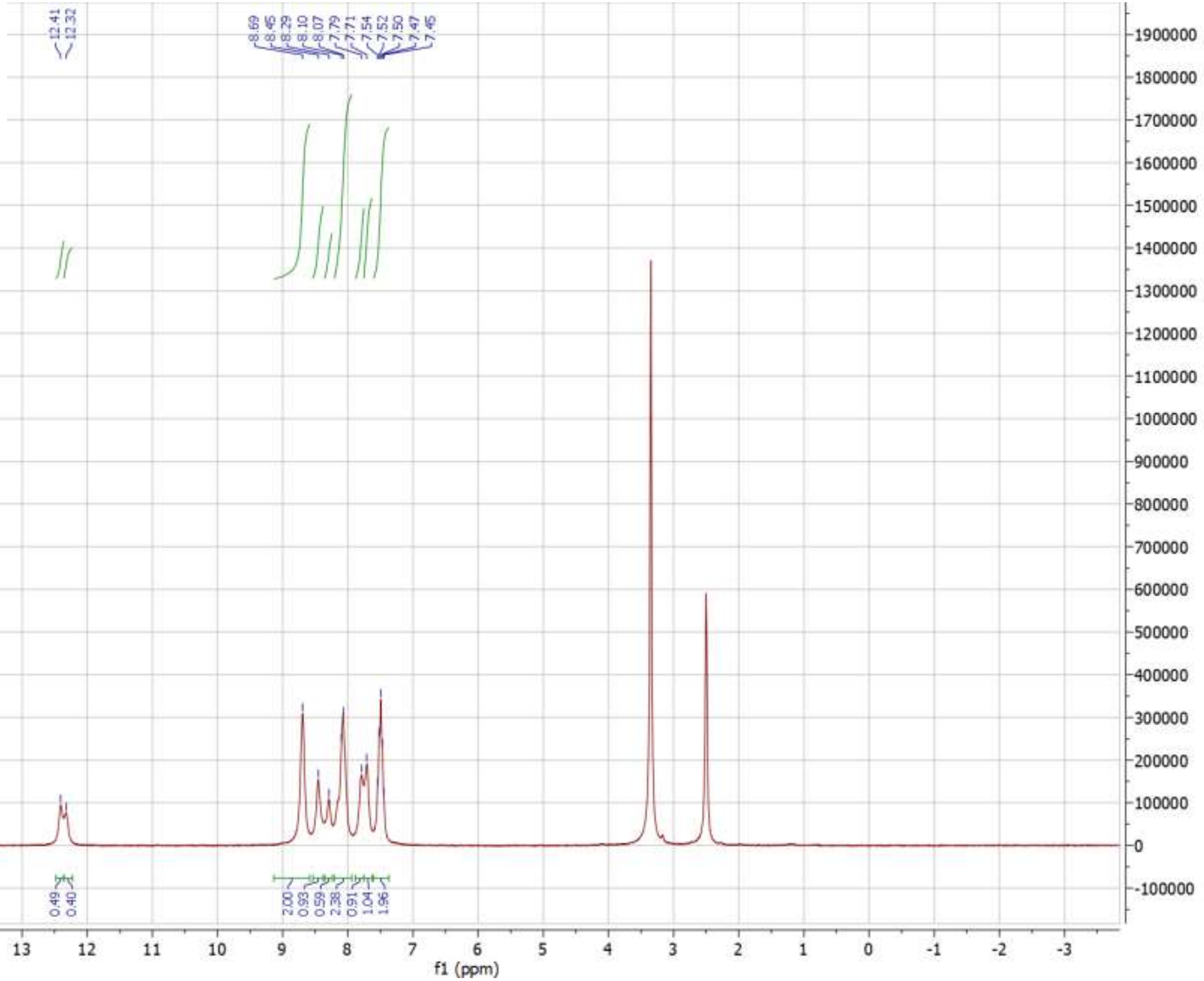

12.41
12.32
8.69
8.45
8.29
8.10
8.07
7.79
7.71
7.54
7.52
7.50
7.47
7.45
0.49
0.40
2.00
0.93
0.59
2.38
0.91
1.04
1.96
f1 (ppm)
16 15 14 13 12 11 10 9 8 7 6 5 4 3 2 1 0 -1 -2 -3
1900000 1800000 1700000 1600000 1500000 1400000 1300000 1200000 1100000 1000000 900000 800000 700000 600000 500000 400000 300000 200000 100000 0 -100000

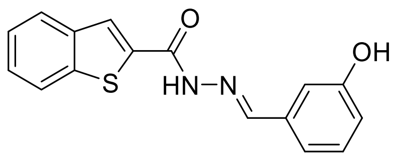
O
OH
S
HN–N

I.f

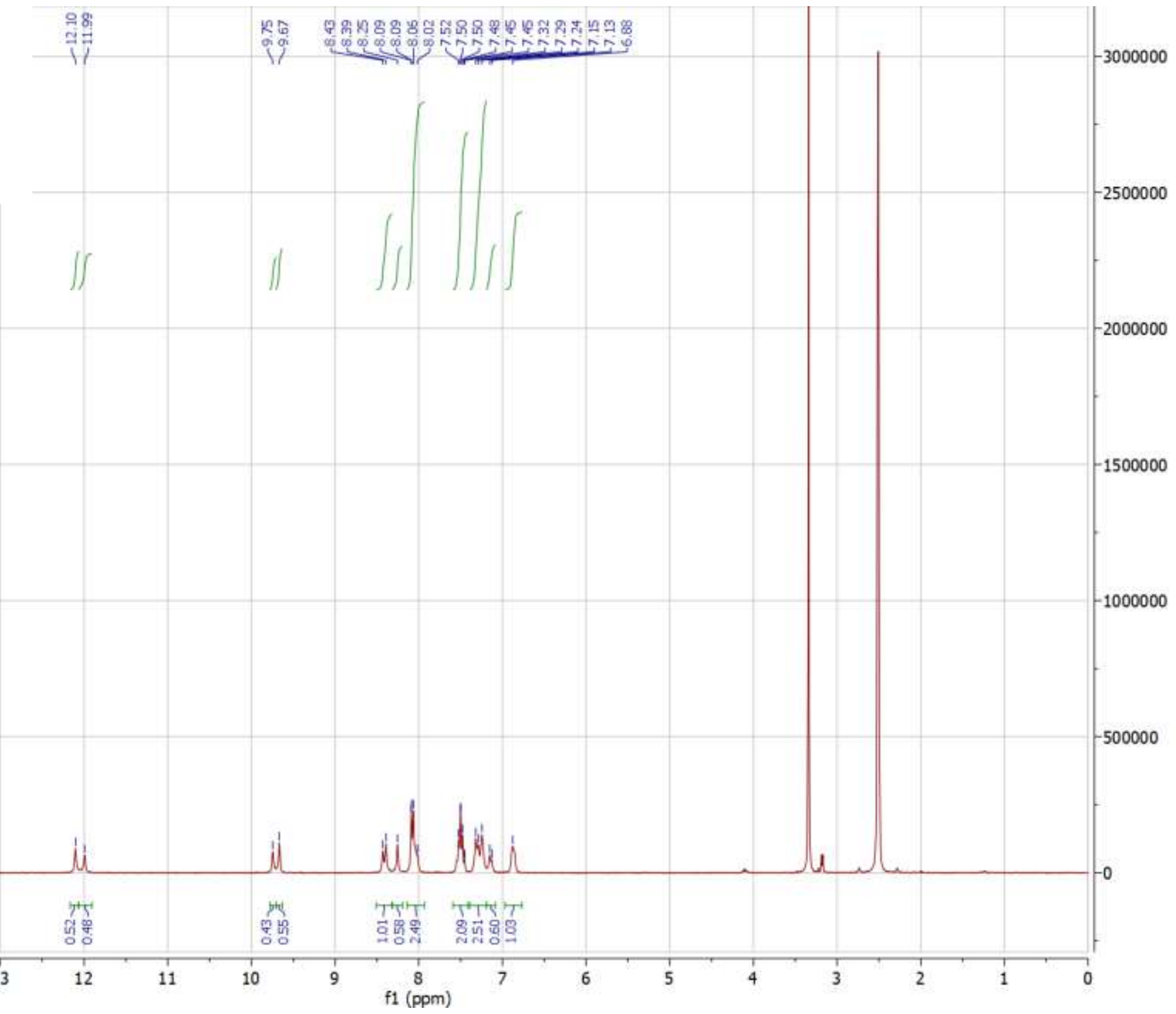
12.10
11.99
9.75
9.67
8.43
8.39
8.25
8.09
8.09
8.06
8.02
7.52
7.50
7.50
7.48
7.45
7.45
7.32
7.29
7.24
7.15
7.13
6.88
0.52
0.48
0.43
0.55
1.01
0.58
2.49
2.09
2.51
0.60
1.03
f1 (ppm)

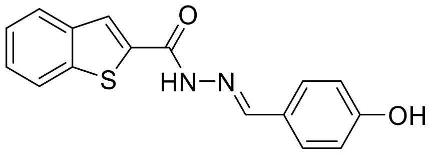

I.g

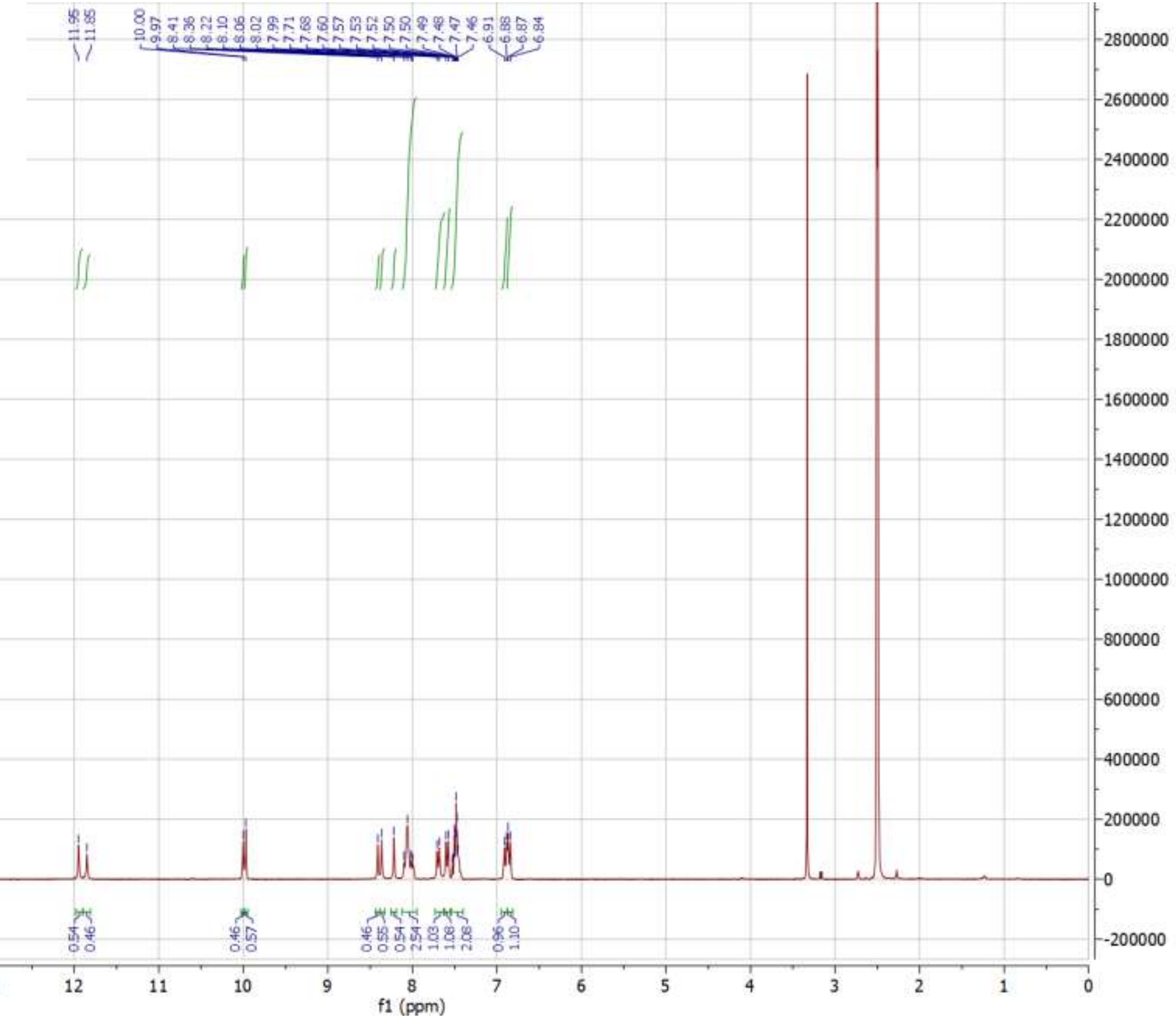

11.95
11.85
10.00
9.97
8.41
8.36
8.22
8.10
8.06
8.02
7.99
7.71
7.68
7.60
7.57
7.53
7.52
7.50
7.50
7.49
7.48
7.47
7.46
6.91
6.88
6.87
6.84
0.54
0.46
0.46
0.57
0.46
0.55
0.54
2.54
1.03
1.08
2.08
0.96
1.10
f1 (ppm)

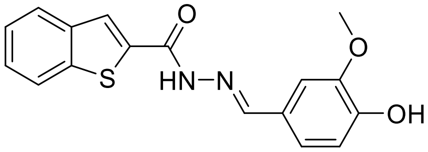
O
HN–N
S
O
OH

I.h

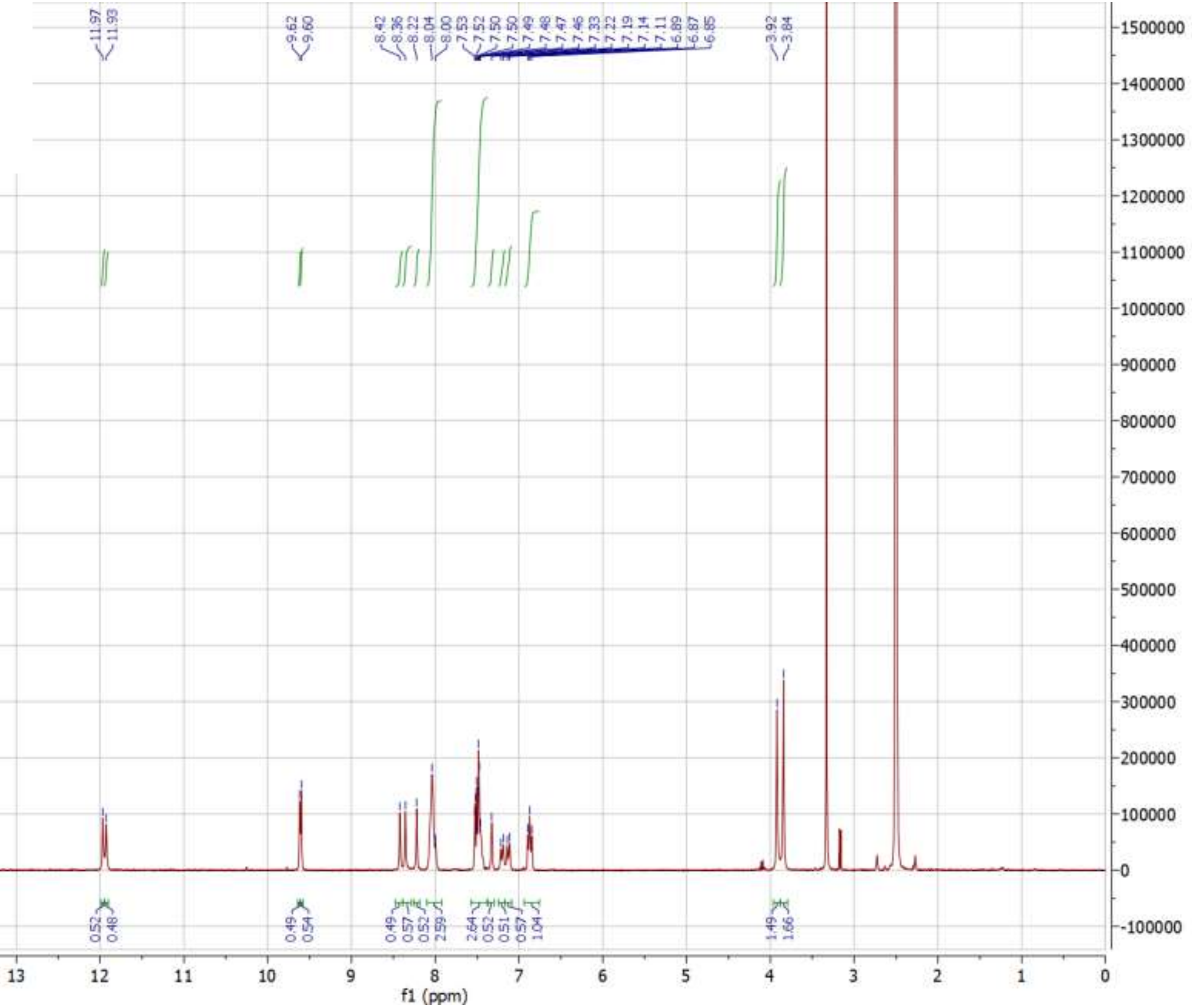
11.97
11.93
9.62
9.60
8.42
8.36
8.22
8.04
8.00
7.53
7.52
7.50
7.50
7.49
7.48
7.47
7.46
7.33
7.22
7.19
7.14
7.11
6.89
6.87
6.85
3.92
3.84
0.52
0.48
0.49
0.54
0.49
0.57
0.52
2.59
2.64
0.52
0.51
0.57
1.04
1.49
1.66
f1 (ppm)

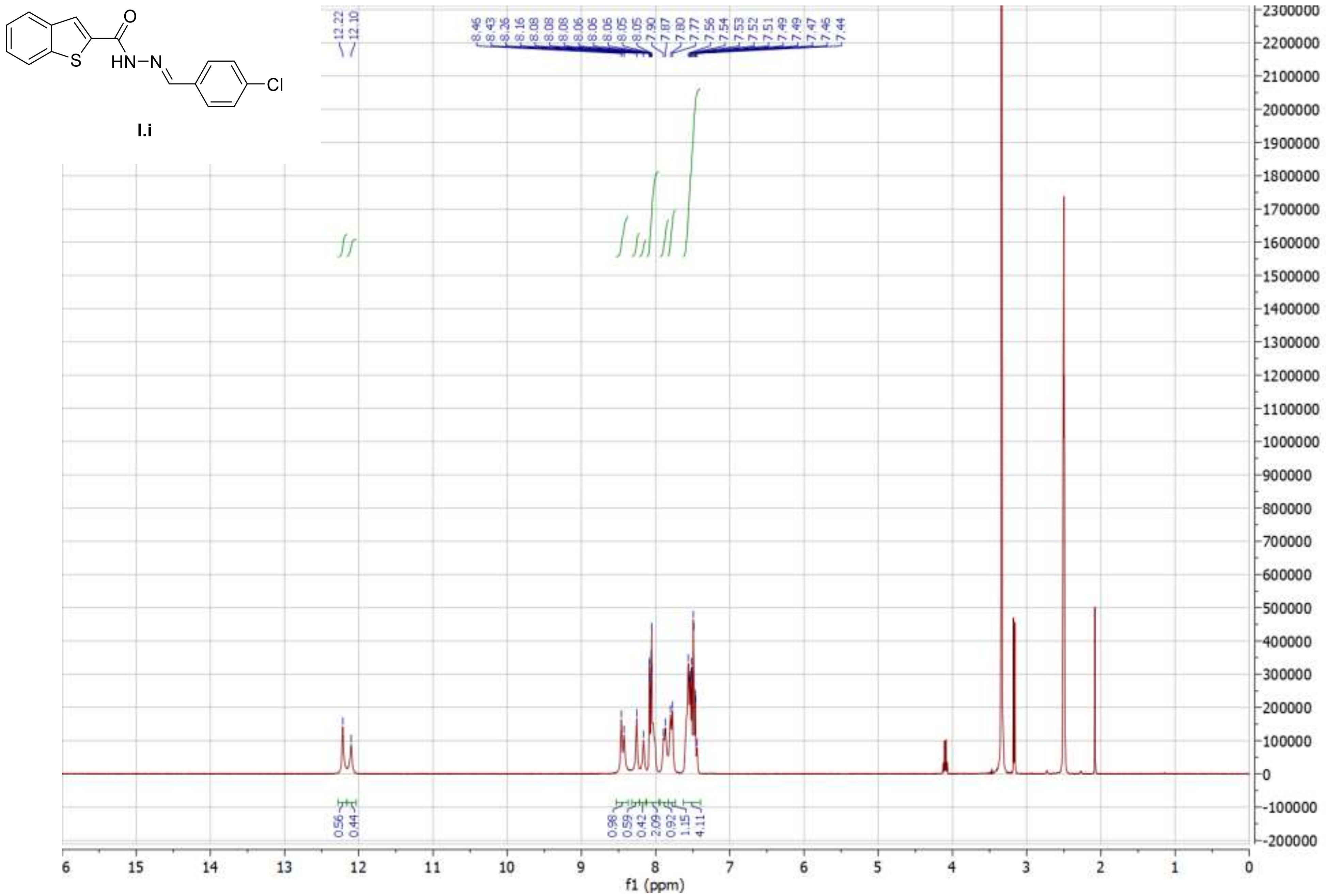

12.22
12.10
8.46
8.43
8.36
8.16
8.08
8.08
8.08
8.06
8.06
8.06
8.05
8.05
7.90
7.87
7.80
7.77
7.56
7.54
7.53
7.52
7.51
7.49
7.49
7.47
7.46
7.44
0.56
0.44
0.98
0.59
0.42
2.09
0.92
1.15
4.11
f1 (ppm)

I.i

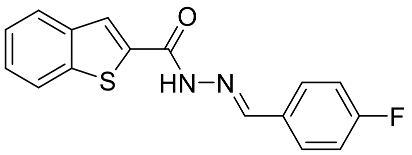

I.j

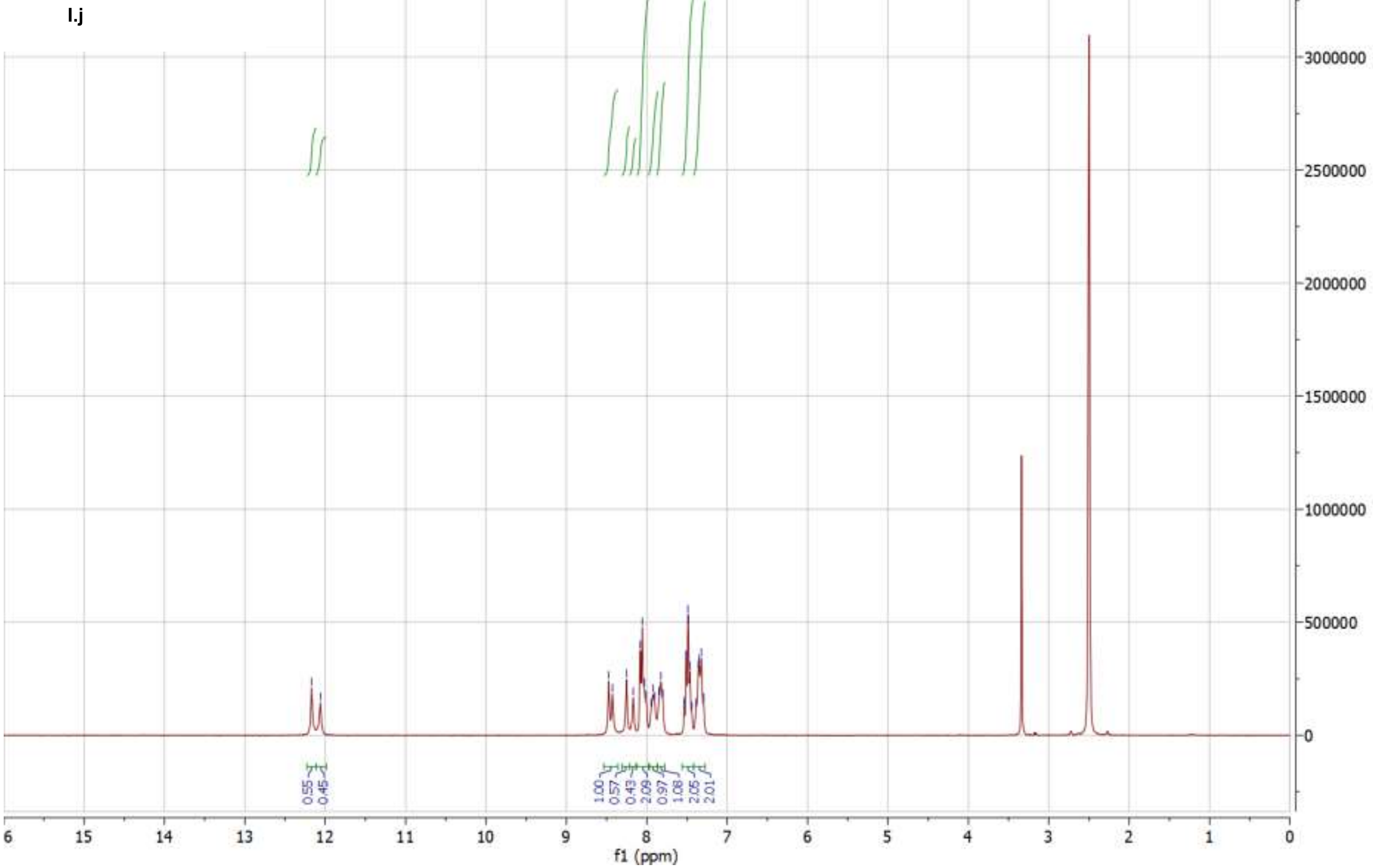

12.17
12.06
8.47
8.43
8.25
8.17
8.08
8.06
8.03
8.01
7.95
7.92
7.90
7.85
7.83
7.80
7.54
7.53
7.51
7.51
7.49
7.48
7.46
7.44
7.39
7.36
7.35
7.32
7.29
0.55
0.45
1.00
0.57
0.43
2.09
0.97
1.08
2.05
2.01
f1 (ppm)

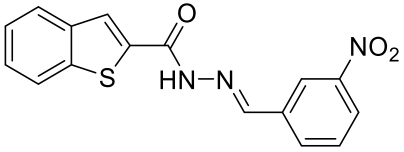
O
NO2
S
HN–N

I.k

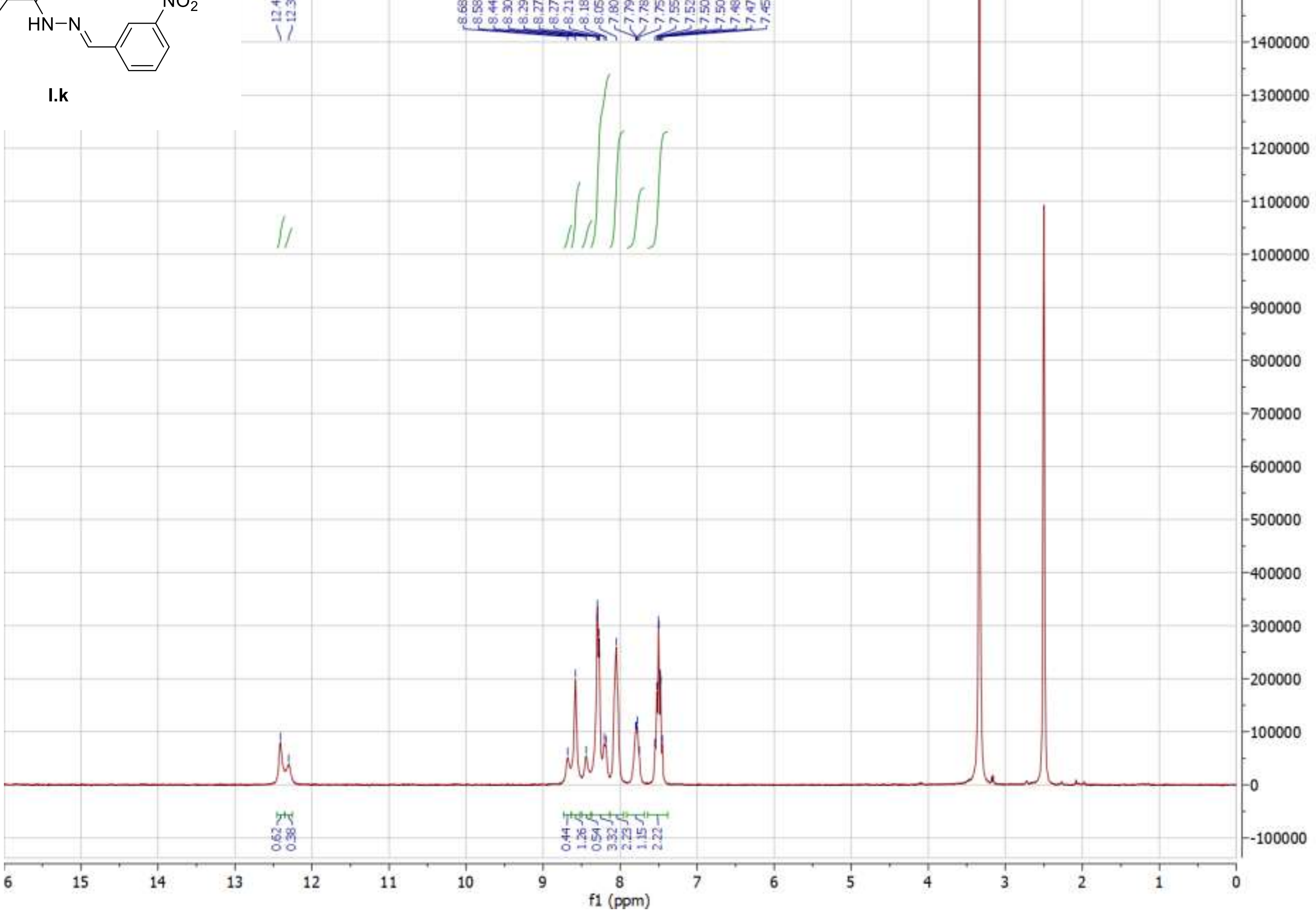
12.41
12.30
8.68
8.58
8.44
8.30
8.29
8.27
8.27
8.21
8.18
8.05
7.80
7.79
7.78
7.75
7.55
7.52
7.50
7.50
7.48
7.47
7.45
0.62
0.38
0.44
1.26
0.54
3.32
2.23
1.15
2.22
f1 (ppm)

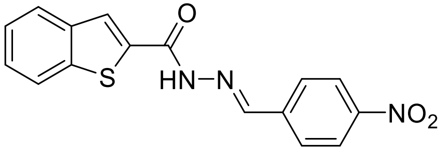
O
S
HN–N
NO2

I.I

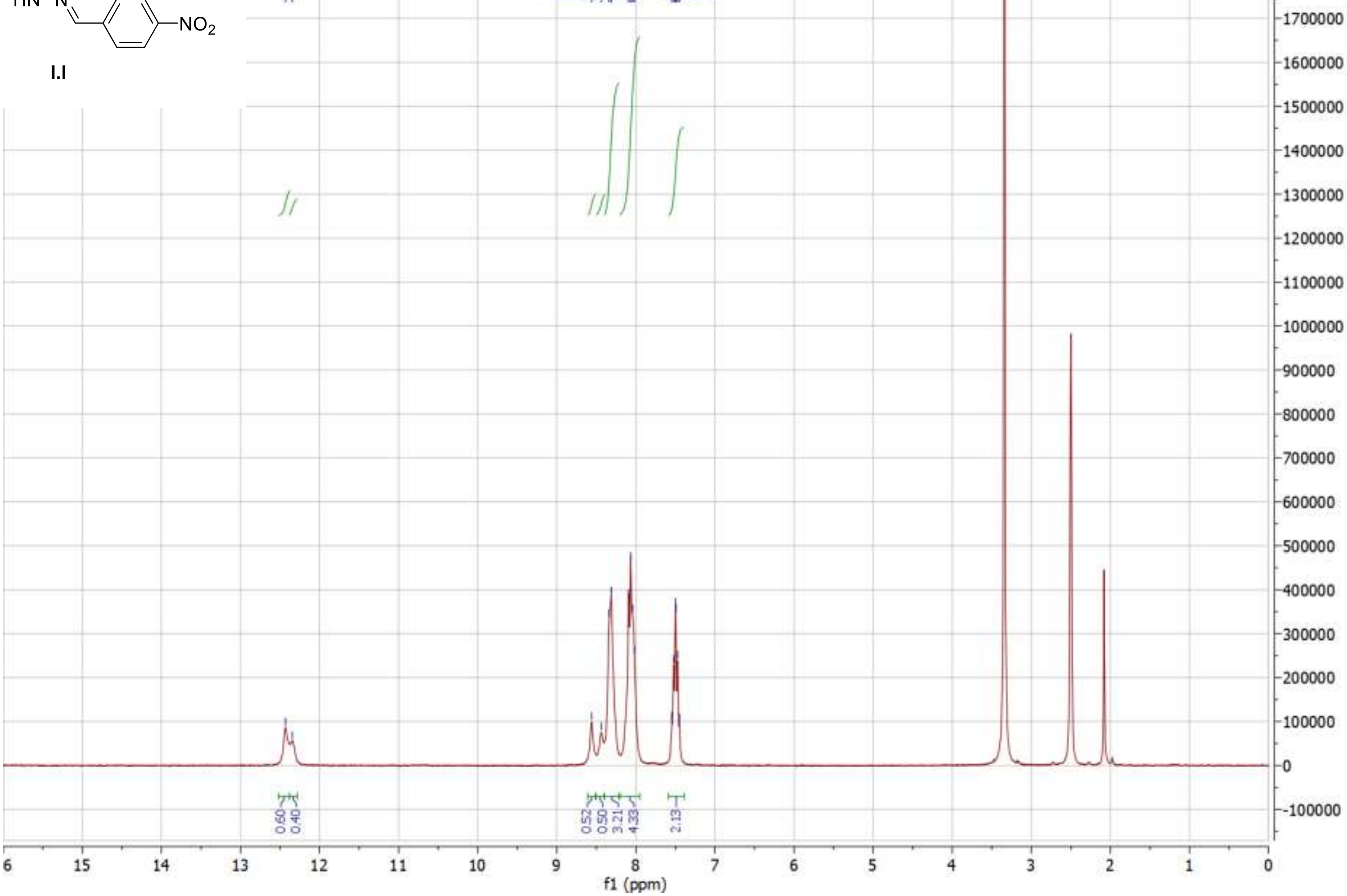
12.43
12.35
8.56
8.44
8.34
8.31
8.31
8.09
8.06
8.04
8.02
7.55
7.52
7.50
7.49
7.47
7.45
0.60
0.40
0.52
0.50
3.21
4.33
2.13
f1 (ppm)

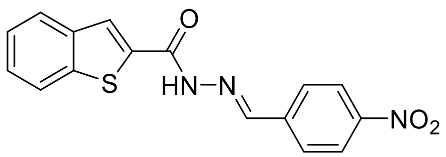
O
HN–N
S
NO_2

I.I

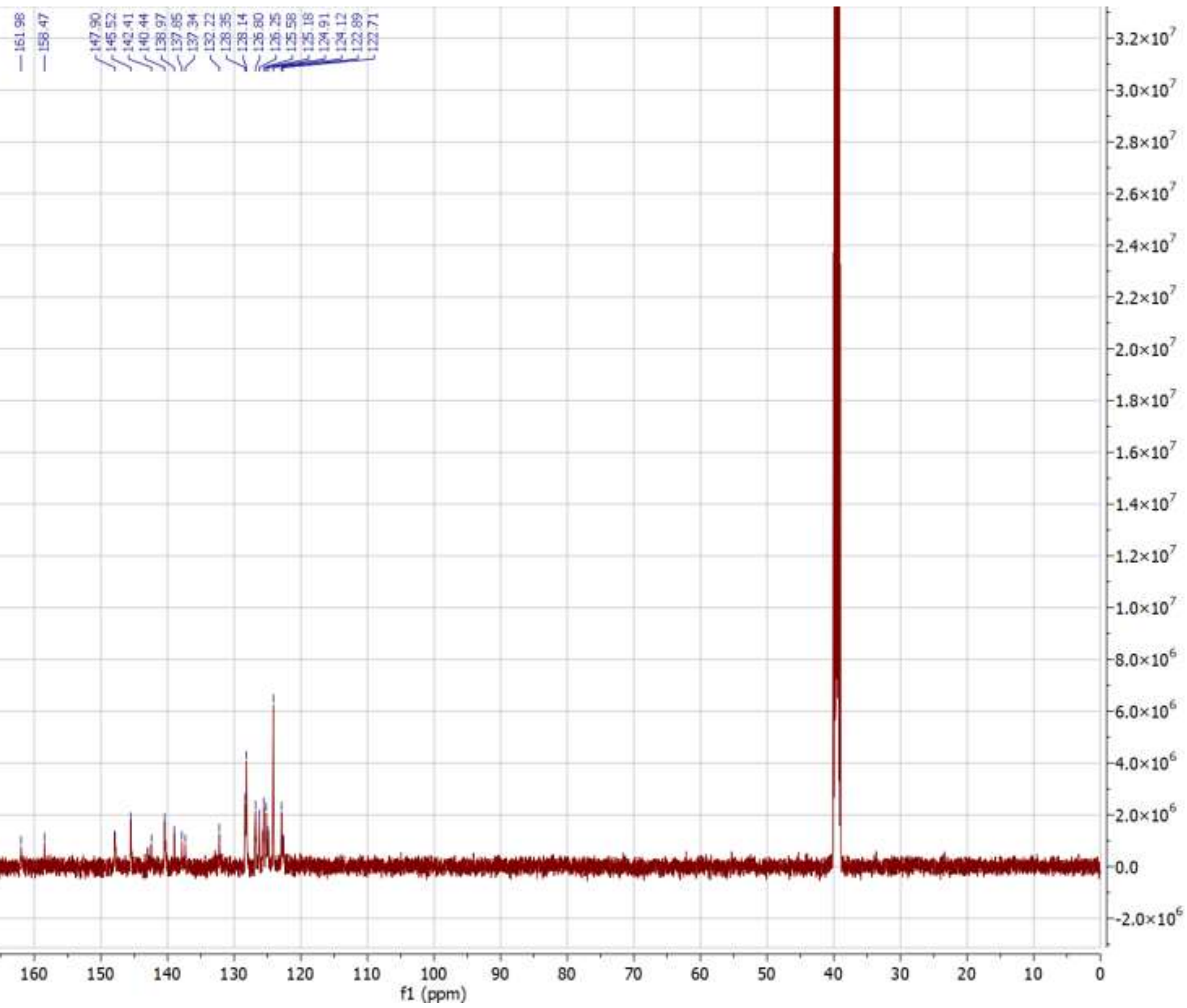
161.98
158.47
147.90
145.52
142.41
140.44
138.97
137.85
137.34
132.22
128.35
128.14
126.80
126.35
125.58
125.18
124.91
124.12
122.89
122.71
3.2×10^7
3.0×10^7
2.8×10^7
2.6×10^7
2.4×10^7
2.2×10^7
2.0×10^7
1.8×10^7
1.6×10^7
1.4×10^7
1.2×10^7
1.0×10^7
8.0×10^6
6.0×10^6
4.0×10^6
2.0×10^6
0.0
-2.0×10^6
190
180
170
160
150
140
130
120
110
100
90
80
70
60
50
40
30
20
10
0
f1 (ppm)

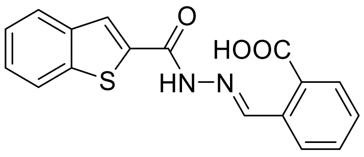

O
HOOC
HN–N
S

I.m

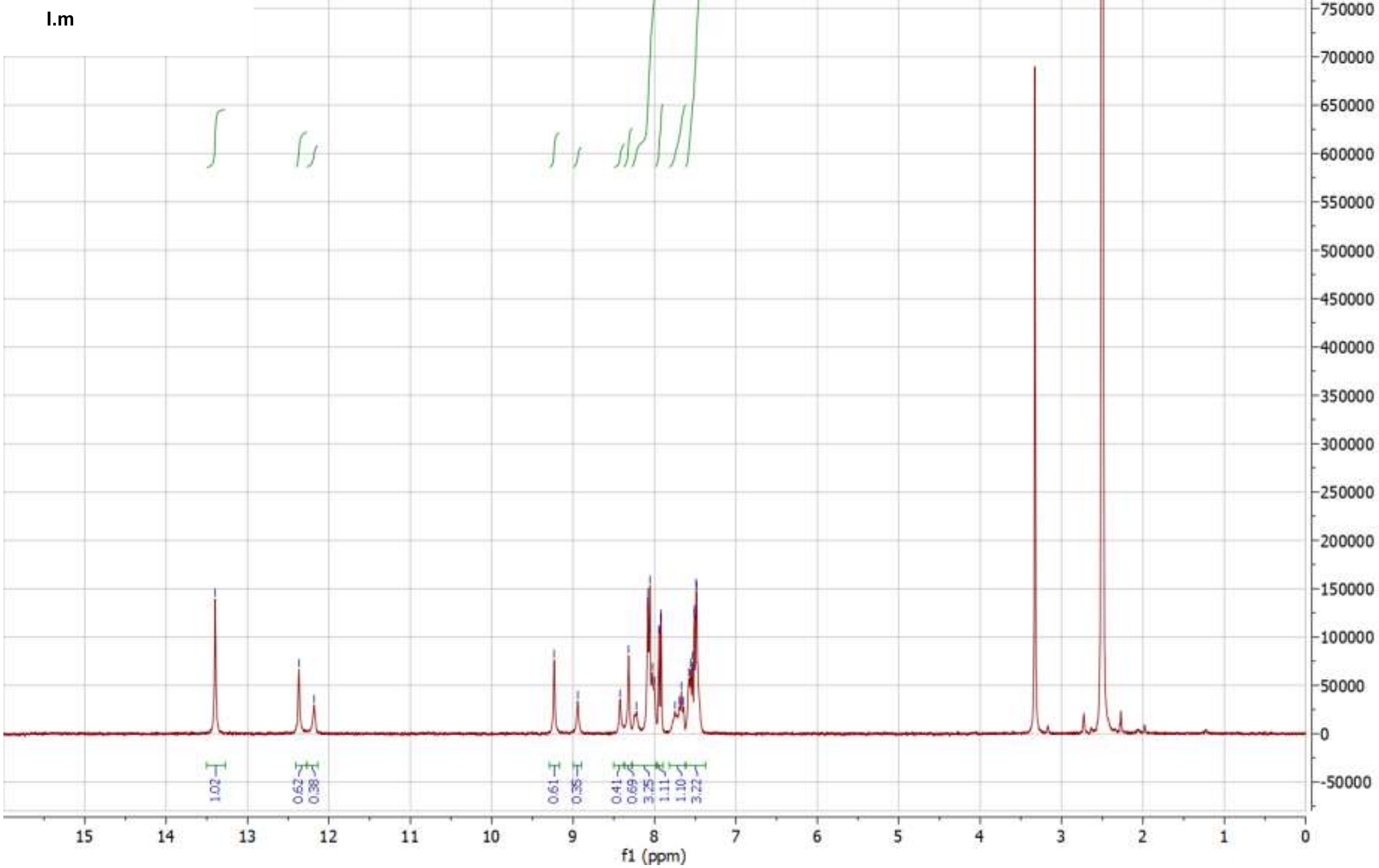

12.37
12.18
9.23
8.94
8.42
8.32
8.22
8.09
8.08
8.06
8.02
8.00
7.95
7.94
7.92
7.92
7.75
7.70
7.67
7.64
7.58
7.56
7.54
7.53
7.51
7.51
7.49
7.48
1.02
0.62
0.38
0.61
0.35
0.41
0.69
3.25
1.11
1.10
3.22
f1 (ppm)

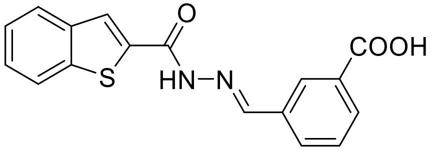
O
COOH
S
HN–N

I.n

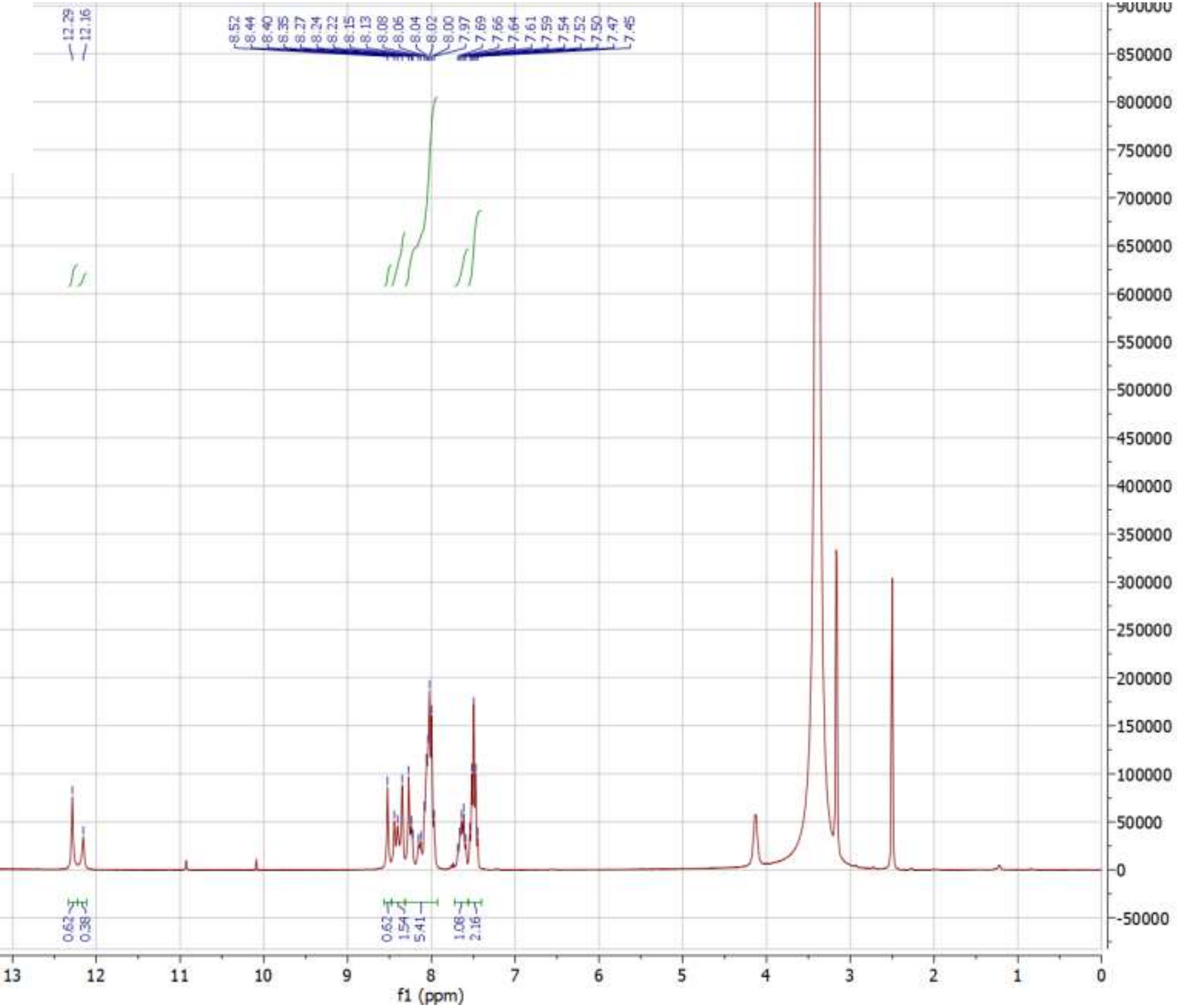
12.29
12.16
8.52
8.44
8.40
8.35
8.27
8.24
8.22
8.15
8.13
8.08
8.06
8.04
8.02
8.00
7.97
7.69
7.66
7.64
7.61
7.59
7.54
7.52
7.50
7.47
7.45
0.62
0.38
0.62
1.54
5.41
1.08
2.16
f1 (ppm)

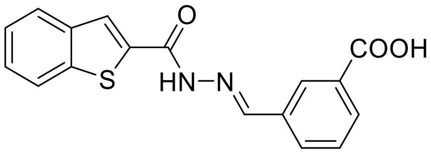
O
COOH
S
HN–N

I.n

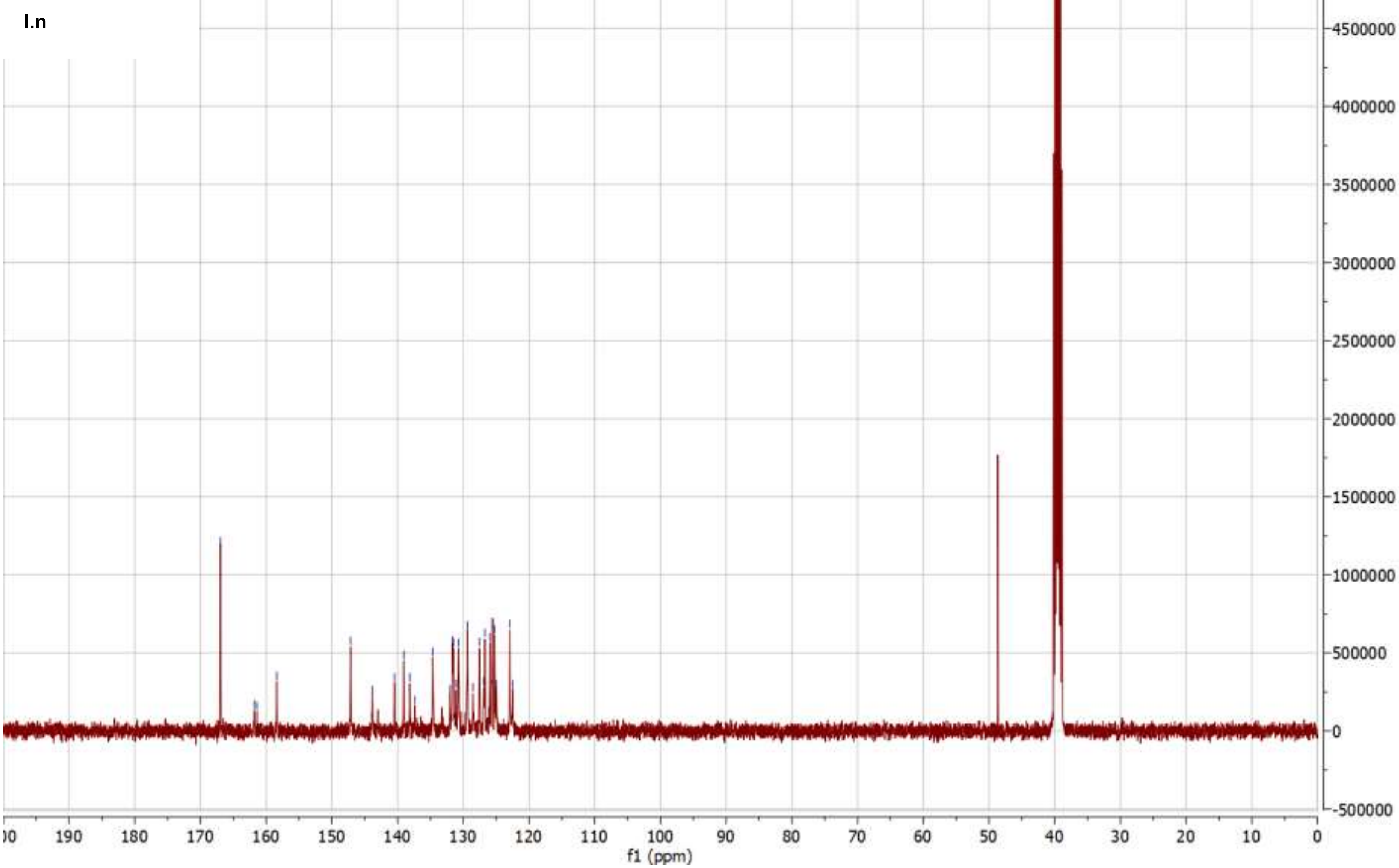
166.97
161.82
161.41
158.41
147.15
143.87
140.46
139.08
138.17
137.40
134.68
132.01
131.65
131.51
131.11
130.78
129.36
128.54
127.54
126.91
126.72
125.97
125.56
125.20
124.96
122.93
122.49
f1 (ppm)

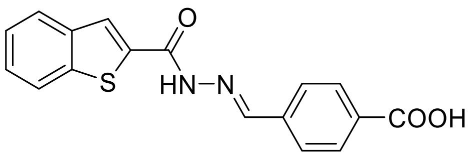
O
S
HN–N
COOH

I.o

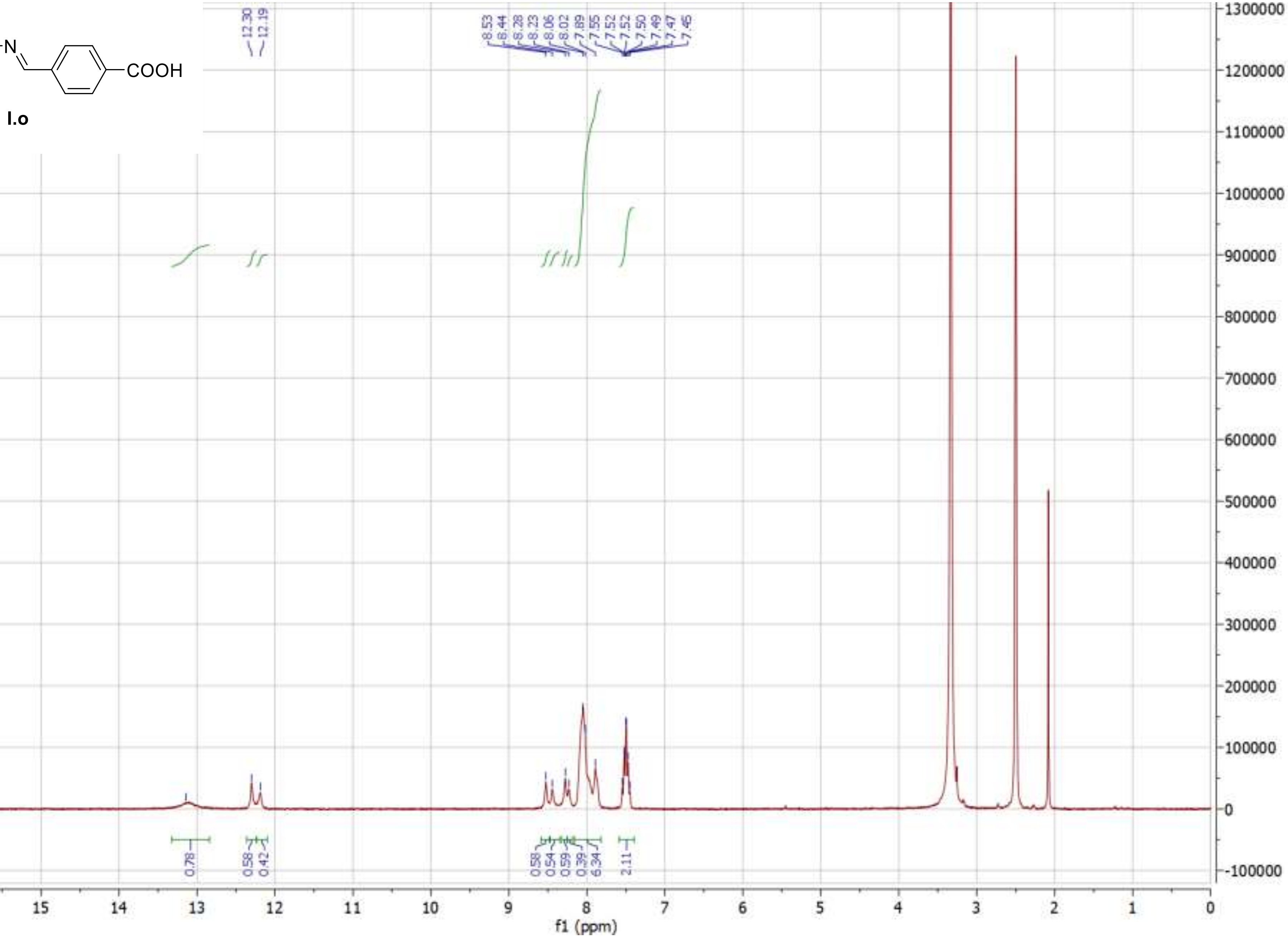
12.30
12.19
8.53
8.44
8.28
8.23
8.06
8.02
7.89
7.55
7.52
7.52
7.50
7.49
7.47
7.45
0.78
0.58
0.42
0.58
0.54
0.59
0.39
6.34
2.11
1300000
1200000
1100000
1000000
900000
800000
700000
600000
500000
400000
300000
200000
100000
0
-100000
16 15 14 13 12 11 10 9 8 7 6 5 4 3 2 1 0
f1 (ppm)

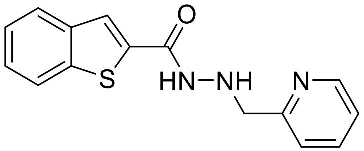

O
S
HN–NH
N

I.p

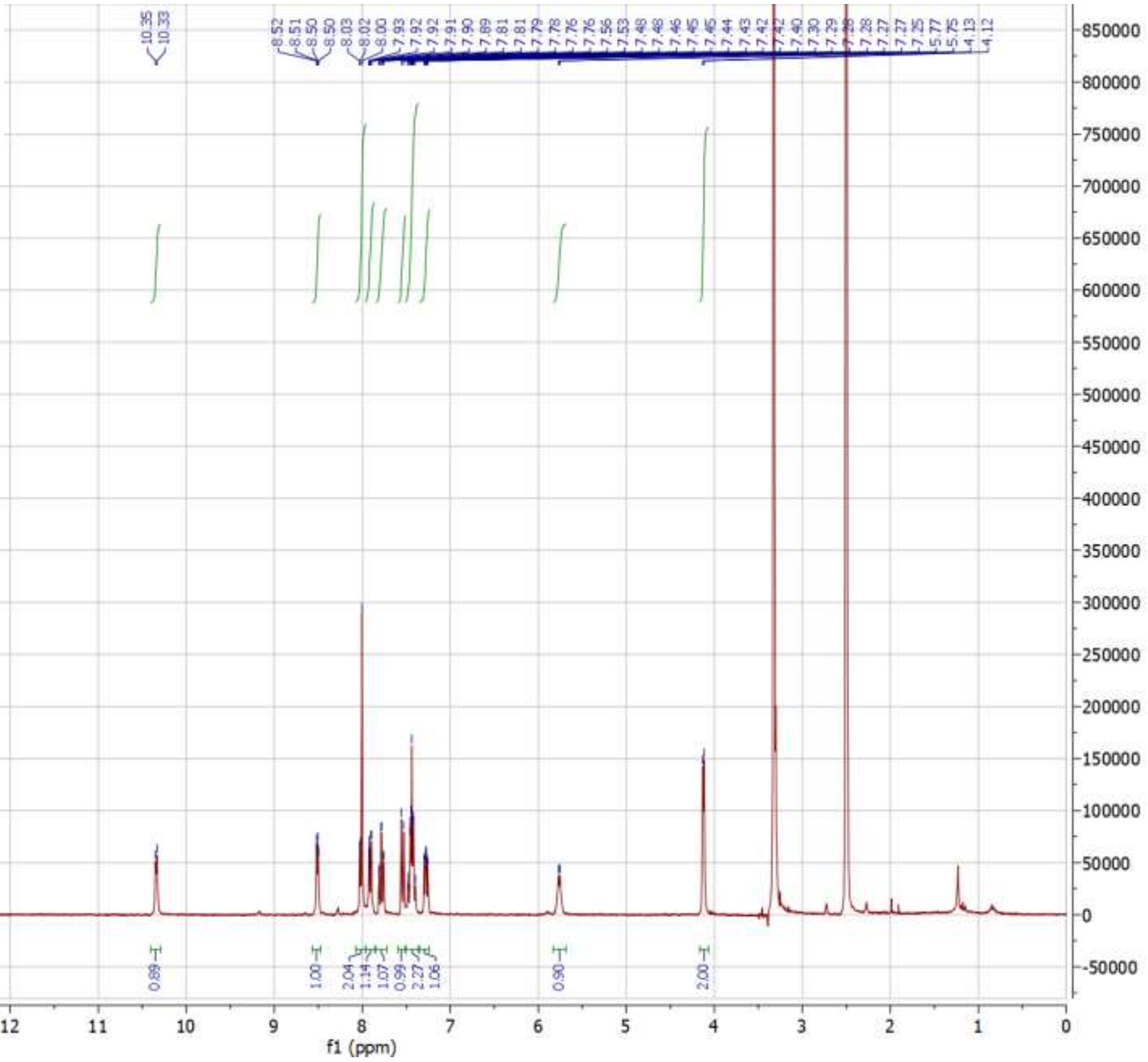

10.35
10.33
8.52
8.51
8.50
8.50
8.03
8.02
8.00
7.93
7.92
7.92
7.91
7.90
7.89
7.81
7.79
7.78
7.76
7.76
7.56
7.53
7.48
7.48
7.46
7.45
7.45
7.44
7.43
7.42
7.42
7.40
7.30
7.29
7.28
7.28
7.27
7.27
7.25
5.77
5.75
4.13
4.12
0.89
1.00
2.04
1.14
1.07
0.99
2.27
1.06
0.90
2.00
f1 (ppm)

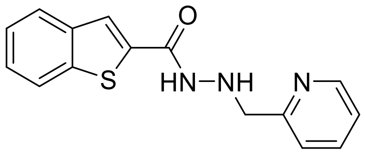
O
S
HN–NH
N

I.p

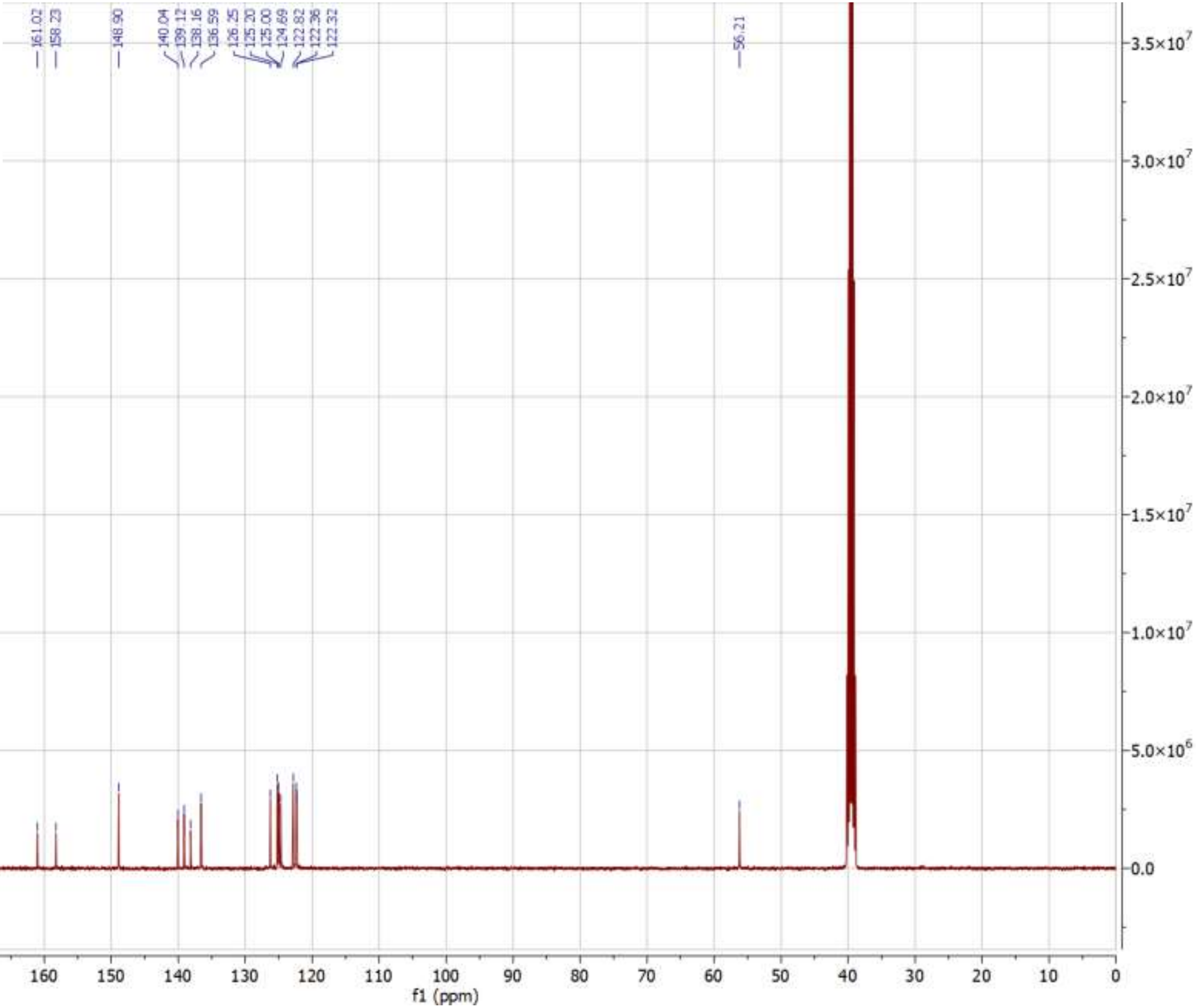
161.02
158.23
148.90
140.04
139.12
138.16
136.59
126.25
125.20
125.00
124.69
122.82
122.36
122.32
56.21
3.5×10^7
3.0×10^7
2.5×10^7
2.0×10^7
1.5×10^7
1.0×10^7
5.0×10^6
0.0
00 190 180 170 160 150 140 130 120 110 100 90 80 70 60 50 40 30 20 10 0
f1 (ppm)

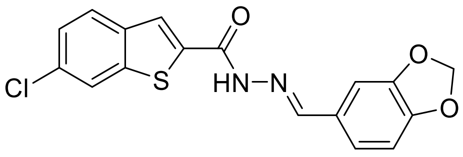

II.a

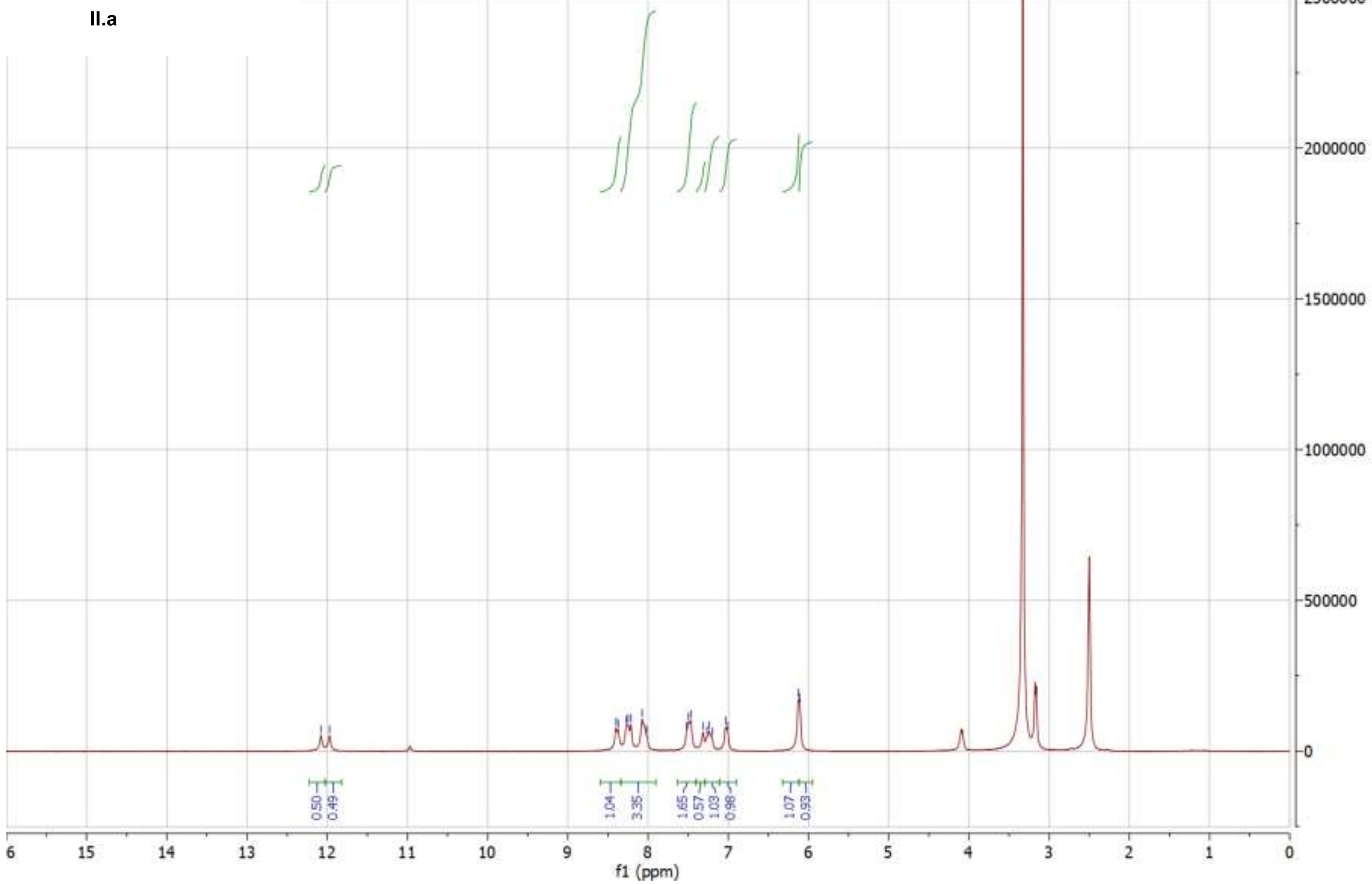

12.08
11.97
8.40
8.37
8.27
8.25
8.21
8.07
8.02
7.52
7.50
7.47
7.31
7.27
7.24
7.20
7.03
7.01
6.13
6.11
2500000
2000000
1500000
1000000
500000
0
0.50
0.49
1.04
3.35
1.65
0.57
1.03
0.98
1.07
0.93
f1 (ppm)

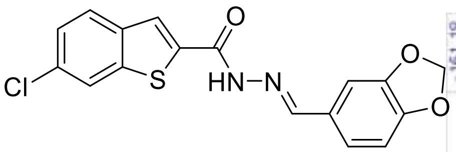
Cl
S
O
HN–N
O
O

II.a

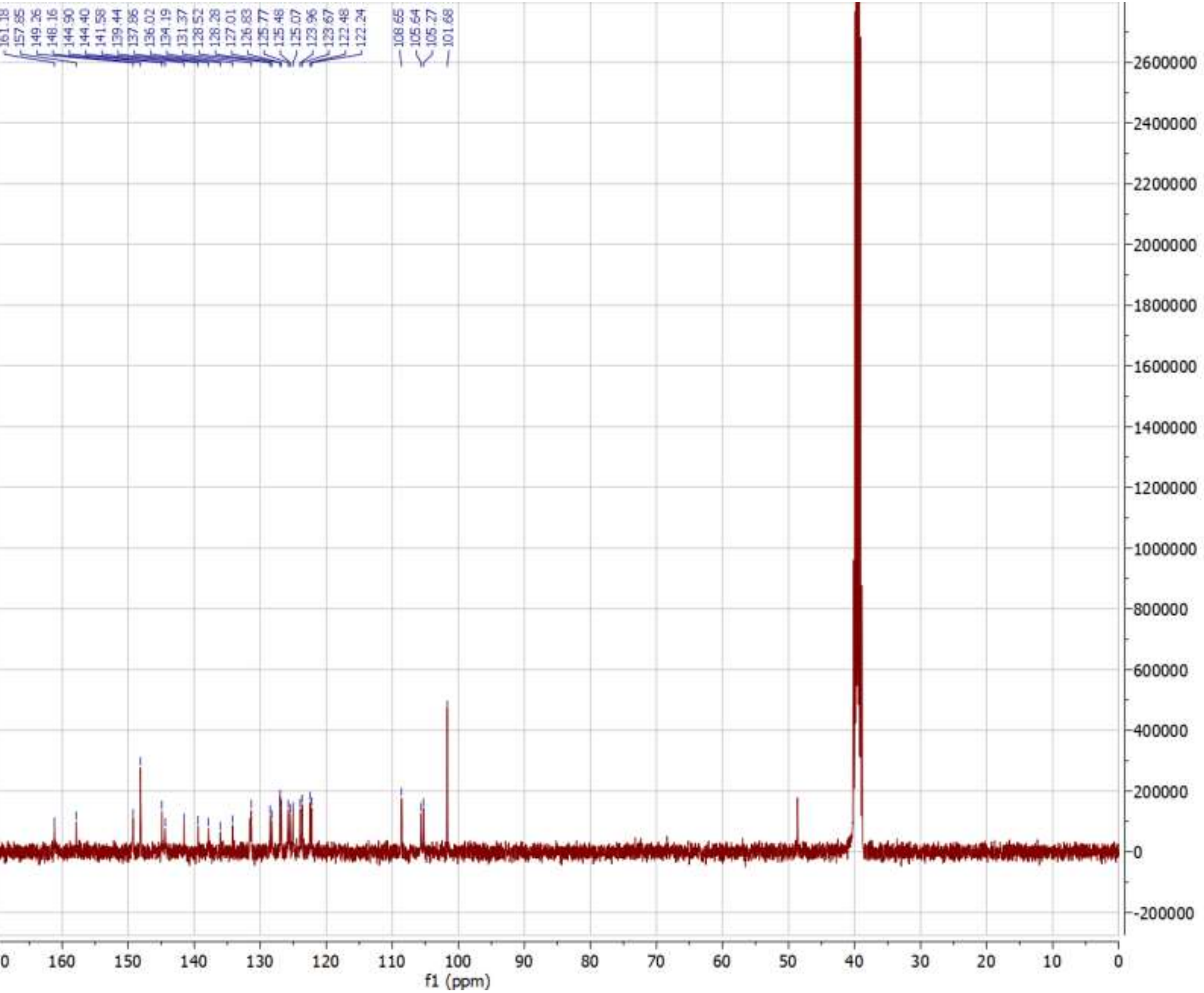
161.18
157.85
149.26
148.16
144.90
144.40
141.58
139.44
137.86
136.02
134.19
131.37
128.52
128.28
127.01
126.83
125.77
125.48
125.07
123.96
123.67
122.48
122.24
108.65
105.64
105.27
101.68
f1 (ppm)

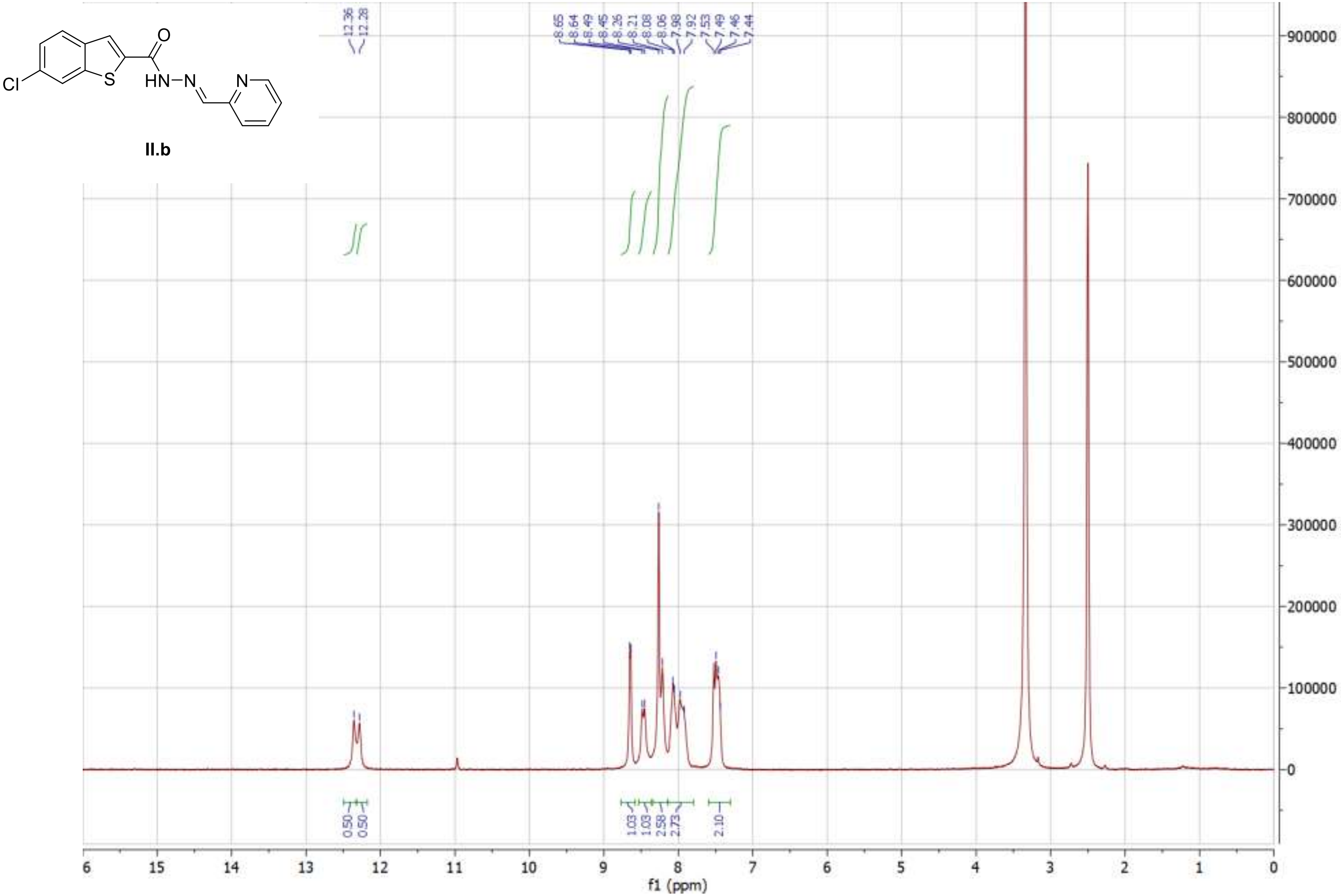

O
Cl
S
HN
N
N
12.36
12.28
8.65
8.64
8.49
8.45
8.26
8.21
8.08
8.06
7.98
7.92
7.53
7.49
7.46
7.44
0.50
0.50
1.03
1.03
2.58
2.73
2.10
900000
800000
700000
600000
500000
400000
300000
200000
100000
0
6
15
14
13
12
11
10
9
8
7
6
5
4
3
2
1
0
f1 (ppm)

II.b

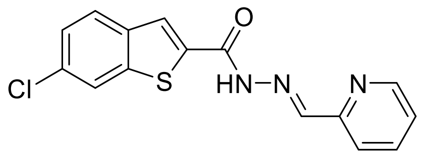

II.b

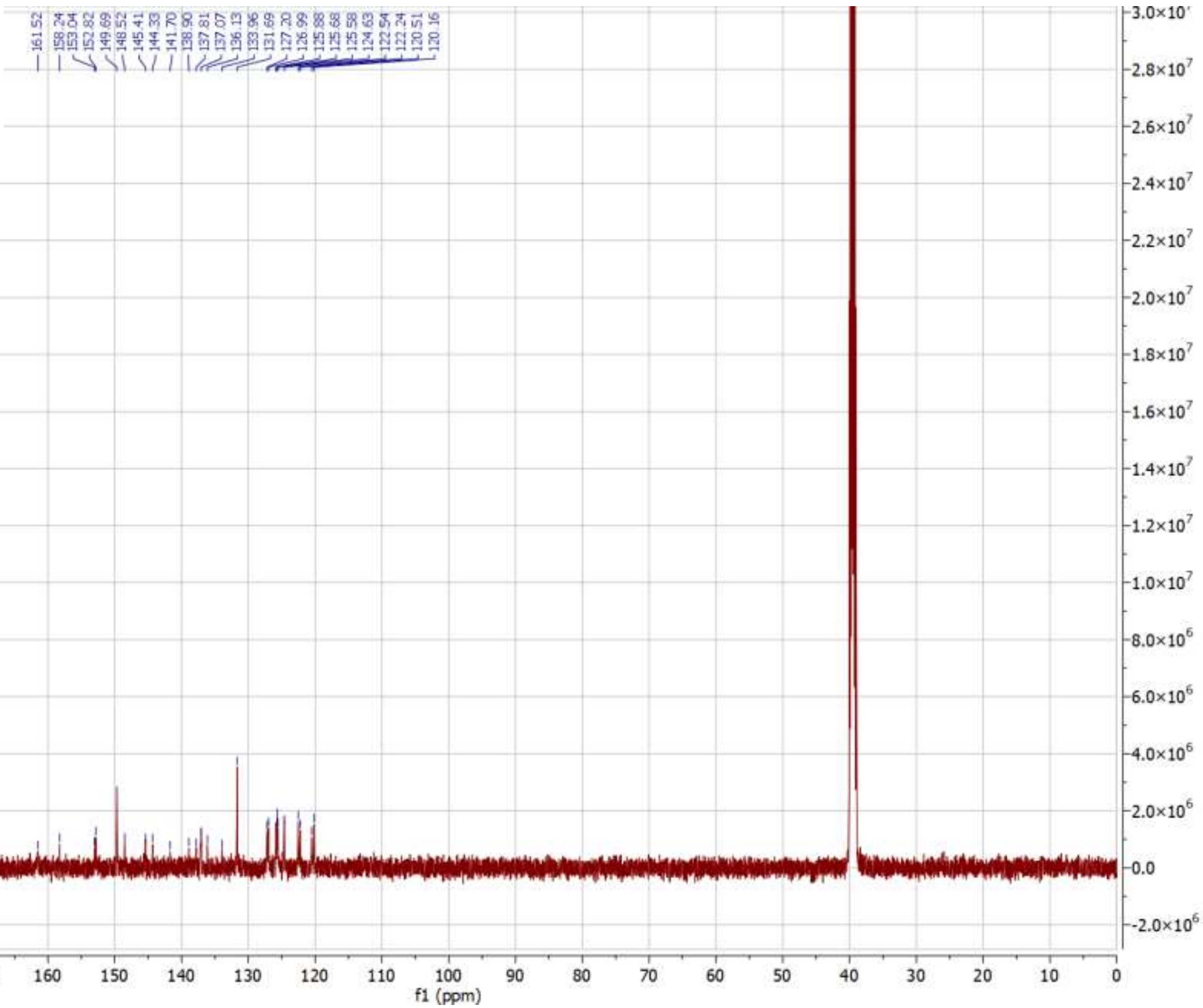
161.52
158.24
153.04
152.82
149.69
148.52
145.41
144.33
141.70
138.90
137.81
137.07
136.13
133.96
131.69
127.20
126.99
125.88
125.68
125.58
124.63
122.54
122.24
120.51
120.16
f1 (ppm)

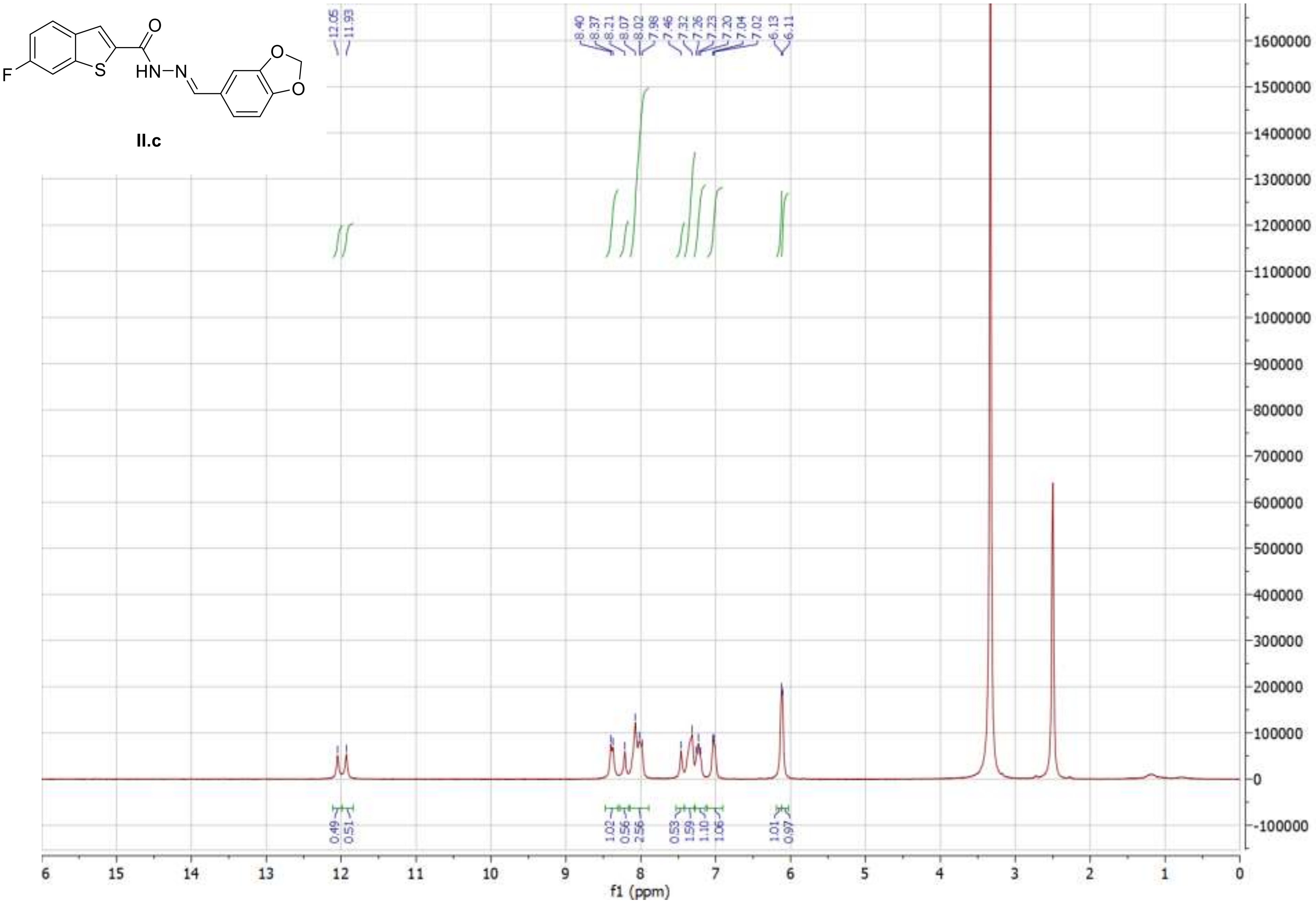
O
F
S
HN
N
O
O
12.05
11.93
8.40
8.37
8.21
8.07
8.02
7.98
7.46
7.32
7.26
7.23
7.20
7.04
7.02
6.13
6.11
0.49
0.51
1.02
0.56
2.56
0.53
1.59
1.10
1.06
1.01
0.97
f1 (ppm)

II.c

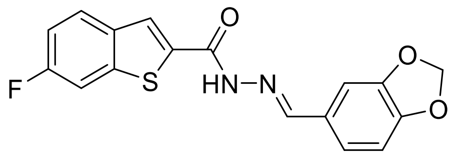
F
S
O
HN
N
O
O

II.c

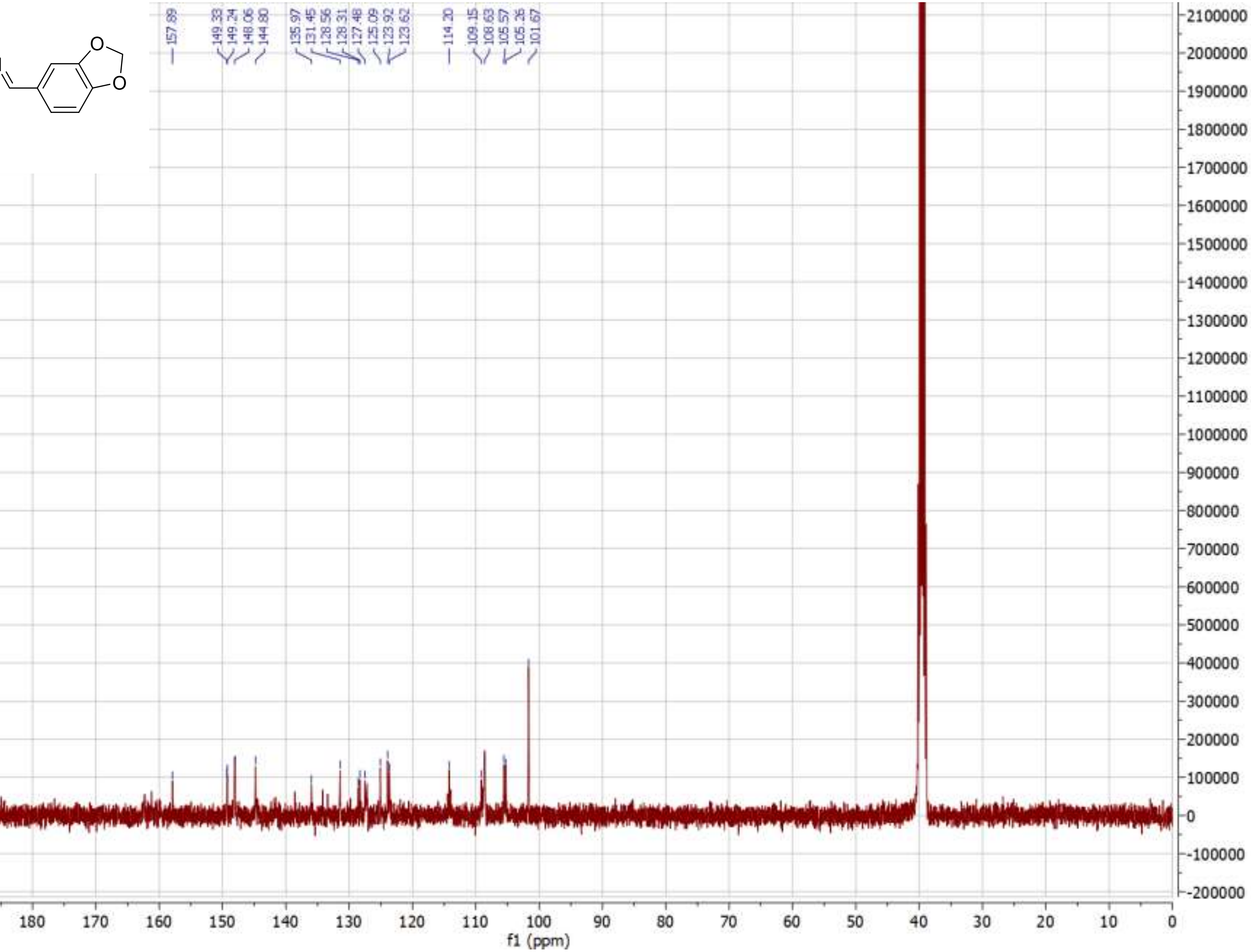
157.89
149.33
149.24
148.06
144.80
135.97
131.45
128.56
128.31
127.48
125.09
123.92
123.62
114.20
109.15
108.63
105.57
105.36
101.67
f1 (ppm)

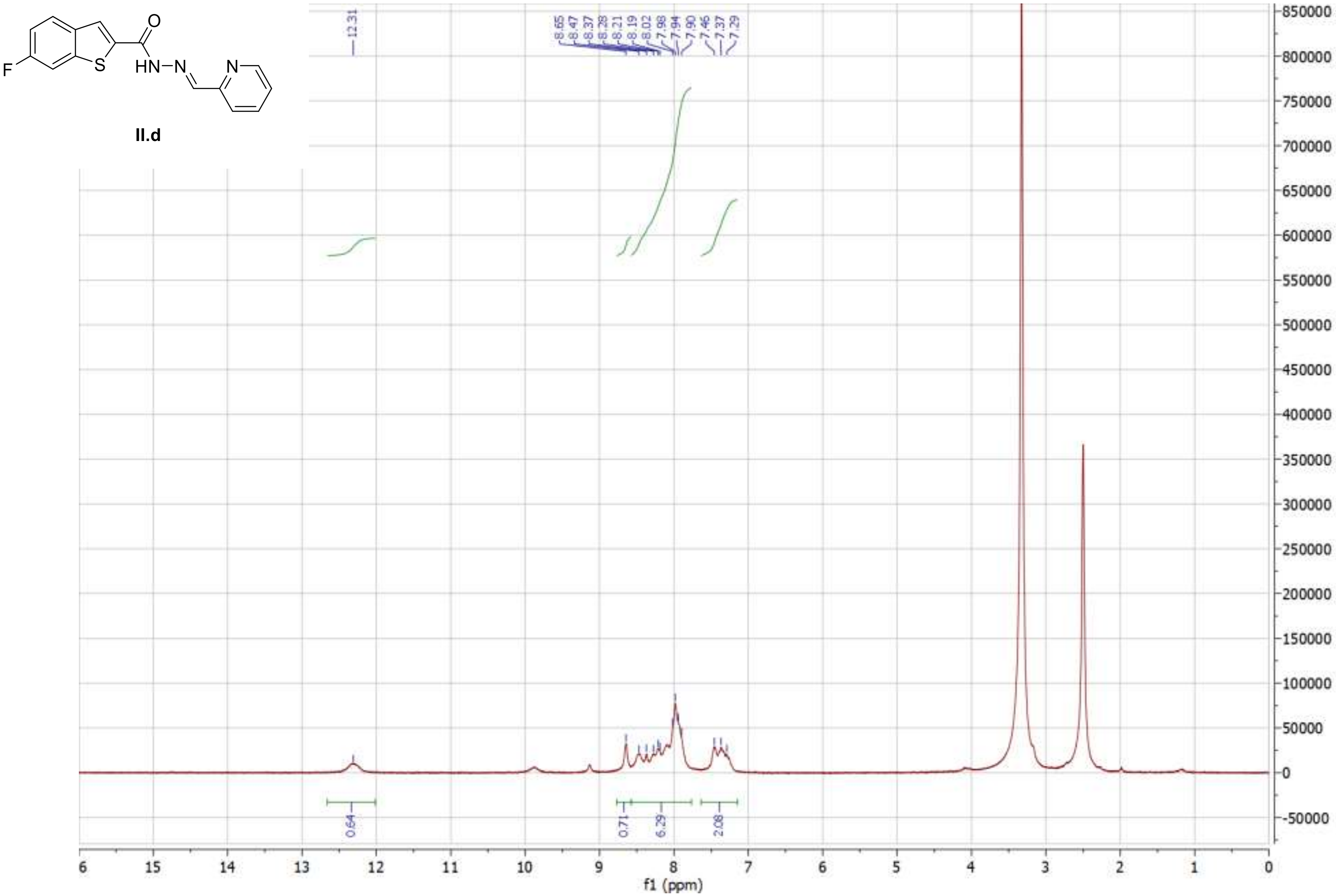
O
F
S
HN–N
N
II.d
12.31
8.65
8.47
8.37
8.28
8.21
8.19
8.02
7.98
7.94
7.90
7.46
7.37
7.29
0.64
0.71
6.29
2.08
f1 (ppm)

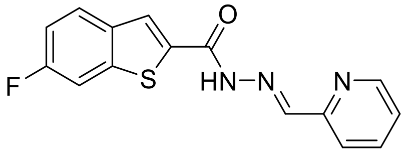

II.d

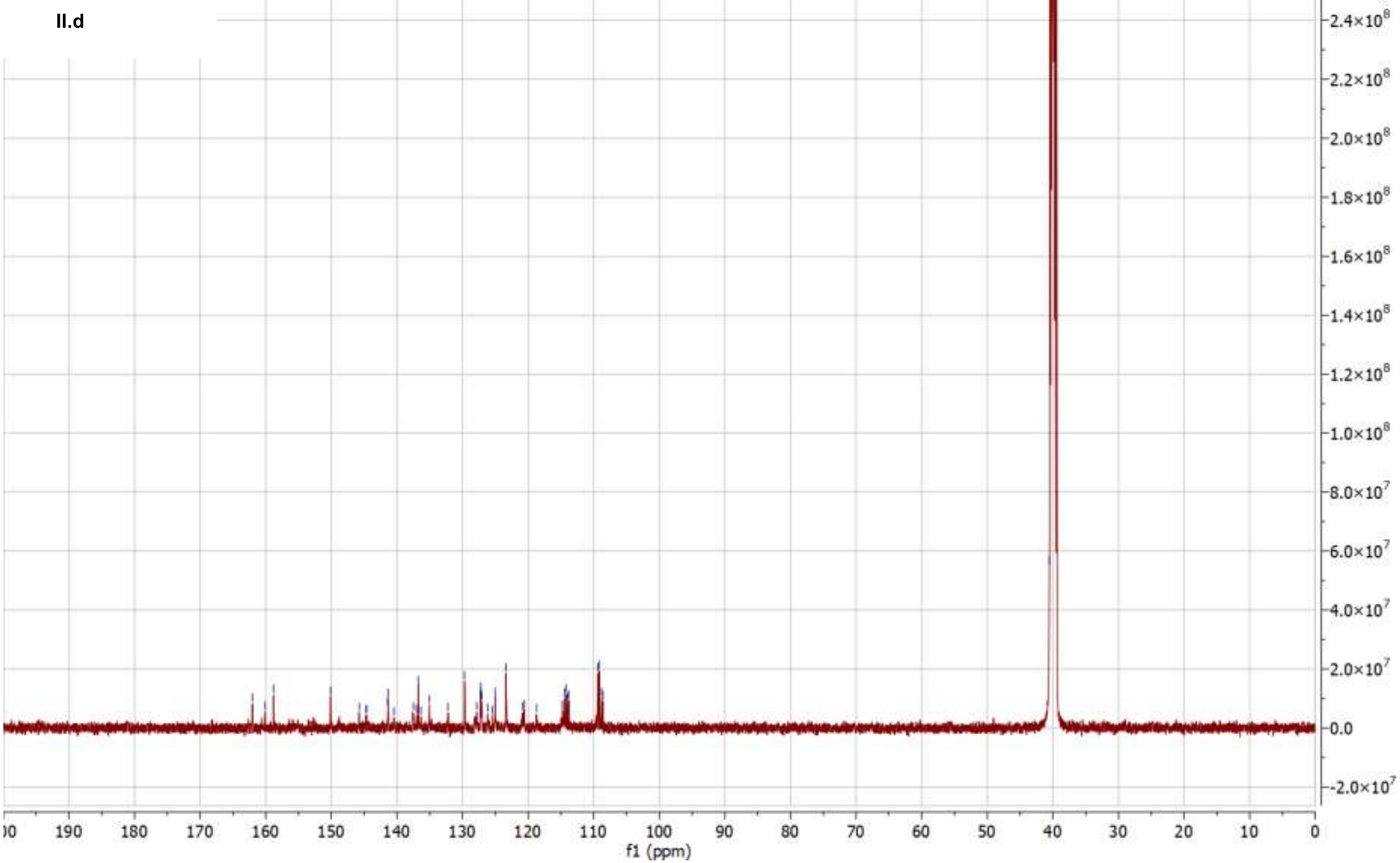

162.02
160.08
158.80
150.13
145.75
144.71
144.62
141.43
141.34
140.47
137.50
137.04
136.76
136.33
135.04
132.22
129.70
127.83
127.28
127.20
127.15
127.07
126.16
125.48
125.03
123.37
120.85
120.60
118.72
114.78
114.43
114.24
114.01
113.82
109.37
109.16
108.79
108.59
40.54
f1 (ppm)

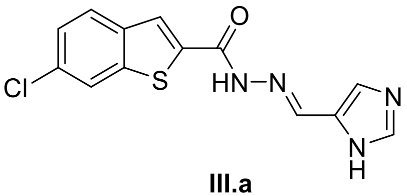
Cl
S
O
HN–N
N
N
H

III.a

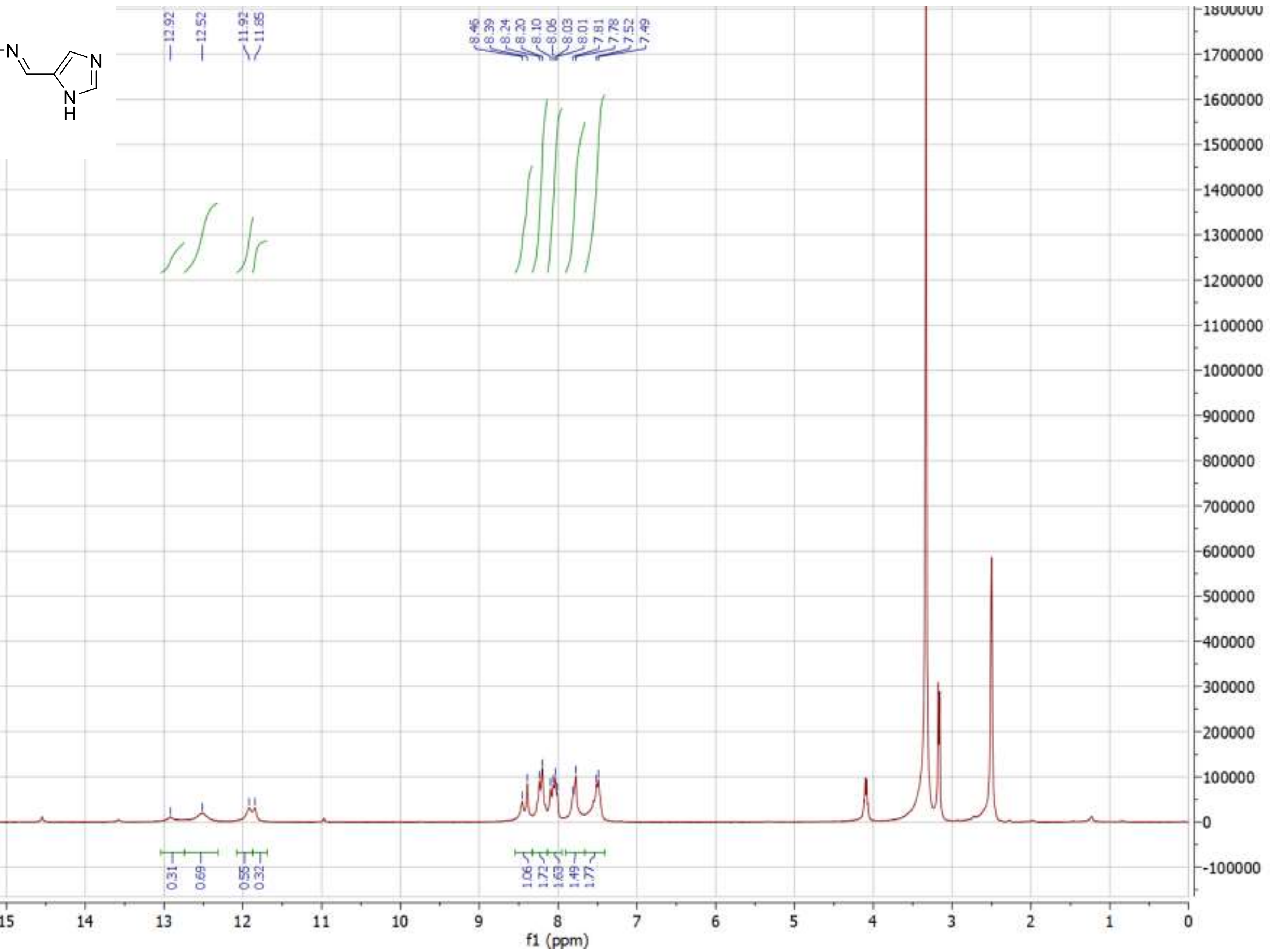
12.92
12.52
11.92
11.85
8.46
8.39
8.24
8.20
8.10
8.06
8.03
8.01
7.81
7.78
7.52
7.49
0.31
0.69
0.55
0.32
1.06
1.72
1.63
1.49
1.77
f1 (ppm)

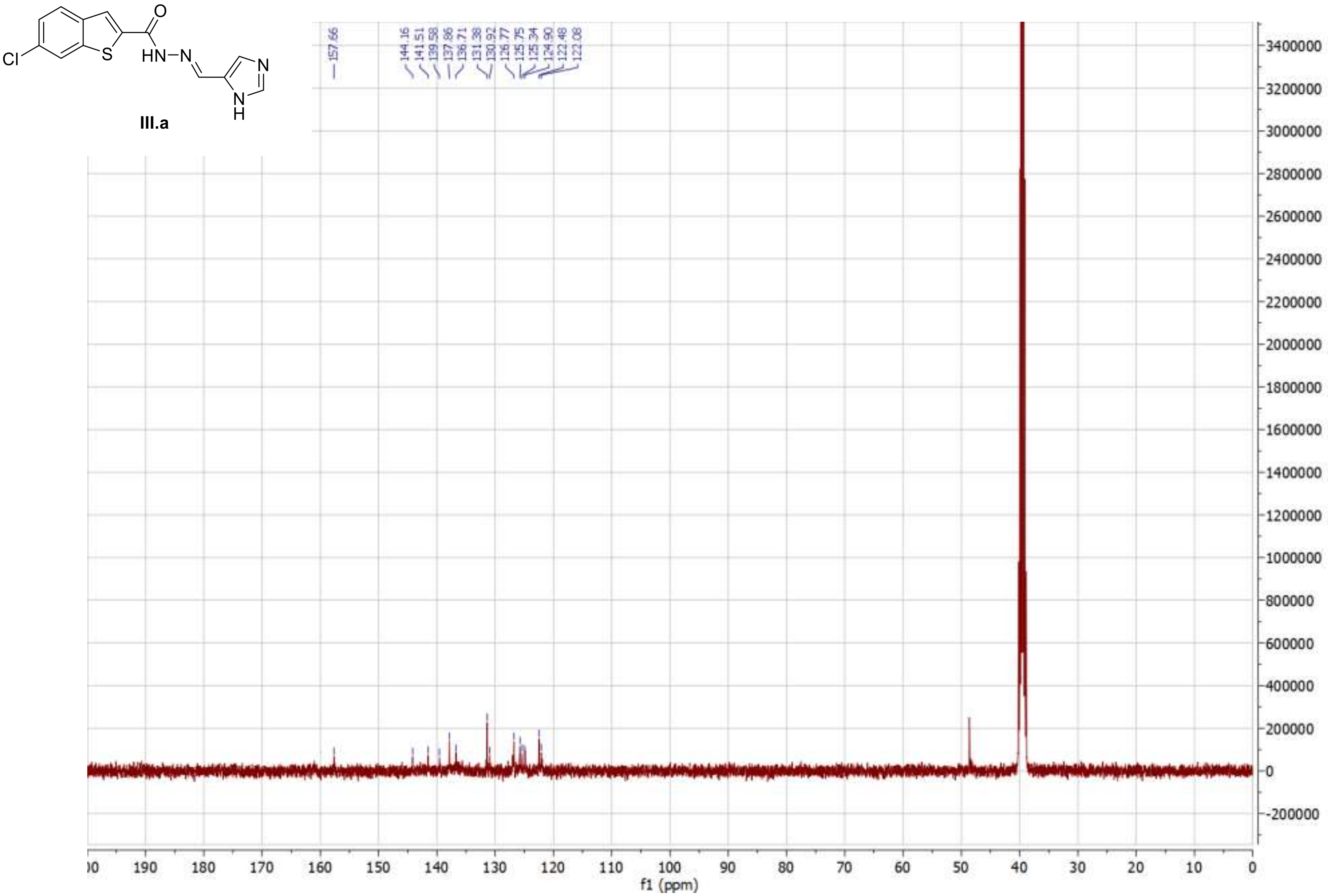
O
Cl
S
HN–N
N
N
H
III.a
157.66
144.16
141.51
139.58
137.86
136.71
131.38
130.92
126.77
125.75
125.34
124.90
122.48
122.08
f1 (ppm)

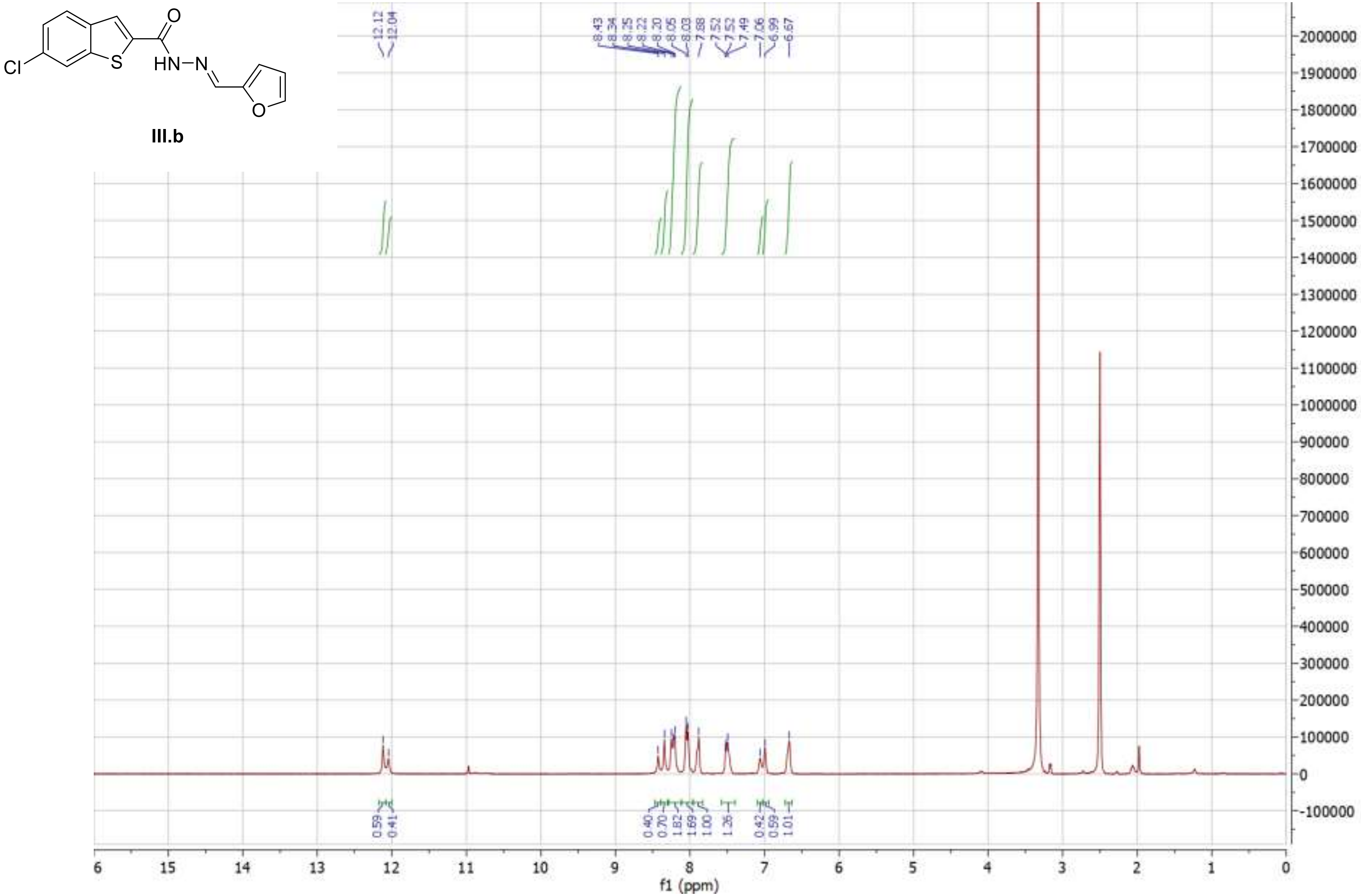

III.b

12.12
12.04
8.43
8.34
8.25
8.22
8.20
8.05
8.03
7.88
7.52
7.52
7.49
7.06
6.99
6.67
0.59
0.41
0.40
0.70
1.82
1.69
1.00
1.26
0.42
0.59
1.01
f1 (ppm)

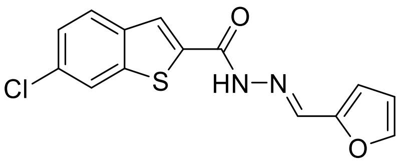
O
Cl
S
HN–N
O

III.b

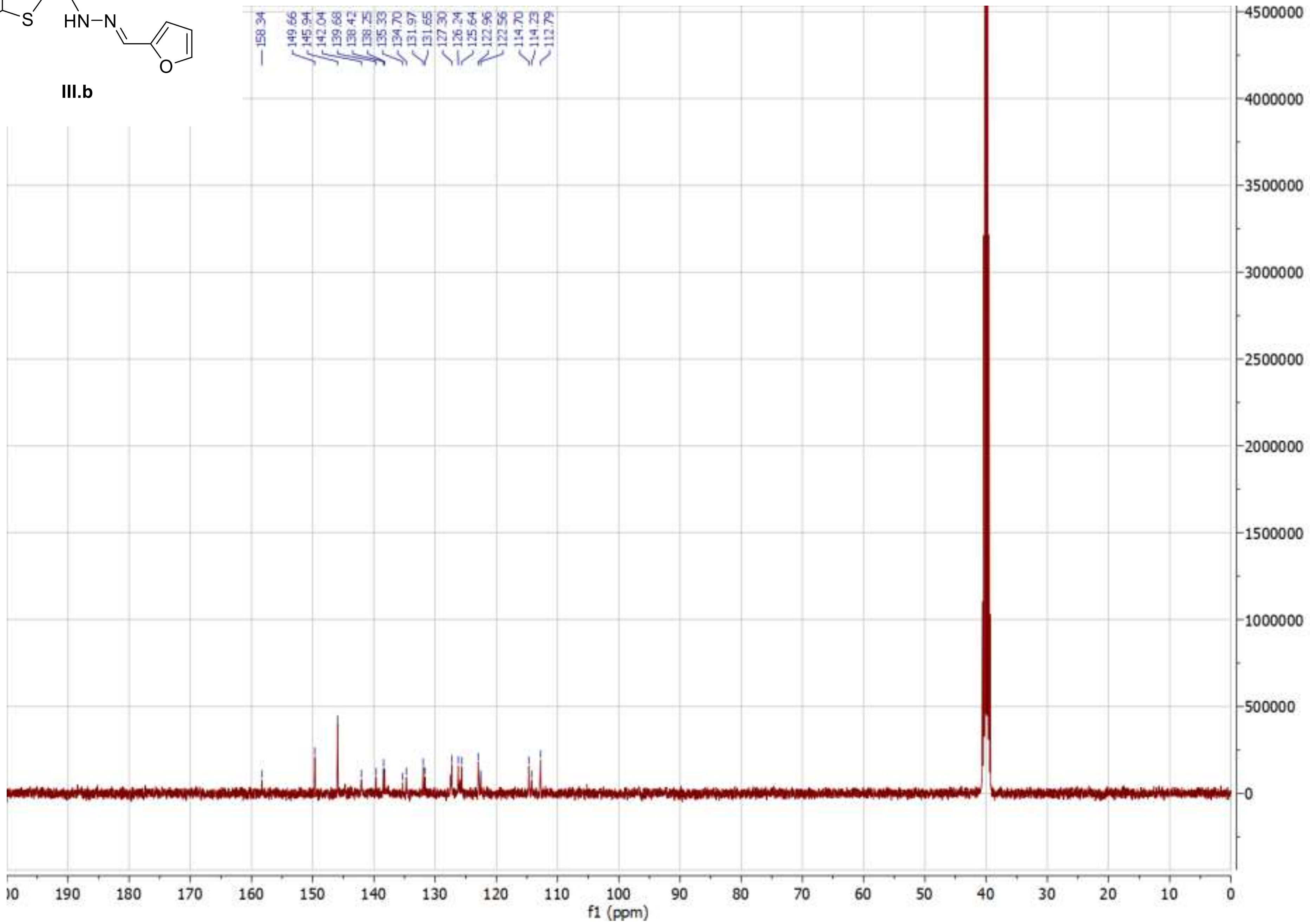
158.34
149.66
145.94
142.04
139.68
138.42
138.25
135.33
134.70
131.97
131.65
127.30
126.24
125.64
122.96
122.56
114.70
114.23
112.79
4500000
4000000
3500000
3000000
2500000
2000000
1500000
1000000
500000
0
00
190
180
170
160
150
140
130
120
110
100
90
80
70
60
50
40
30
20
10
0
f1 (ppm)

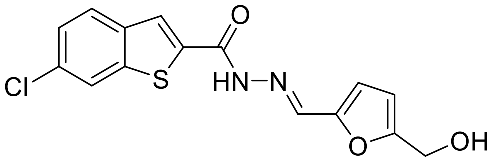
Cl
S
O
HN
N
O
OH

III.c

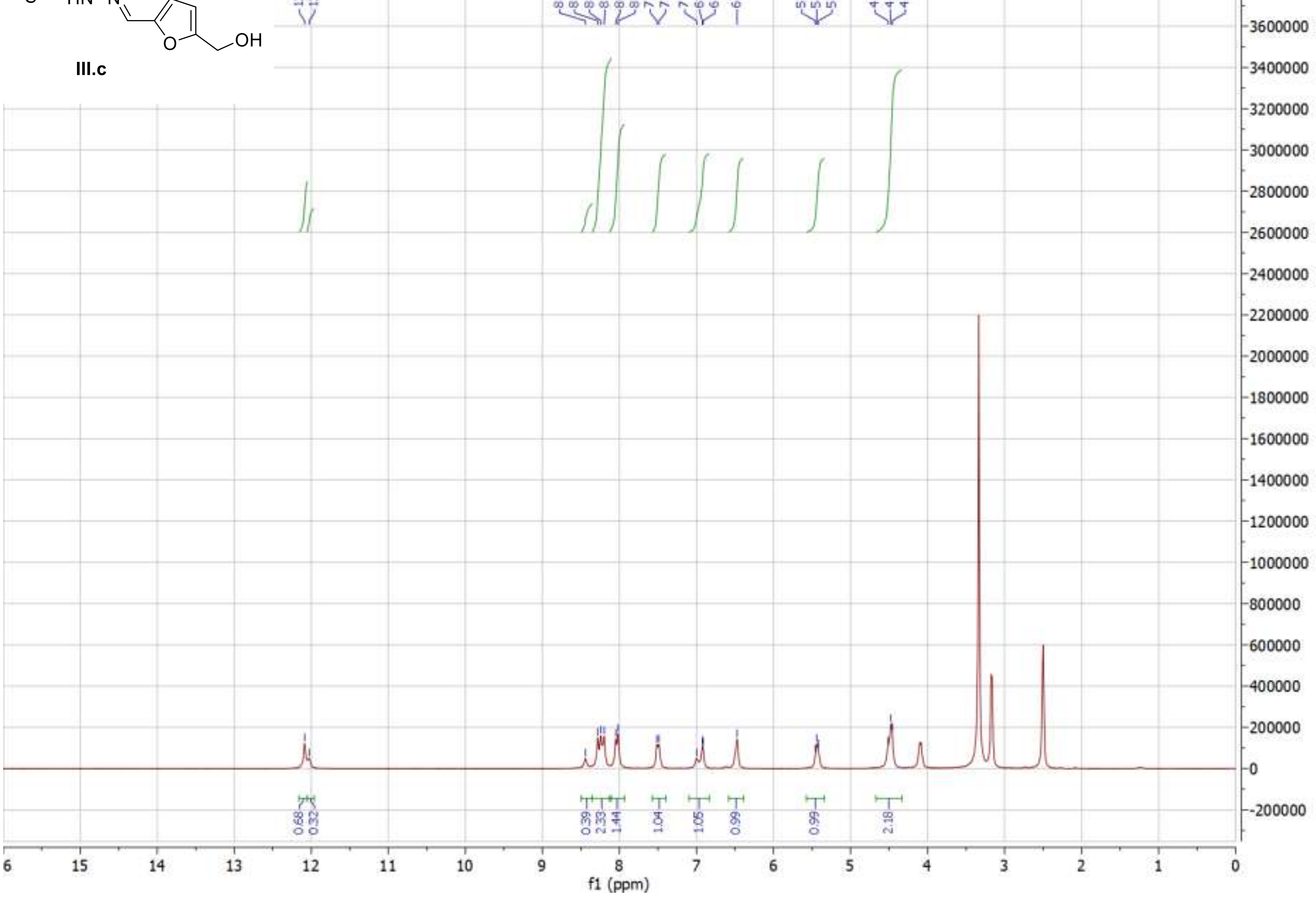
12.09
12.03
8.44
8.28
8.24
8.20
8.05
8.02
7.51
7.49
7.00
6.92
6.91
6.47
5.46
5.44
5.42
4.51
4.48
4.46
0.68
0.32
0.39
2.33
1.44
1.04
1.05
0.99
0.99
2.18
f1 (ppm)

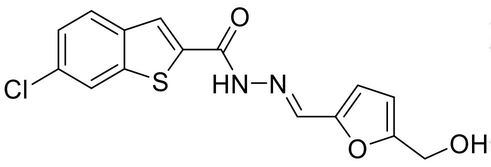
O
Cl
S
HN–N
O
OH

III.c

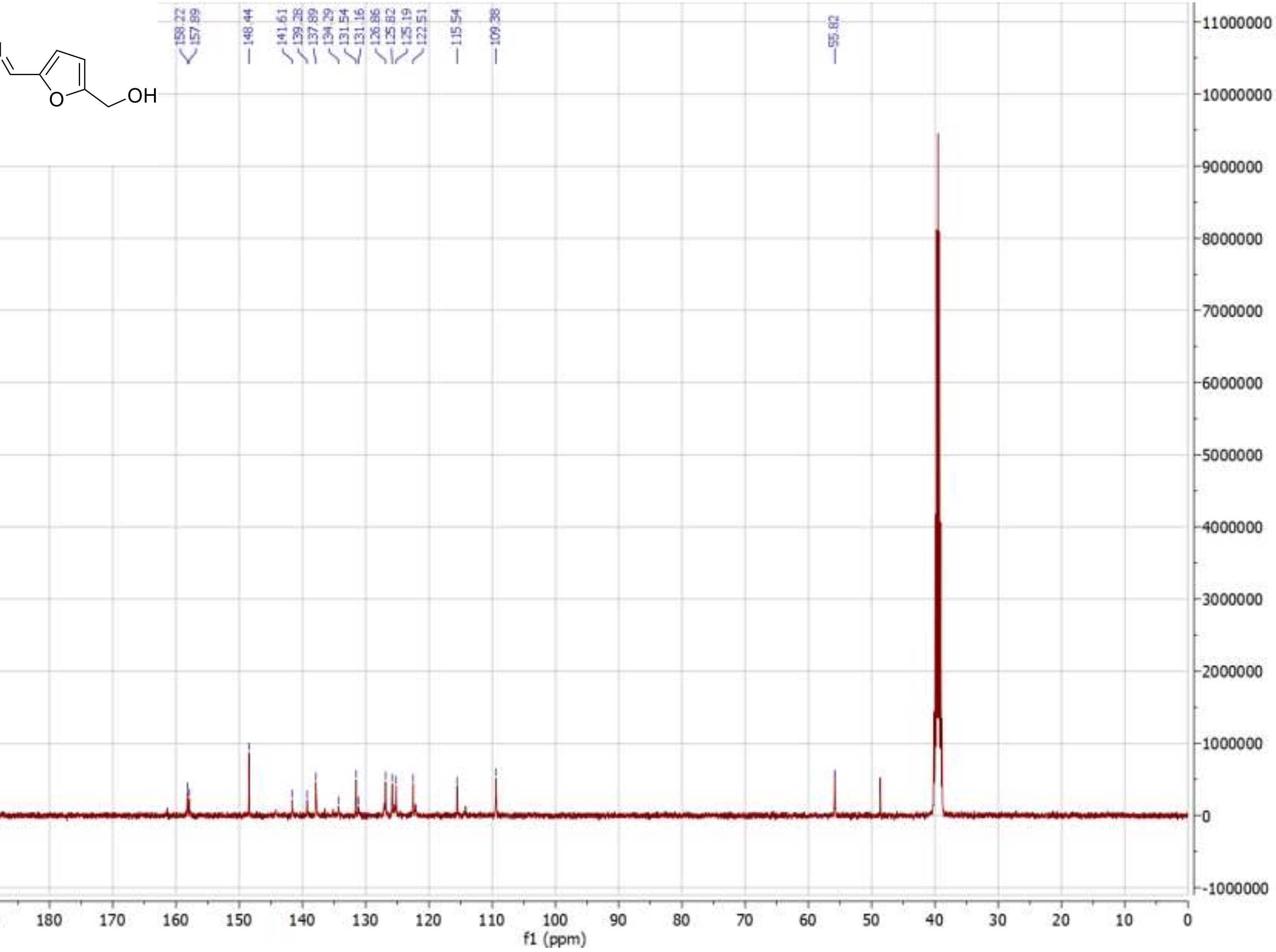
158.22
157.89
148.44
141.61
139.28
137.89
134.29
131.54
131.16
126.86
125.82
125.19
122.51
115.54
109.38
55.82
f1 (ppm)

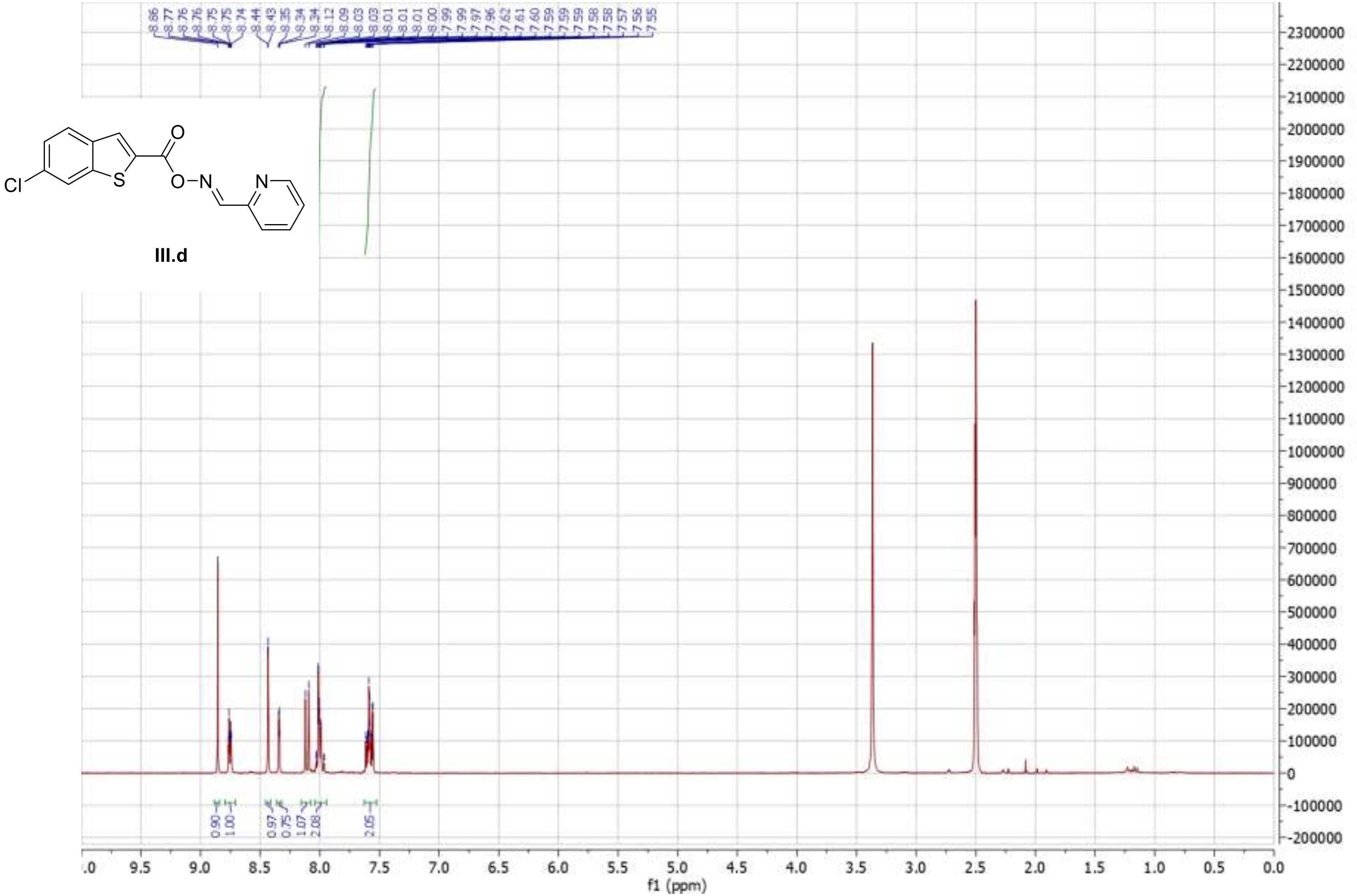

8.86
8.77
8.76
8.76
8.75
8.75
8.74
8.44
8.43
8.35
8.34
8.34
8.12
8.09
8.03
8.03
8.01
8.01
8.01
8.00
7.99
7.99
7.97
7.96
7.62
7.61
7.60
7.59
7.59
7.59
7.58
7.58
7.57
7.56
7.55
O
Cl
S
O
N
N
III.d
0.90
1.00
0.97
0.75
1.07
2.08
2.05
f1 (ppm)

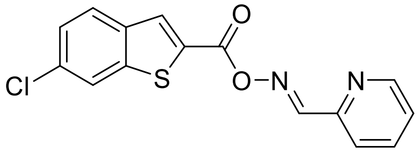
Cl
S
O
O
N
N

III.d

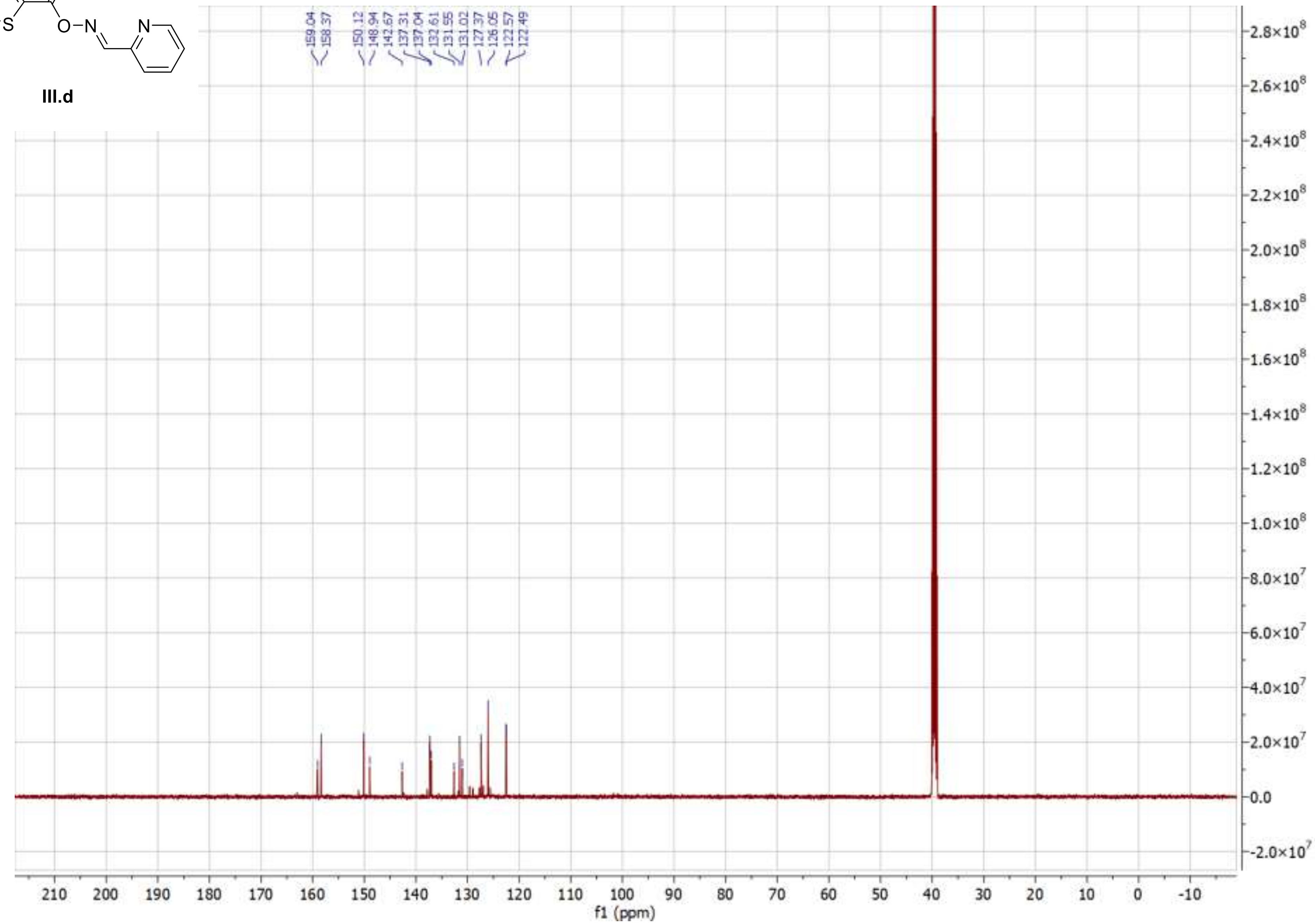
159.04
158.37
150.12
148.94
142.67
137.31
137.04
132.61
131.55
131.02
127.37
126.05
122.57
122.49
2.8×10^8
2.6×10^8
2.4×10^8
2.2×10^8
2.0×10^8
1.8×10^8
1.6×10^8
1.4×10^8
1.2×10^8
1.0×10^8
8.0×10^7
6.0×10^7
4.0×10^7
2.0×10^7
0.0
-2.0×10^7
210 200 190 180 170 160 150 140 130 120 110 100 90 80 70 60 50 40 30 20 10 0 -10
f1 (ppm)

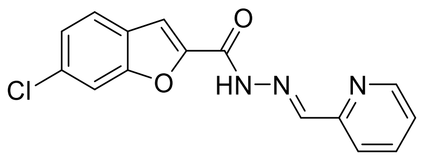

III.e

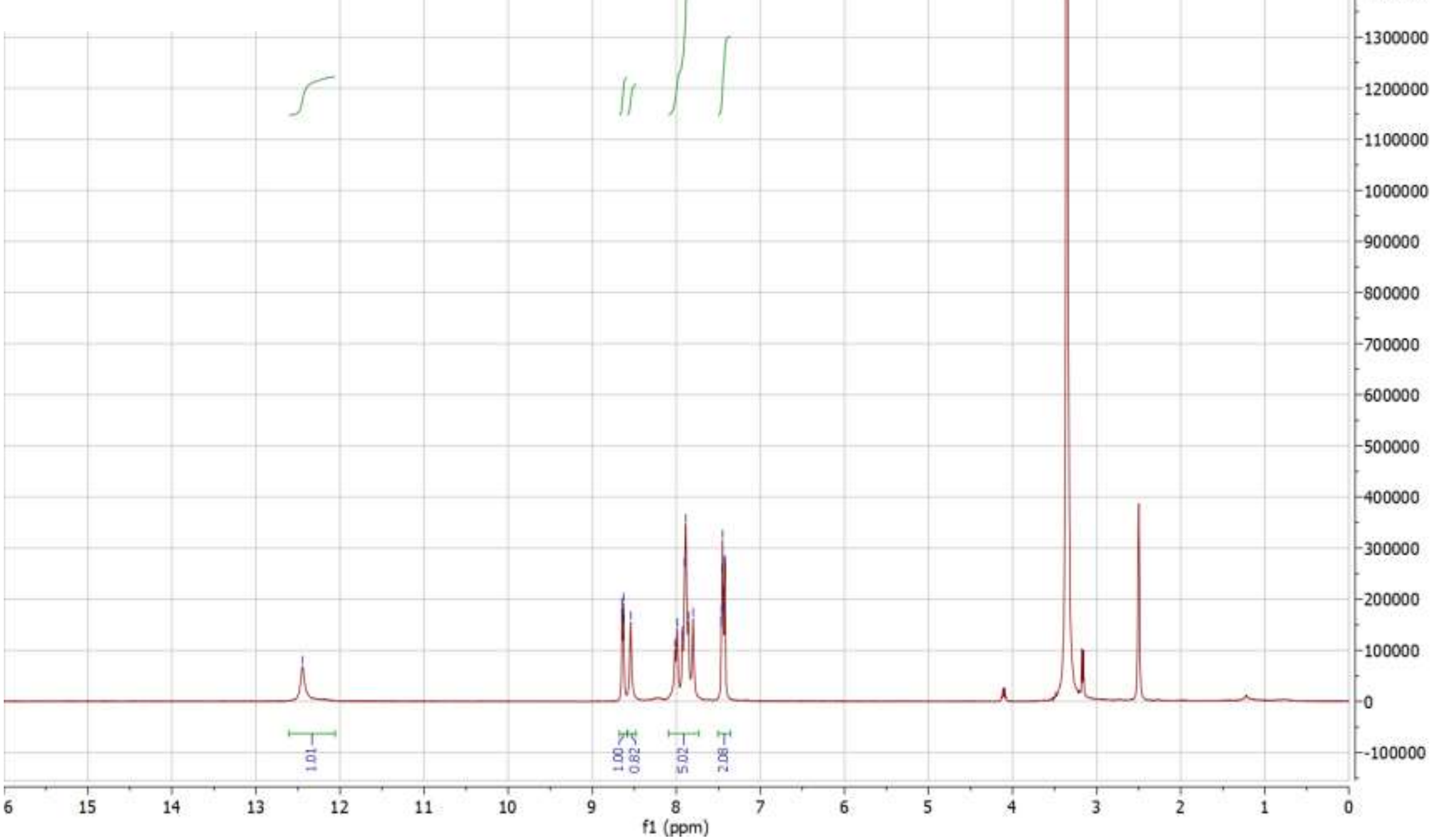
12.45
8.65 8.64 8.63 8.63 8.62 8.54 8.02 7.99 7.93 7.92 7.90 7.89 7.86 7.80 7.47 7.46 7.45 7.45 7.44 7.43 7.42
1.01
1.00
0.82
5.02
2.08
f1 (ppm)

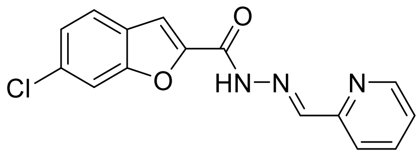
O
Cl
O
HN–N
N

III.e

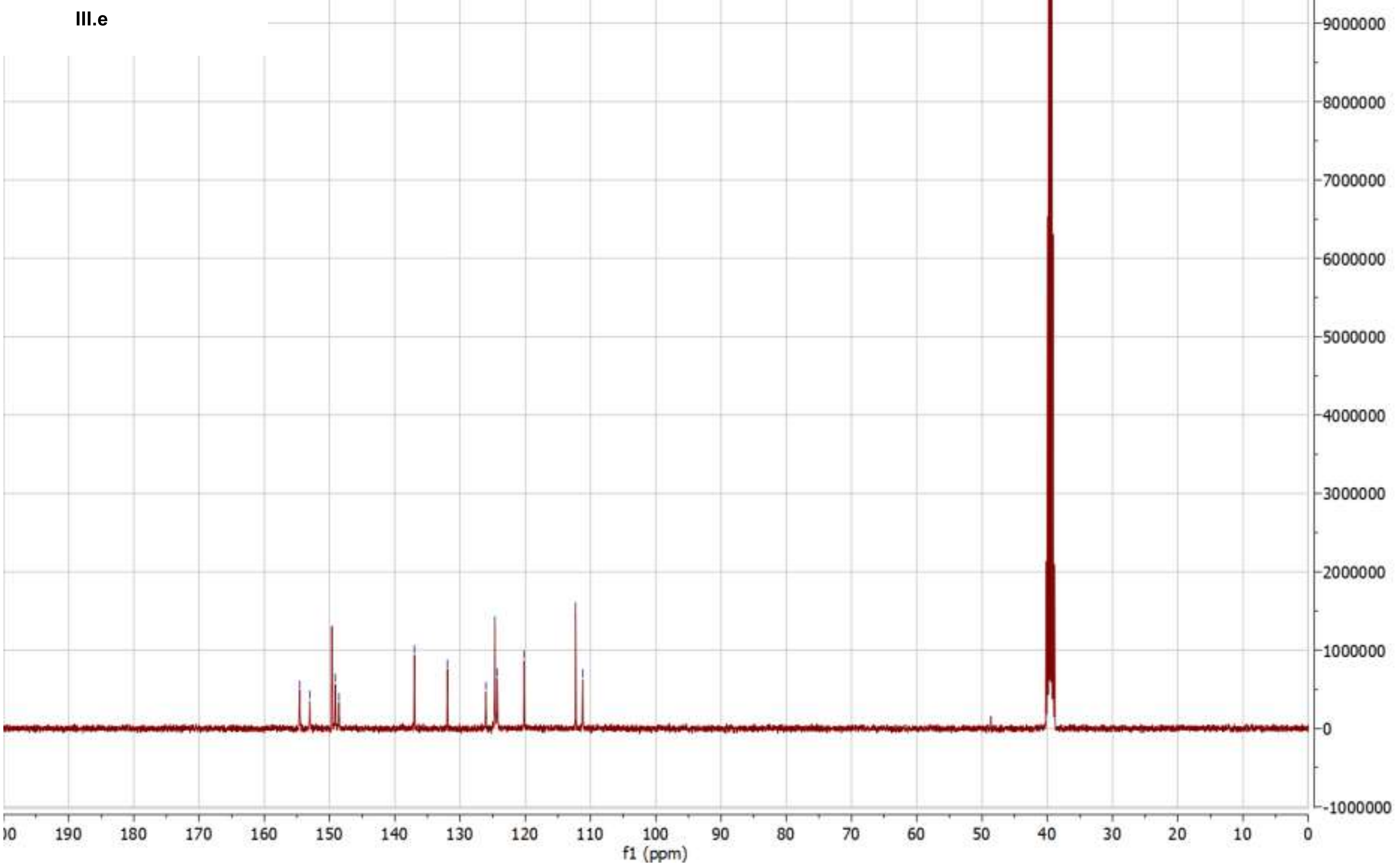
154.59
153.05
149.11
148.57
137.00
131.93
126.04
124.67
124.27
120.17
112.29
111.20
f1 (ppm)

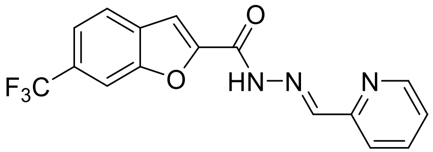

III.f

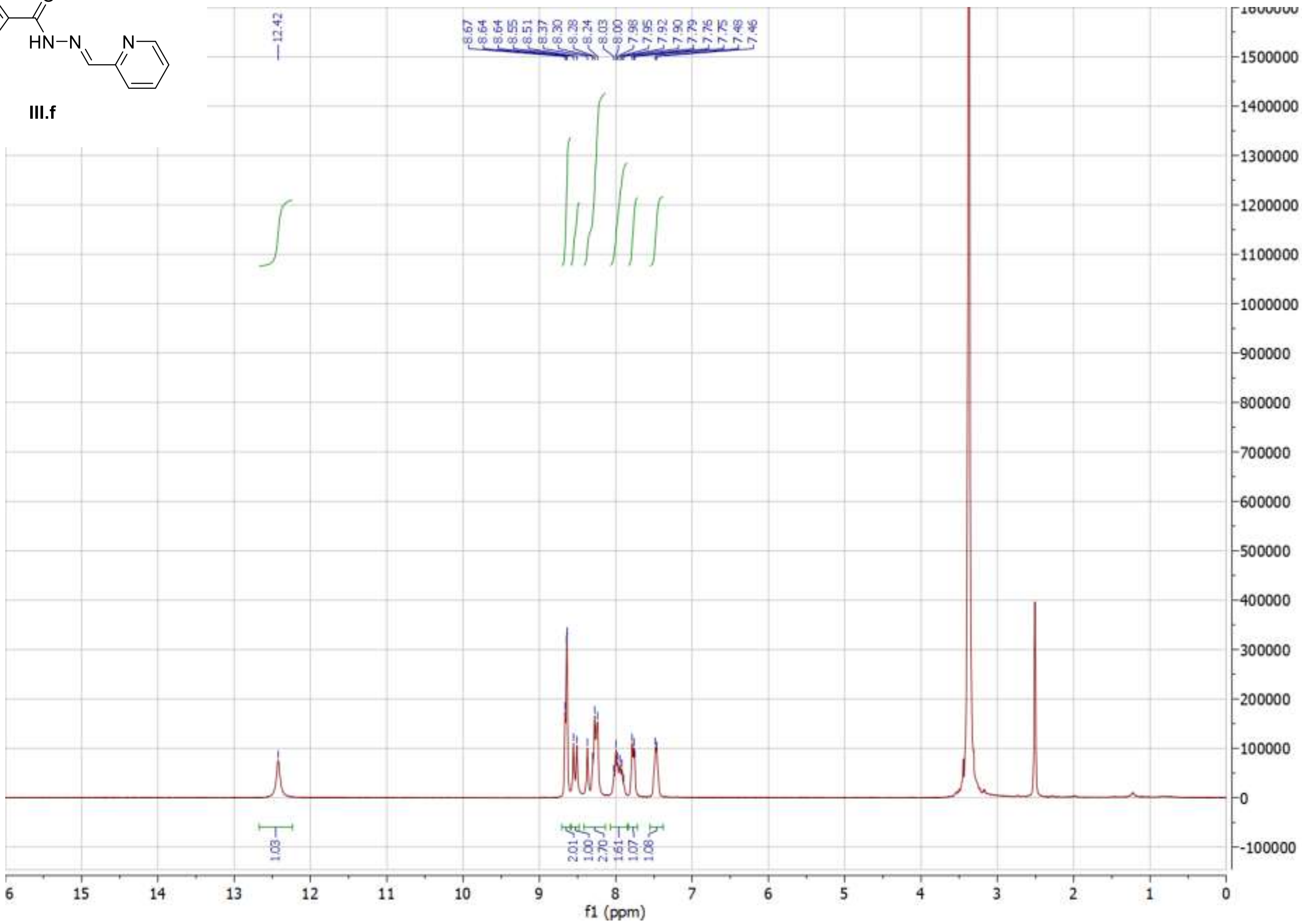

12.42
8.67
8.64
8.64
8.55
8.51
8.37
8.30
8.28
8.24
8.03
8.00
7.98
7.95
7.92
7.90
7.79
7.76
7.75
7.48
7.46
1.03
2.01
1.00
2.70
1.61
1.07
1.08
f1 (ppm)

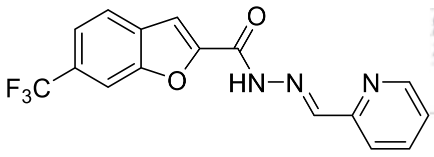
O
HN–N
N
F3C
O

III.f

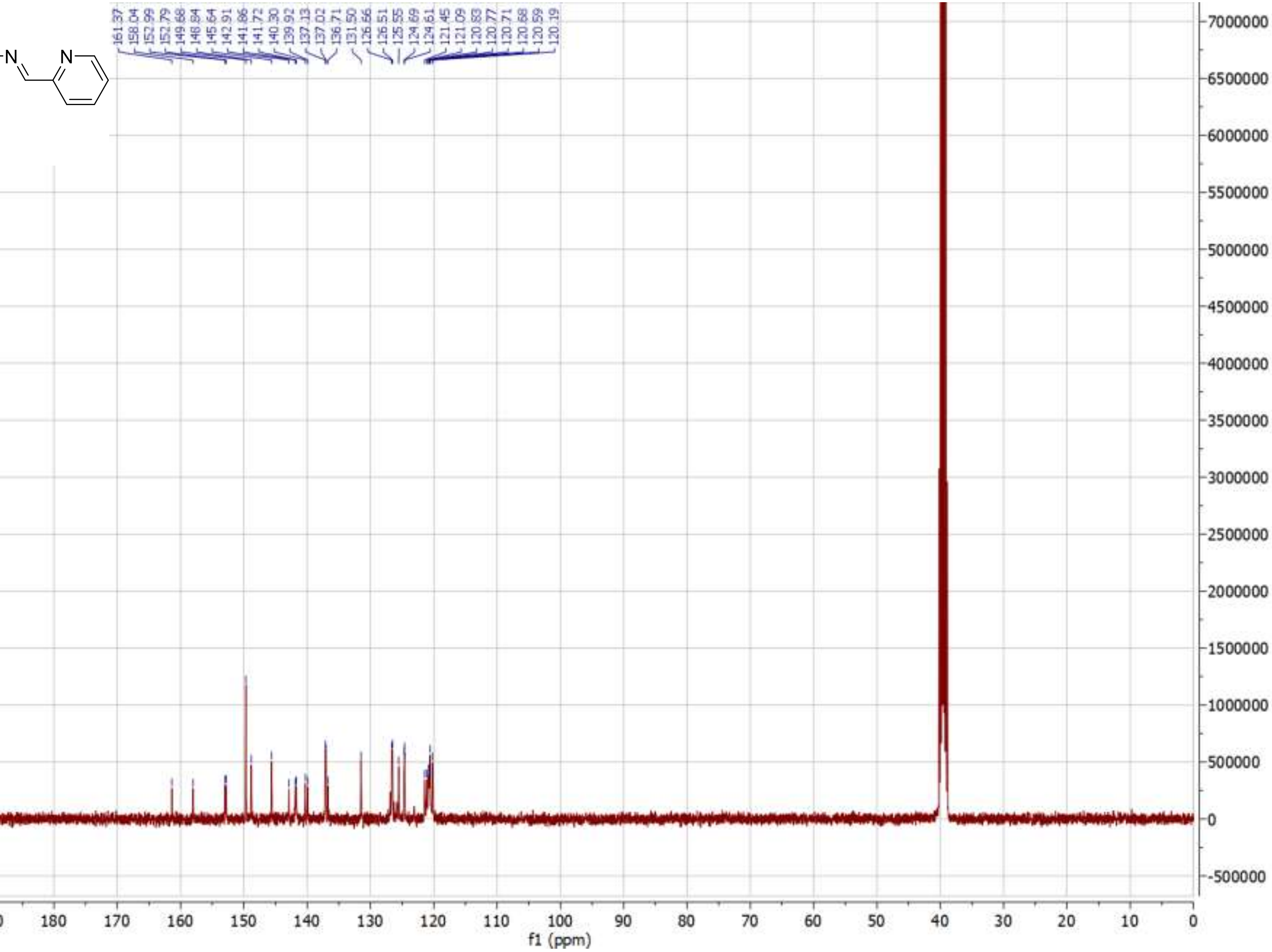
161.37
158.04
152.99
152.79
149.68
148.84
145.64
142.91
141.86
141.72
140.30
139.92
137.13
137.02
136.71
131.50
126.66
126.51
125.55
124.69
124.61
121.45
121.09
120.83
120.77
120.71
120.68
120.59
120.19
7000000
6500000
6000000
5500000
5000000
4500000
4000000
3500000
3000000
2500000
2000000
1500000
1000000
500000
0
-500000
190 180 170 160 150 140 130 120 110 100 90 80 70 60 50 40 30 20 10 0
f1 (ppm)
